# Supplementary material for: Investigating the shared genetic architecture between multiple sclerosis and inflammatory bowel diseases
Source: Nat Commun. 2021 Sep 24;12:5641. doi: 10.1038/s41467-021-25768-0 (PMC8463615; doi:10.1038/s41467-021-25768-0)
Supplement: Supplementary file 1 — Supplementary Information [file 41467_2021_25768_MOESM1_ESM.pdf]

Supplementary Information

Investigating the shared genetic architecture between multiple sclerosis and inflammatory bowel diseases

Yuanhao Yang[1,2], Hannah Musco[1], Steve Simpson-Yap[3,4], Zhihong Zhu[2,5], Ying Wang[1,2], Xin Lin[3], Jiawei Zhang[6], Bruce Taylor[3,*], Jacob Gratten[1,2,*], Yuan Zhou[3,*]

[1] Mater Research, Translational Research Institute, Brisbane, QLD, Australia

[2] Institute for Molecular Bioscience, The University of Queensland, Brisbane, QLD, Australia

[3] Menzies Institute for Medical Research, University of Tasmania, Hobart, TAS, Australia

[4] Neuroepidemiology Unit, Melbourne School of Population & Global Health, The University of Melbourne, Melbourne, VIC, Australia

[5] National Centre for Register-based Research, Aarhus University, Aarhus, Denmark

[6] Department of General Surgery, the First Affiliated Hospital of Anhui Medical University, Hefei, China

* These authors jointly supervised this work

Correspondence: Dr Yuan Zhou, email: yuan.zhou@utas.edu.au; Dr Jacob Gratten, email: jacob.gratten@mater.uq.edu.au; Professor Bruce Taylor, email: bruce.taylor@utas.edu.au.

Table of Contents

Supplementary Note 4
Table S1. Genetic correlations between MS and each of IBD, UC, and CD 6
Table S2. Summary of local genetic correlations between MS and each of IBD, UC, and CD in the MHC region 7
Table S3. Novel genetic variants associated with cross-trait MS and IBD (or UC or CD) revealed by MTAG and CPASSOC 8
Table S4. Summary of two-sample MR results between MS and each of IBD, UC, and CD 9
Table S5. Summary of two-sample MR results (excluding SNPs in the MHC region) between MS and each of IBD, UC, and CD 10
Table S6. Summary of CAUSE results between MS and each of IBD, UC, and CD 11
Table S7. Summary of CAUSE results (excluding SNPs in the MHC region) between MS and each of IBD, UC, and CD 12
Table S8. Summary of enrichment correlations among MS, IBD, UC, and CD for GTEx tissues and cell types (by tissues) 13
Table S9. Summary of significant SMR associations (SMR *p*-value $<5.28\times10^{-7}$) involving novel SNPs associated with cross-trait MS and IBD (or UC or CD); shown in Table S3 14
Figure S1. Overview of statistical analyses performed in the study 15
Figure S2. Local genetic correlations between MS and IBD revealed by ρ-HESS 16
Figure S3. Local genetic correlations between MS and UC revealed by ρ-HESS. 17
Figure S4. Local genetic correlations between MS and CD revealed by ρ-HESS. 18
Figure S5. Comparison of negative log10 *p*-values for cross-trait GWAS meta-analyses of MS-IBD, MS-UC and MS-CD performed using MTAG and CPASSOC 19
Figure S6. GSMR effect size plots for associations between MS and each of IBD, UC and CD. 20
Figure S7. Proportion of overlap of the top 10% most specific genes among 37 GTEx tissues using the Bryois et al. (2020) method. 21
Figure S8. Distribution of the proportion of total expression per gene in each of 37 GTEx tissues using the Bryois et al. (2020) method. 22
Figure S9. S-LDSC-based heritability enrichment estimates in 37 GTEx tissues for MS and each of IBD, UC and CD, using the Bryois et al. (2020) method. 23
Figure S10. MAGMA-based heritability enrichment estimates in 37 GTEx tissues for MS and each of IBD, UC and CD, without genes in the MHC region. 24
Figure S11. MAGMA-based heritability enrichment estimates in 37 GTEx tissues for MS and each of IBD, UC and CD, including genes in the MHC region. 25
Figure S12. Tissue-specific enrichment of heritability in MS, IBD, UC and CD in immune tissues using MAGMA, without genes in the MHC region. 26
Figure S13. Tissue-specific enrichment of heritability in MS, IBD, UC and CD in immune tissues using MAGMA, including genes in the MHC region.. 27
Figure S14. Proportion of overlap of the top 10% highly expressed genes among 45 GTEx tissues using the Finucane et al. (2018) method. 28
Figure S15. S-LDSC-based heritability enrichment estimates in 45 GTEx tissues for MS and each of IBD, UC and CD, using the Finucane et al. (2018) method. 29
Figure S16. Proportion of overlap of the top 10% most specific genes among 28 lung cell types, using the Bryois et al. (2020) method. 30
Figure S17. Distribution of the proportion of total expression per gene in each of the 28 lung cell types, using the Bryois et al. (2020) method. 31
Figure S18. S-LDSC-based heritability enrichment estimates in 28 lung cell types for MS and each of IBD, UC and CD. 32
Figure S19. MAGMA-based heritability enrichment in 28 lung cell types for MS and each of IBD, UC and CD, without genes in the MHC region. 33

Figure S20. MAGMA-based heritability enrichment estimates in 28 lung cell types for MS and each of IBD, UC and CD, including genes in the MHC region. 34
Figure S21. Proportion of overlap of the top 10% most specific genes among 11 PBMC cell types, using the Bryois et al. (2020) method. 35
Figure S22. Distribution of the proportion of total expression per gene in each of the 11 PBMC cell types, using the Bryois et al. (2020) method. 36
Figure S23. S-LDSC-based heritability enrichment estimates in 11 PBMC cell types for MS and each of IBD, UC and CD. 37
Figure S24. MAGMA-based heritability enrichment in 11 PBMC cell types for MS and each of IBD, UC and CD, without genes in the MHC region. 38
Figure S25. MAGMA-based heritability enrichment estimates in 11 PBMC cell types for MS and each of IBD, UC and CD, including genes in the MHC region. 39
Figure S26. Proportion of overlap of the top 10% most specific genes among 30 spleen cell types, using the Bryois et al. (2020) method. 40
Figure S27. Distribution of the proportion of total expression per gene in each of the 30 spleen cell types, using the Bryois et al. (2020) method. 41
Figure S28. S-LDSC-based heritability enrichment estimates in 30 spleen cell types for MS and each of IBD, UC and CD. 42
Figure S29. MAGMA-based heritability enrichment in 30 spleen cell types for MS and each of IBD, UC and CD, without genes in the MHC region. 43
Figure S30. MAGMA-based heritability enrichment estimates in 30 spleen cell types for MS and each of IBD, UC and CD, including genes in the MHC region. 44
Figure S31. Proportion of overlap of the top 10% most specific genes among 15 small intestine cell types, using the Bryois et al. (2020) method. 45
Figure S32. Distribution of the proportion of total expression per gene in each of the 15 small intestine cell types, using the Bryois et al. (2020) method. 46
Figure S33. S-LDSC-based heritability enrichment estimates in 15 small intestine cell types for MS and each of IBD, UC and CD. 47
Figure S34. MAGMA-based heritability enrichment in 15 small intestine cell types for MS and each of IBD, UC and CD, without genes in the MHC region. 48
Figure S35. MAGMA-based heritability enrichment estimates in 15 small intestine cell types for MS and each of IBD, UC and CD, including genes in the MHC region. 49
Figure S36. Cell type-specific enrichment of heritability for MS, IBD, UC and CD in immune tissue cells using MAGMA, without genes in the MHC region. 50
Figure S37. Cell type-specific enrichment of heritability for MS, IBD, UC and CD in immune tissue cells using MAGMA, including genes in the MHC region. 51
Figure S38. Comparison of heritability enrichment estimates. 52
Figure S39. Tissue-specific enrichment of heritability for MS, IBD, UC and CD in immune tissues, using S-LDSC. 53
Figure S40. Cell type-specific enrichment of heritability for MS, IBD, UC and CD in immune tissue cells, using S-LDSC. 54
Figure S41. Manhattan plots of SMR results for associations between gene expression in lung and each of MS, IBD, UC and CD. 55
Figure S42. Manhattan plots of SMR results for associations between gene expression in small intestine-terminal ileum and each of MS, IBD, UC and CD. 56
Figure S43. Manhattan plots of SMR results for associations between gene expression in spleen and each of MS, IBD, UC and CD. 57
Figure S44. Manhattan plots of SMR results for associations between gene expression in whole blood and each of MS, IBD, UC and CD. 58
Reference 59

Supplementary Note

Tissue-specific SNP heritability enrichment using the Finucane et al. (2018) method

Our primary stratified linkage disequilibrium score regression (S-LDSC) analyses of tissue-specific SNP heritability relied on the method in Bryois et al. (2020)[1] to define tissue annotations based on tissue-specific genes. As a sensitivity analyses, we performed a parallel series of S-LDSC analyses in which we applied the original method from Finucane et al. (2018)[2] for selecting the top 10% highly expressed genes per tissue. We excluded tissues that were non-natural tissues, as well as testis and tissues with <100 samples. Here we did not combine the tissues from the same organ and a total of 45 Genotype-Tissue Expression (GTEx) tissues remained. We then calculated *T*-statistics per gene for a focal tissue as a proxy, to measure the expression levels in this focal tissue compared to expression levels in all other GTEx tissues ('control' tissues), excepting the tissues from the same tissue type (e.g., brain-related tissues were excluded from 'control' tissues if the focal tissue was another brain tissue). *T*-statistics were calculated using linear models via ordinary least-squares. Tissue type was re-coded to '1' for the focal tissue and '-1' for all the control tissues and used as a covariate in the linear model. Both age and sex were also included as covariates. Only protein-coding genes were analysed in the analyses. The top 10% highly expressed genes for each GTEx tissue were then selected for downstream analyses by ranking the T-statistics. In these analyses, we observed false discovery rate (FDR) significant single nucleotide polymorphism (SNP) heritability enrichments in both multiple sclerosis (MS) and at least one of inflammatory bowel disease (IBD), ulcerative colitis (UC), or Crohn's disease (CD) in lung, small intestine–terminal ileum, spleen, and whole blood (Figure S14, Supplementary Data 3), which is similar to what we found using the Bryois et al. 2020[1] method (Figure S8, Supplementary Data 2).

Gene-set enrichment analysis using MAGMA

We conducted gene-set enrichment analysis using MAGMA (Multi-marker Analysis of GenoMic Annotation)[3], as a sensitivity analysis for S-LDSC. MAGMA implements gene-set enrichment analysis in two steps. First, gene-level association analyses are performed based on the genome-wide association study (GWAS) summary data, which estimates the association between a gene and phenotype by averaging *p*-values of the SNPs located near the target gene. LD structure is corrected for according to the 1000 Genomes Europeans reference. Second, gene-set enrichment analysis is performed for a given gene set (e.g. the 10% highly specific genes for GTEx tissues), using a linear regression for the gene-level association results (i.e. *Z*-

score) adjusted by the covariates including gene size, gene density, inverse minor allele counts per gene, and the logarithm of these covariates. In our study, we performed MAGMA gene-set enrichment analysis for 121 gene sets (37 GTEx tissues and 84 cell types) that were assessed in the S-LDSC using the Bryois et al. (2020)[1] method.

To investigate the influence of the major histocompatibility complex (MHC) region on heritability enrichment, we also conducted parallel MAGMA gene-set enrichment analyses with genes located in the MHC region included. Also, to explore if significant tissue-level SNP heritability enrichments were affected by overlap of genes among tissues/cell types, we further performed conditional MAGMA analyses with and without the MHC genes. The conditional MAGMA is also based on the similar linear regression, adjusted by the additional gene sets which are same as the scheme described in our S-LDSC analyses.

As summarised in Figure S11-12, we identified FDR- or Bonferroni- significant heritability enrichment in MS, IBD, UC and CD in lung (with the exception of UC), spleen, and whole blood. MS was also associated with FDR-significant enrichment in small intestine–terminal ileum. After conditional analyses, MS showed FDR-significant enrichments for all four tissues; and the enrichments for lung and whole blood remained FDR-significant for IBD and CD.

Patterns of cell type-specific enrichment using MAGMA (Figure S35-36) were also generally similar to those observed using S-LDSC. A large proportion of the FDR-significant cell types identified in S-LDSC were also FDR-significant using MAGMA, including $CD8^+$ cytotoxic T cells in spleen for MS and CD in the conditional MAGMA analysis. Exceptions included two small intestine cells for MS, activated dendritic cells in lung for IBD and CD, $CD4^+$ T cells in lung for IBD and UC, $CD56^+$ NK cells for CD, and $CD8^+$ cytotoxic T cells and NK cells in lung as well as three spleen cells for IBD. These inconsistent results are likely due to the conservative estimation process of MAGMA, as described in Bryois et al. 2020[1]. Overall, most of these MAGMA results were similar to the S-LDSC results (Figure S37), suggesting that our S-LDSC results are reliable. For this reason, we present the S-LDSC results as our main results because we are interested in identifying tissue- and cell type-specific SNP heritability enrichments for MS and IBDs. As expected, tissue- and cell type-specific enrichments slightly increased with the inclusion of genes in the MHC region (Supplementary Data 7-8), consistent with a modest contribution of the MHC region to the SNP heritability of MS and IBDs.

Table S1. Genetic correlations between MS and each of IBD, UC, and CD

Trait 1–Trait 2	MS–IBD	MS–UC	MS–CD	UC–CD
No. overlapped SNPs	1,170,899	1,170,881	1,169,137	1,180,651
Without constrained intercept				
r_g	0.28	0.33	0.16	0.7
standard error (r_g)	0.01	0.05	0.05	0.05
p-value	2.01×10^{-10}	1.66×10^{-13}	2.40×10^{-3}	2.05×10^{-47}
Intercept (genetic covariance)	0.1	0.08	0.08	0.18
$\boldsymbol{\lambda_{GC}}$ (Trait 1)	1.15	1.15	1.15	1.13
Intercept (Trait 1)	1.02	1.02	1.02	1.05
$\boldsymbol{\lambda_{GC}}$ (Trait 2)	1.17	1.13	1.14	1.14
Intercept (Trait 2)	1.05	1.05	1.02	1.02
With constrained intercept of heritability				
r_g	0.24	0.27	0.14	0.56
standard error (r_g)	0.04	0.04	0.05	0.04
p-value	9.81×10^{-11}	1.75×10^{-13}	1.90×10^{-3}	7.32×10^{-37}
Intercept (genetic covariance)	0.1	0.08	0.08	0.17
$\boldsymbol{\lambda_{GC}}$ (Trait 1)	1.15	1.15	1.15	1.13
Intercept (Trait 1)	1	1	1	1
$\boldsymbol{\lambda_{GC}}$ (Trait **2**)	1.17	1.13	1.14	1.14
Intercept (Trait 2)	1	1	1	1

*The genetic correlation between multiple sclerosis (MS) and ulcerative colitis (UC) is statistically different from that between MS and Crohn's disease (CD) at 5% significance level (without constrained intercept: *Z*-score=2.39, *p*=0.02 [two-tailed *Z* test]; constrained intercept: *Z*-score=1.97, *p*=0.05 [two-tailed *Z* test]). SNP: single nucleotide polymorphisms. r_g: genetic correlation.

Table S2. Summary of local genetic correlations between MS and each of IBD, UC, and CD in the MHC region

Trait 1–Trait 2	MHC regions (chromosome: BP start–BP end)	Number of SNPs	local rhog	*p*-value (local rhog)	h^2 (Trait 1)	*p*-value (h^2 [Trait 1])	h^2 (Trait 2)	*p*-value (h^2 [Trait 2])	local r_g
MS–UC	chr6: 28017819–28917608	216	-2.79×10^{-4}	0.44	4.07×10^{-3}	4.69×10^{-21}	8.16×10^{-4}	4.19×10^{-5}	-0.15
MS–UC	chr6: 28917608–29737971	31	-1.90×10^{-3}	1.00×10^{-5}	6.37×10^{-3}	2.59×10^{-34}	8.76×10^{-4}	NA	-0.80
MS–UC	chr6: 30798168–31571218	131	2.45×10^{-3}	3.74×10^{-3}	0.024	6.90×10^{-118}	3.26×10^{-3}	1.02×10^{-8}	0.28
MS–UC	chr6: 31571218–32682664	119	0.016	8.68×10^{-35}	0.055	1.46×10^{-265}	7.50×10^{-3}	1.52×10^{-17}	0.77
MS–UC	chr6: 32682664–33236497	184	3.42×10^{-3}	7.66×10^{-6}	0.020	2.68×10^{-96}	1.90×10^{-3}	5.73×10^{-5}	0.55
MS–CD	chr6: 28017819–28917608	215	3.77×10^{-4}	0.31	4.04×10^{-3}	6.26×10^{-20}	1.66×10^{-4}	NA	0.46
MS–CD	chr6: 28917608–29737971	28	8.90×10^{-4}	0.058	6.79×10^{-3}	1.06×10^{-34}	5.64×10^{-4}	NA	0.45
MS–CD	chr6: 30798168–31571218	119	-2.75×10^{-3}	2.54×10^{-3}	0.025	1.53×10^{-116}	3.08×10^{-3}	4.70×10^{-10}	-0.31
MS–CD	chr6: 31571218–32682664	106	1.26×10^{-4}	0.92	0.047	1.08×10^{-216}	2.24×10^{-3}	6.80×10^{-11}	0.01
MS–CD	chr6: 32682664–33236497	176	-2.54×10^{-4}	0.76	0.021	1.39×10^{-95}	1.59×10^{-3}	1.60×10^{-5}	-0.04
MS–IBD	chr6: 28017819–28917608	216	8.17×10^{-5}	0.78	4.06×10^{-3}	4.69×10^{-21}	3.35×10^{-4}	0.022	0.07
MS–IBD	chr6: 28917608–29737971	31	-9.64×10^{-4}	7.21×10^{-3}	6.37×10^{-3}	2.59×10^{-34}	6.54×10^{-4}	3.99×10^{-63}	-0.47
MS–IBD	chr6: 30798168–31571218	131	4.46×10^{-4}	0.53	0.024	6.90×10^{-118}	3.05×10^{-3}	3.75×10^{-10}	0.05
MS–IBD	chr6: 31571218–32682664	119	0.011	1.48×10^{-25}	0.055	1.46×10^{-265}	6.51×10^{-3}	1.84×10^{-20}	0.58
MS–IBD	chr6: 32682664–33236497	184	2.01×10^{-3}	1.71×10^{-3}	0.020	2.68×10^{-96}	2.21×10^{-3}	6.38×10^{-7}	0.30

MS: multiple sclerosis. IBD: inflammatory bowel disease. UC: ulcerative colitis. CD: Crohn's disease. SNP: single nucleotide polymorphisms. MHC: major histocompatibility complex. BP: base pair. h^2: heritability. r_g: genetic correlation.

Table S3. Novel genetic variants associated with cross-trait MS and IBD (or UC or CD) revealed by MTAG and CPASSOC

SNP ID	rs13428812	rs116555563	rs13428812	rs9977672
Chromosome	2	5	2	21
BP Position	25492467	158503209	25492467	40463283
Effect Allele	G	T	G	A
Non-effect Allele	A	C	A	G
Nearest Gene	*DNMT3A*	*EBF1*	*DNMT3A*	*DSCAM-AS1*
Trait 1	MS	MS	MS	MS
Trait 2	IBD	UC	CD	CD
Trait 1 beta	0.08	-0.52	0.08	-0.09
Trait 1 *p*-value	3.19×10^{-6}	2.93×10^{-6}	3.19×10^{-6}	1.45×10^{-6}
Trait 2 beta	0.09	-0.52	0.12	-0.13
Trait 2 *p*-value	1.86×10^{-6}	1.21×10^{-5}	3.93×10^{-7}	2.53×10^{-6}
MTAG beta	0.06	-0.09	0.06	-0.06
MTAG *p*-value	6.84×10^{-11}	3.64×10^{-10}	2.67×10^{-11}	6.32×10^{-11}
CPASSOC *p*-value	9.99×10^{-9}	3.54×10^{-9}	2.80×10^{-9}	2.85×10^{-9}

MS: multiple sclerosis. IBD: inflammatory bowel disease. UC: ulcerative colitis. CD: Crohn's disease. SNP: single nucleotide polymorphisms. BP: base pair. MTAG: Multi-Trait Analysis of genome-wide association study (GWAS). CPASSOC: Cross Phenotype Association.

Table S4. Summary of two-sample MR results between MS and each of IBD, UC, and CD

Method	No of SNPs[1]	Beta (logit)[2]	Beta (liability)[2]	OR (liability)[2]	*P*-value
**MS to IBD**					
GSMR	75 (59)	0.17 (0.14–0.20)	0.17 (0.14–0.20)	1.19 (1.15–1.22)	1.69×10^{-29}
MR-Egger	75	0.19 (0.06–0.33)	0.19 (0.06–0.33)	1.21 (1.06–1.38)	6.62×10^{-3}
IVW	75	0.14 (0.06–0.22)	0.14 (0.06–0.21)	1.15 (1.07–1.24)	3.18×10^{-4}
Weighted Median	75	0.16 (0.10–0.21)	0.16 (0.10–0.21)	1.17 (1.11–1.23)	7.78×10^{-9}
Weighted Mode	75	0.17 (0.13–0.21)	0.17 (0.13–0.21)	1.18 (1.13–1.23)	8.58×10^{-12}
**IBD to MS**					
GSMR	68 (51)	0.03 (-0.01–0.06)	0.02 (-0.01–0.06)	1.02 (0.99–1.06)	0.14
MR-Egger	68	0.30 (-0.02–0.63)	0.29 (-0.02–0.61)	1.34 (0.98–1.84)	0.070
IVW	68	0.02 (-0.09–0.14)	0.02 (-0.09–0.13)	1.02 (0.92–1.14)	0.67
Weighted Median	68	0.02 (-0.04–0.08)	0.02 (-0.04–0.08)	1.02 (0.97–1.08)	0.45
Weighted Mode	68	0.02 (-0.05–0.10)	0.02 (-0.04–0.09)	1.02 (0.96–1.10)	0.50
**MS to UC**					
GSMR	75 (62)	0.17 (0.13–0.20)	0.17 (0.13–0.20)	1.18 (1.13–1.23)	7.35×10^{-16}
MR-Egger	75	0.31 (0.18–0.45)	0.31 (0.18–0.44)	1.37 (1.20–1.56)	1.65×10^{-5}
IVW	75	0.19 (0.12–0.27)	0.19 (0.11–0.27)	1.21 (1.12–1.31)	8.69×10^{-7}
Weighted Median	75	0.19 (0.12–0.26)	0.19 (0.12–0.25)	1.20 (1.12–1.29)	1.50×10^{-7}
Weighted Mode	75	0.18 (0.09–0.28)	0.18 (0.09–0.27)	1.20 (1.10–1.32)	1.91×10^{-4}
**UC to MS**					
GSMR	42 (31)	0.08 (0.04–0.12)	0.08 (0.04–0.12)	1.08 (1.04–1.12)	2.30×10^{-5}
MR-Egger	42	0.33 (-0.25–0.92)	0.33 (-0.26–0.92)	1.40 (0.77–2.52)	0.27
IVW	42	0.21 (0.03–0.38)	0.21 (0.03–0.38)	1.23 (1.03–1.47)	0.021
Weighted Median	42	0.05 (-0.02–0.11)	0.05 (-0.02–0.11)	1.05 (0.98–1.11)	0.15
Weighted Mode	42	0.06 (-0.01–0.12)	0.06 (-0.01–0.12)	1.06 (0.99–1.13)	0.12
**MS to CD**					
GSMR	75 (58)	0.07 (0.03–0.11)	0.07 (0.03–0.11)	1.07 (1.03–1.11)	3.51×10^{-4}
MR-Egger	75	0.04 (-0.13–0.21)	0.04 (-0.13–0.21)	1.04 (0.88–1.23)	0.67
IVW	75	0.07 (-0.03–0.17)	0.07 (-0.03–0.16)	1.07 (0.97–1.18)	0.17
Weighted Median	75	0.06 (0–0.12)	0.06 (0–0.12)	1.06 (1–1.13)	0.051
Weighted Mode	75	0.04 (-0.01–0.10)	0.04 (-0.01–0.10)	1.04 (0.99–1.10)	0.13
**CD to MS**					
GSMR	56 (41)	-0.04 (-0.07–-0.01)	-0.04 (-0.07–-0.01)	0.96 (0.93–0.99)	4.17×10^{-3}
MR-Egger	56	0.17 (-0.02–0.36)	0.17 (-0.02–0.36)	1.19 (0.98–1.44)	0.089
IVW	56	-0.01 (-0.09–0.07)	-0.01 (-0.09–0.07)	0.99 (0.92–1.07)	0.81
Weighted Median	56	0.01 (-0.03–0.06)	0.01 (-0.03–0.06)	1.01 (0.97–1.06)	0.61
Weighted Mode	56	0.01 (-0.04–0.07)	0.01 (-0.04–0.07)	1.01 (0.96–1.07)	0.63

[1]Numbers in brackets are 95% confidence intervals (CIs) of the estimates. [2]Numbers in the brackets of GSMR results are the numbers of SNPs remaining after the HEIDI (HEterogeneity In Dependent Instrument) test. MS: multiple sclerosis. IBD: inflammatory bowel disease. UC: ulcerative colitis. CD: Crohn's disease. SNP: single nucleotide polymorphisms. OR: odds ratio. MR: Mendelian Randomisation. GSMR: Generalised Summary-data-based Mendelian Randomisation. IVW: inverse variance weighting.

Table S5. Summary of two-sample MR results (excluding SNPs in the MHC region) between MS and each of IBD, UC, and CD

Method	No of SNPs[1]	Beta (logit)[2]	Beta (liability)[2]	OR (liability)[2]	*P*-value
**MS to IBD**					
GSMR	70 (61)	0.12 (0.08–0.16)	0.12 (0.08–0.16)	1.13 (1.08–1.17)	2.07×10^{-9}
MR-Egger	70	0.30 (-0.15–0.76)	0.31 (-0.15–0.78)	1.37 (0.86–2.18)	0.19
IVW	70	0.13 (0.04–0.23)	0.14 (0.04–0.24)	1.15 (1.04–1.27)	6.36×10^{-3}
Weighted Median	70	0.09 (0.03–0.16)	0.09 (0.03–0.16)	1.10 (1.03–1.17)	6.16×10^{-3}
Weighted Mode	70	0.09 (-0.03–0.21)	0.09 (-0.04–0.22)	1.09 (0.97–1.24)	0.16
**IBD to MS**					
GSMR	63 (49)	0.02 (-0.01–0.06)	0.02 (-0.01–0.06)	1.02 (0.99–1.06)	0.17
MR-Egger	63	0.18 (-0.05–0.41)	0.17 (-0.05–0.40)	1.19 (0.95–1.49)	0.14
IVW	63	0.05 (-0.03–0.13)	0.05 (-0.03–0.13)	1.05 (0.97–1.14)	0.21
Weighted Median	63	0.02 (-0.04–0.08)	0.02 (-0.04–0.08)	1.02 (0.97–1.08)	0.44
Weighted Mode	63	0.02 (-0.05–0.10)	0.02 (-0.05–0.09)	1.02 (0.95–1.10)	0.56
**MS to UC**					
GSMR	70 (66)	0.10 (0.06–0.15)	0.10 (0.06–0.15)	1.11 (1.06–1.16)	1.14×10^{-5}
MR-Egger	70	0.22 (-0.20–0.64)	0.22 (-0.20–0.64)	1.25 (0.82–1.90)	0.21
IVW	70	0.13 (0.04–0.22)	0.13 (0.04–0.22)	1.14 (1.04–1.25)	3.52×10^{-3}
Weighted Median	70	0.05 (-0.02–0.13)	0.05 (-0.02–0.13)	1.06 (0.98–1.14)	0.17
Weighted Mode	70	0.01 (-0.15–0.16)	0.01 (-0.14–0.16)	1.01 (0.87–1.17)	0.93
**UC to MS**					
GSMR	36 (31)	0.08 (0.04–0.12)	0.08 (0.04–0.12)	1.08 (1.04–1.12)	2.30×10^{-5}
MR-Egger	36	0.03 (-0.26–0.33)	0.03 (-0.26–0.33)	1.03 (0.77–1.39)	0.82
IVW	36	0.10 (0.02–0.18)	0.10 (0.02–0.18)	1.10 (1.02–1.20)	0.020
Weighted Median	36	0.04 (-0.02–0.10)	0.04 (-0.02–0.10)	1.04 (0.98–0.10)	0.17
Weighted Mode	36	0.05 (-0.03–0.12)	0.05 (-0.03–0.12)	1.05 (0.97–1.13)	0.22
**MS to CD**					
GSMR	70 (57)	0.12 (0.07–0.18)	0.12 (0.07–0.17)	1.13 (1.07–1.19)	8.42×10^{-6}
MR-Egger	70	0.43 (-0.18–1.05)	0.42 (-0.18–1.03)	1.53 (0.83–2.79)	0.18
IVW	70	0.13 (0–0.26)	0.13 (0–0.26)	1.13 (1–1.29)	0.049
Weighted Median	70	0.12 (0.04–0.20)	0.12 (0.04–0.20)	1.13 (1.04–1.22)	3.72×10^{-3}
Weighted Mode	70	0.12 (-0.04–0.29)	0.12 (-0.04–0.29)	1.13 (0.96–1.33)	0.14
**CD to MS**					
GSMR	54 (40)	-0.04 (-0.07–-0.01)	-0.04 (-0.07–-0.01)	0.96 (0.93–0.99)	6.46×10^{-3}
MR-Egger	54	0.13 (-0.03–0.29)	0.13 (-0.03–0.30)	1.14 (0.97–1.35)	0.12
IVW	54	0.01 (-0.06–0.07)	0.01 (-0.06–0.08)	1.01 (0.94–1.08)	0.79
Weighted Median	54	0.01 (-0.03–0.06)	0.01 (-0.03–0.06)	1.01 (0.97–1.06)	0.59
Weighted Mode	54	0.01 (-0.04–0.06)	0.01 (-0.04–0.06)	1.01 (0.96–1.07)	0.66

[1]Numbers in brackets are 95% confidence intervals (CIs) of the estimates. [2]Numbers in the brackets of GSMR results are the numbers of SNPs remaining after the HEIDI (HEterogeneity In Dependent Instrument) test. MS: multiple sclerosis. IBD: inflammatory bowel disease. UC: ulcerative colitis. CD: Crohn's disease. MHC: major histocompatibility complex. SNP: single nucleotide polymorphisms. OR: odds ratio. MR: Mendelian Randomisation. GSMR: Generalised Summary-data-based Mendelian Randomisation. IVW: inverse variance weighting.

Table S6. Summary of CAUSE results between MS and each of IBD, UC, and CD

Model	Gamma[1]	Gamma (liability)[1]	OR (liability)[1]	*P*-value (Gamma)[2]	Eta[1]	*q*[1]	*Z*	*P*-value[3]
**MS to IBD (*N* = 2,092)**								
Sharing	NA	NA	NA	NA	0.09 (0.04–0.17)	0.26 (0.03–0.52)	-1.95	0.025
Causal	0.05 (0.02–0.07)	0.05 (0.02–0.07)	1.05 (1.02–1.07)	1.01×10^{-4}	-0.01 (-0.43–0.34)	0.03 (0–0.25)		
**IBD to MS (*N* = 2,088)**								
Sharing	NA	NA	NA	NA	0.20 (0.10–0.42)	0.27 (0.07–0.52)	-3.15	8.06×10^{-4}
Causal	0.09 (0.06–0.12)	0.09 (0.06–0.12)	1.09 (1.06–1.12)	8.37×10^{-9}	-2.63 (-3.00–-2.30)	0.03 (0.02–0.05)		
**MS to UC (*N* = 2,092)**								
Sharing	NA	NA	NA	NA	0.15 (0.10–0.21)	0.41 (0.24–0.60)	-1.01	0.16
Causal	0.09 (0.05–0.12)	0.09 (0.05–0.12)	1.09 (1.05–1.13)	6.94×10^{-7}	-0.19 (-0.56–0.22)	0.06 (0–0.25)		
**UC to MS (*N* = 1,723)**								
Sharing	NA	NA	NA	NA	3.07 (2.35–3.89)	0.02 (0.01–0.04)	-0.52	0.30
Causal	0.06 (0.03–0.10)	0.06 (0.03–0.10)	1.06 (1.03–1.11)	8.21×10^{-4}	-2.21 (-2.67–-1.80)	0.02 (0.01–0.04)		
**MS to CD (*N* = 2,092)**								
Sharing	NA	NA	NA	NA	-0.05 (-0.42–0.53)	0.04 (0–0.28)	-0.68	0.25
Causal	-0.01 (-0.02–0)	-0.01 (-0.02–0)	0.99 (0.98–1.00)	8.25×10^{-1}	-0.01 (-0.43–0.35)	0.02 (0–0.23)		
**CD to MS (*N* = 1,805)**								
Sharing	NA	NA	NA	NA	-2.53 (-3.09–-1.83)	0.04 (0.02–0.05)	-1.18	0.12
Causal	0.04 (0.01–0.07)	0.04 (0.01–0.07)	1.04 (1.01–1.07)	8.96×10^{-3}	-2.47 (-3.04–-1.76)	0.04 (0.02–0.06)		

[1]Numbers in brackets are 95% confidence intervals (CIs) of the estimates. [2]The *p*-value is calculated from the estimated gamma and its 95% CI, assuming the estimates of Gamma are nearly normal distributed. [3]*P*-value for model comparison using the expected log pointwise posterior density. The second model is a better fit if *Z*-score is less than 0. MS: multiple sclerosis. IBD: inflammatory bowel disease. UC: ulcerative colitis. CD: Crohn’s disease. SNP: single nucleotide polymorphisms. CAUSE: Causal Analysis Using Summary Effect Estimates.

Table S7. Summary of CAUSE results (excluding SNPs in the MHC region) between MS and each of IBD, UC, and CD

Model	Gamma[1]	Gamma (liability)[1]	OR (liability)[1]	*P*-value (Gamma)[2]	Eta[1]	*q*[1]	*Z*	*P*-value[3]
**MS to IBD (*N* = 1,771)**								
Sharing	NA	NA	NA	NA	0.23 (0.09–0.50)	0.22 (0.05–0.50)	-2.59	4.80×10^{-3}
Causal	0.08 (0.04–0.11)	0.08 (0.04–0.11)	1.09 (1.04–1.12)	9.53×10^{-6}	0.05 (-0.52–0.70)	0.03 (0–0.24)		
**IBD to MS (*N* = 1,939)**								
Sharing	NA	NA	NA	NA	0.19 (0.08–0.37)	0.27 (0.05–0.52)	-2.21	0.014
Causal	0.08 (0.04–0.11)	0.08 (0.04–0.11)	1.08 (1.04–1.11)	9.53×10^{-6}	0.03 (-0.47–0.65)	0.03 (0–0.25)		
**MS to UC (*N* = 1,771)**								
Sharing	NA	NA	NA	NA	0.18 (0.10–0.32)	0.33 (0.13–0.58)	-2.47	6.81×10^{-3}
Causal	0.08 (0.05–0.12)	0.08 (0.05–0.12)	1.08 (1.05–1.13)	9.53×10^{-6}	0.02 (-0.55–0.61)	0.03 (0–0.24)		
**UC to MS (*N* = 1,552)**								
Sharing	NA	NA	NA	NA	0.14 (-0.24–0.65)	0.07 (0–0.36)	-1.54	0.062
Causal	0.05 (0.02–0.09)	0.05 (0.02–0.09)	1.05 (1.02–1.09)	5.15×10^{-3}	0.01 (-0.54–0.58)	0.03 (0–0.24)		
**MS to CD (*N* = 1,771)**								
Sharing	NA	NA	NA	NA	0.15 (-0.25–0.73)	0.08 (0–0.37)	-1.53	0.062
Causal	0.06 (0.02–0.09)	0.06 (0.02–0.09)	1.06 (1.02–1.09)	8.21×10^{-4}	0.01 (-0.68–0.70)	0.03 (0–0.24)		
**CD to MS (*N* = 1,705)**								
Sharing	NA	NA	NA	NA	0.12 (-0.11–0.58)	0.10 (0–0.40)	-1.87	0.030
Causal	0.05 (0.02–0.08)	0.05 (0.02–0.08)	1.05 (1.02–1.08)	1.13×10^{-3}	0.02 (-0.43–0.57)	0.03 (0–0.24)		

[1]Numbers in brackets are 95% confidence intervals (CIs) of the estimates. [2]The *p*-value is calculated from the estimated gamma and its 95% CI, assuming the estimates of Gamma are nearly normal distributed. [3]*P*-value for model comparison using the expected log pointwise posterior density. The second model is a better fit if *Z*-score is less than 0. MS: multiple sclerosis. IBD: inflammatory bowel disease. UC: ulcerative colitis. CD: Crohn’s disease. MHC: major histocompatibility complex. SNP: single nucleotide polymorphisms. CAUSE: Causal Analysis Using Summary Effect Estimates.

Table S8. Summary of enrichment correlations among MS, IBD, UC, and CD for GTEx tissues and cell types (by tissues)

Trait 1–Trait 2	MS–IBD	MS–UC	MS–CD	UC–CD
GTEx tissues (Bryois et al. 2020 method, No of tissues = 37)				
Correlation	0.84	0.80	0.81	0.85
p-value	1.20×10^{-10}	2.78×10^{-9}	1.68×10^{-9}	3.56×10^{-11}
GTEx tissues (Finucane et al. 2018 method, No of tissues = 45)				
Correlation	0.73	0.66	0.74	0.76
p-value	4.76×10^{-9}	7.84×10^{-7}	6.32×10^{-9}	1.80×10^{-9}
Cell types in lung (No of cell types = 28)				
Correlation	0.73	0.67	0.70	0.72
p-value	1.02×10^{-5}	8.53×10^{-5}	3.09×10^{-5}	1.88×10^{-5}
Cell types in PBMC (No of cell types = 11)				
Correlation	0.6	0.57	0.67	0.57
p-value	0.050	0.069	0.025	0.064
Cell types in spleen (No of cell types = 30)				
Correlation	0.26	0.17	0.22	0.80
p-value	0.17	0.37	0.24	1.36×10^{-7}
Cell types in small intestine (No of cell types = 15)				
Correlation	-0.10	0.22	-0.39	0.54
p-value	0.74	0.43	0.15	0.037

MS: multiple sclerosis. IBD: inflammatory bowel disease. UC: ulcerative colitis. CD: Crohn's disease. GTEx: Genotype-Tissue Expression. PBMC: peripheral blood mononuclear cells.

Table S9. Summary of significant SMR associations (SMR *p*-value <5.28×10^{-7}) involving novel SNPs associated with cross-trait MS and IBD (or UC or CD); shown in Table S3

Diseases	Cross-trait MS & CD	Cross-trait MS & CD	Cross-trait MS & IBD	Cross-trait MS & IBD	Cross-trait MS & CD
eQTL data	eQTLGen (Whole Blood)	eQTLGen (Whole Blood)	eQTLGen (Whole Blood)	eQTLGen (Whole Blood)	eQTLGen (Whole Blood)
Gene	*DNMT3A*	*POMC*	*DNMT3A*	*POMC*	*ETS2*
Chromosome	2	2	2	2	21
Probe position (hg19)	25510652	25387747	25510652	25387747	40187055
Top SNP	rs13428812	rs13428812	rs13428812	rs13428812	rs9977672
Top SNP position (hg19)	25492467	25492467	25492467	25492467	40463283
effect allele	G	G	G	G	A
non-effect allele	A	A	A	A	G
Beta (GWAS)	0.06	0.06	0.06	0.06	-0.06
P-value (GWAS)	2.67×10^{-11}	2.67×10^{-11}	6.84×10^{-11}	6.84×10^{-11}	6.32×10^{-11}
Beta (eQTL)	-0.12	-0.10	-0.12	-0.10	-0.15
P-value (eQTL)	2.98×10^{-34}	5.44×10^{-23}	2.98×10^{-34}	5.44×10^{-23}	5.47×10^{-42}
Beta (SMR)	-0.54	-0.67	-0.47	-0.58	0.44
P-value (SMR)	4.96×10^{-9}	3.33×10^{-8}	8.74×10^{-9}	5.23×10^{-8}	3.88×10^{-9}
P-value (HEIDI)	0.21	5.09×10^{-4}	0.31	7.56×10^{-4}	0.22
No of SNPs after HEIDI	20	10	20	10	9

*No significant SMR results identified using GTEx (Genotype-Tissue Expression) V7 tissue expression quantitative trait locus (eQTL) summary data. MS: multiple sclerosis. IBD: inflammatory bowel disease. CD: Crohn’s disease. SNP: single nucleotide polymorphisms. GWAS: Genome-wide Association Study. SMR: Summary-data-based Mendelian randomisation. HEIDI: HEterogeneity In Dependent Instrument.

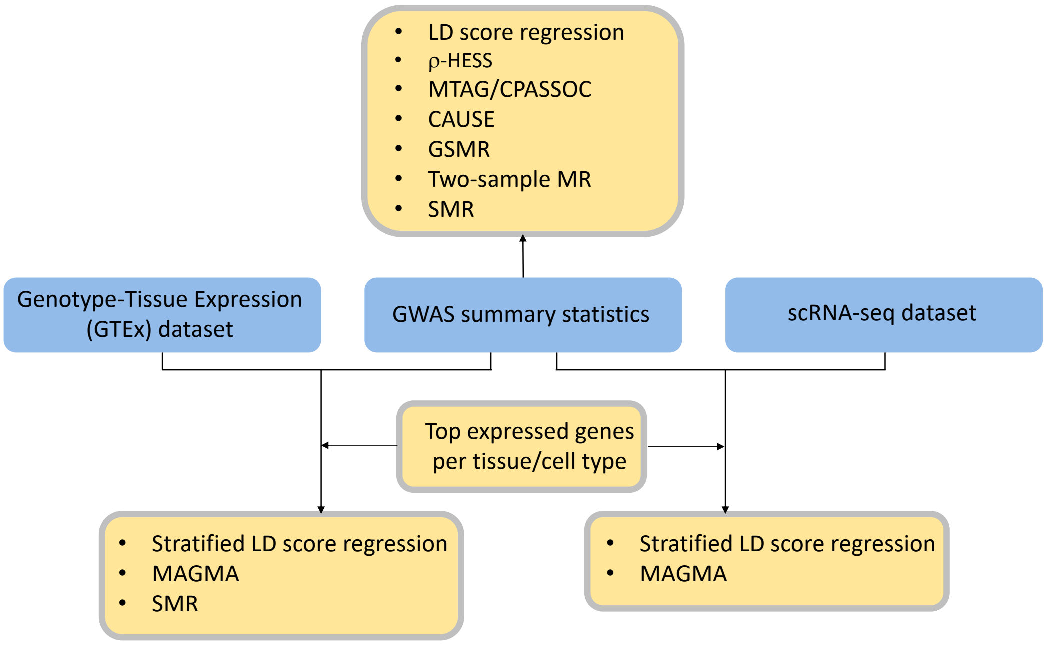

Figure S1. Overview of statistical analyses performed in the study. LD: linkage disequilibrium. ρ-HESS: Heritability Estimation from Summary Statistics. MTAG: Multi-Trait Analysis of GWAS. CPASSOC (Cross Phenotype Association). CAUSE: Causal Analysis Using Summary Effect Estimates. GSMR: Summary-data-based Mendelian Randomisation. MR: Mendelian Randomisation. GWAS: Genome-wide Association Study. scRNA-seq: single-cell RNA sequencing. MAGMA: Multi-marker Analysis of GenoMic Annotation. SMR: Summary-data-based Mendelian randomisation.

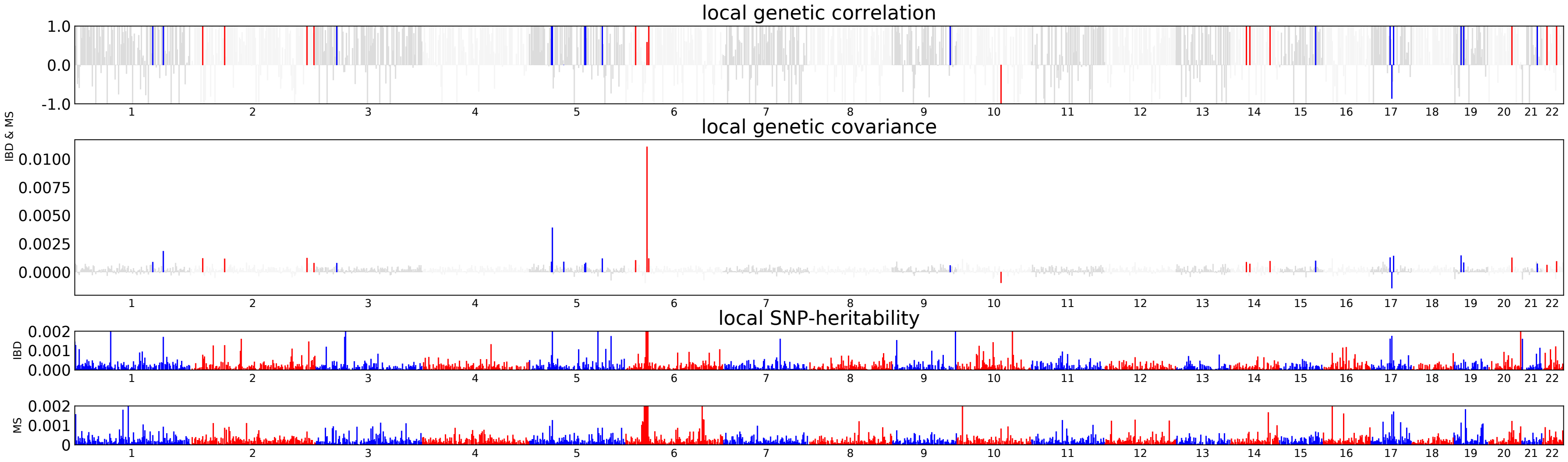

Figure S2. Local genetic correlations between multiple sclerosis (MS) and inflammatory bowel disease (IBD) revealed by ρ-HESS (Heritability Estimation from Summary Statistics). Significant local genetic correlation estimates are highlighted in red and blue for even and odd chromosomes, respectively.

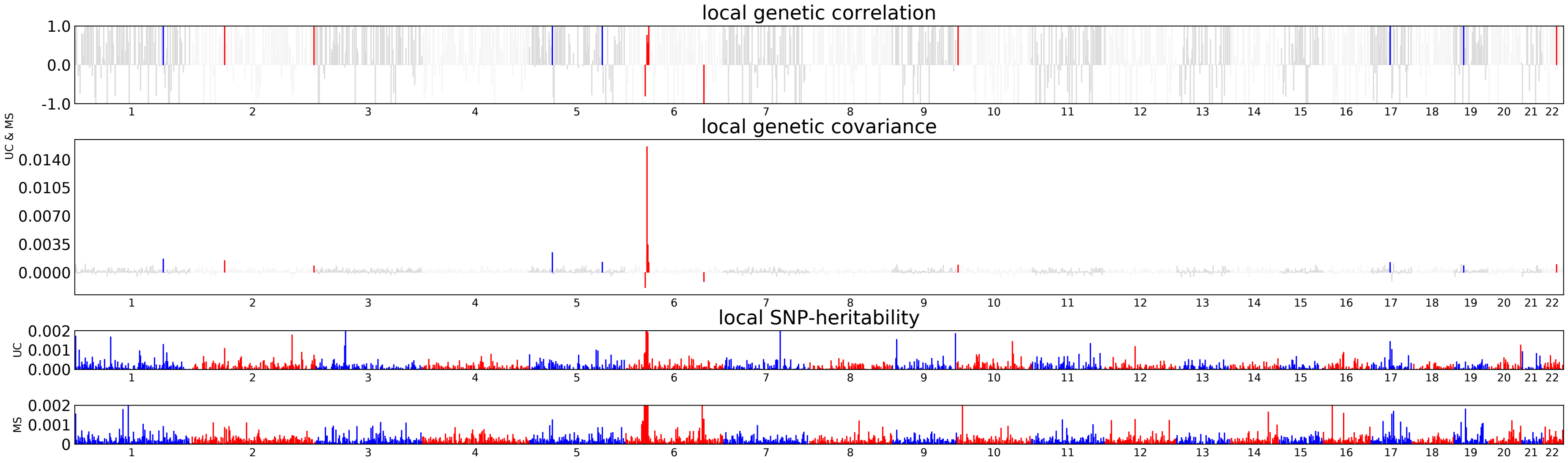

Figure S3. Local genetic correlations between multiple sclerosis (MS) and ulcerative colitis (UC) revealed by ρ-HESS (Heritability Estimation from Summary Statistics). Significant local genetic correlation estimates are highlighted in red and blue for even and odd chromosomes, respectively.

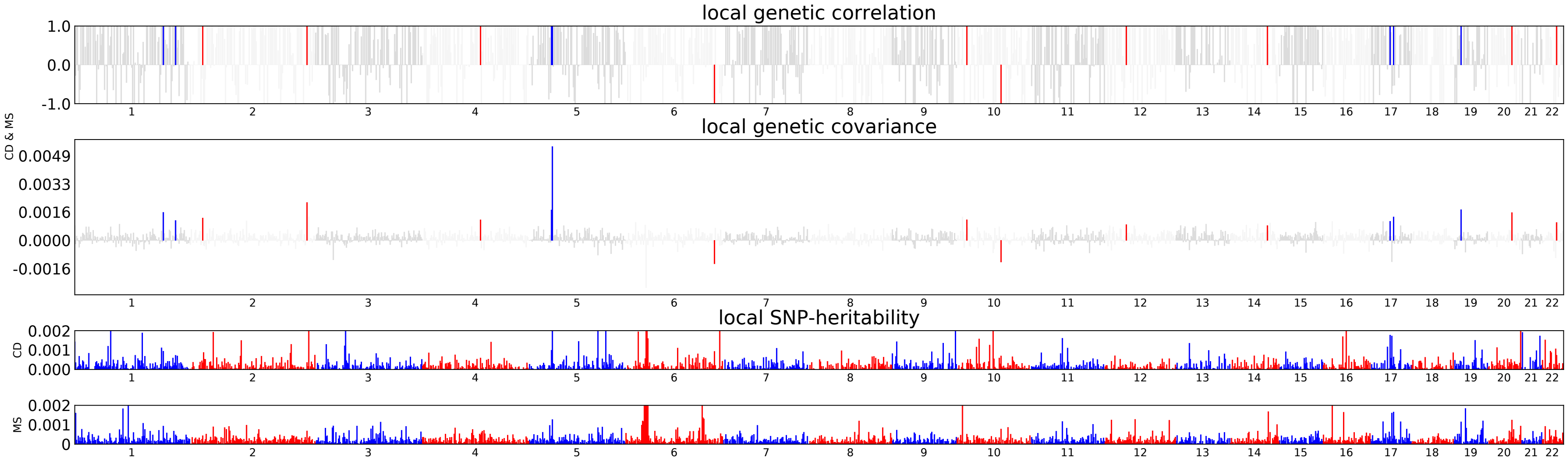

Figure S4. Local genetic correlations between multiple sclerosis (MS) and Crohn's disease (CD) revealed by ρ-HESS (Heritability Estimation from Summary Statistics). Significant local genetic correlation estimates are highlighted in red and blue for even and odd chromosomes, respectively.

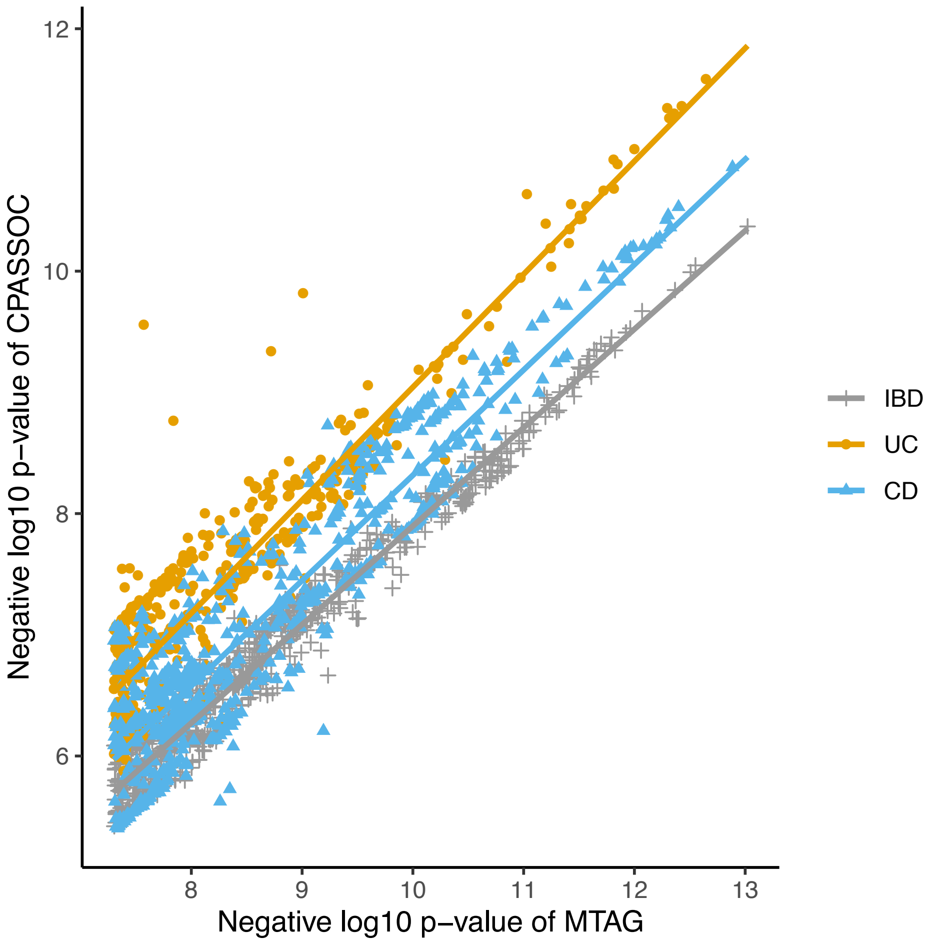

Figure S5. Comparison of negative log10 *p*-values for cross-trait genome-wide association study (GWAS) meta-analyses of multiple sclerosis (MS)-inflammatory bowel disease (IBD), MS-ulcerative colitis (UC) and MS-Crohn's disease (CD) performed using MTAG (Multi-Trait Analysis of GWAS) and CPASSOC (Cross Phenotype Association). Results are displayed for MTAG (x axis) and CPASSOC (y axis) for single nucleotide polymorphisms (SNPs) with genome-wide significant support ($p<5\times10^{-8}$) in the MTAG analyses, but not in the original single-trait GWAS. Grey: MS-IBD SNPs; correlation=0.98 (95% confidence interval [CI]=0.98-0.99; Spearman's test p-value$<1\times10^{-300}$). Orange: MS-UC SNPs; correlation=0.93 (95% CI=0.91-0.94; Spearman's test p-value$=1.27\times10^{-216}$). Blue: MS-CD SNPs; correlation=0.93 (95% CI=0.92-0.94; Spearman's test p-value$<1\times10^{-300}$).

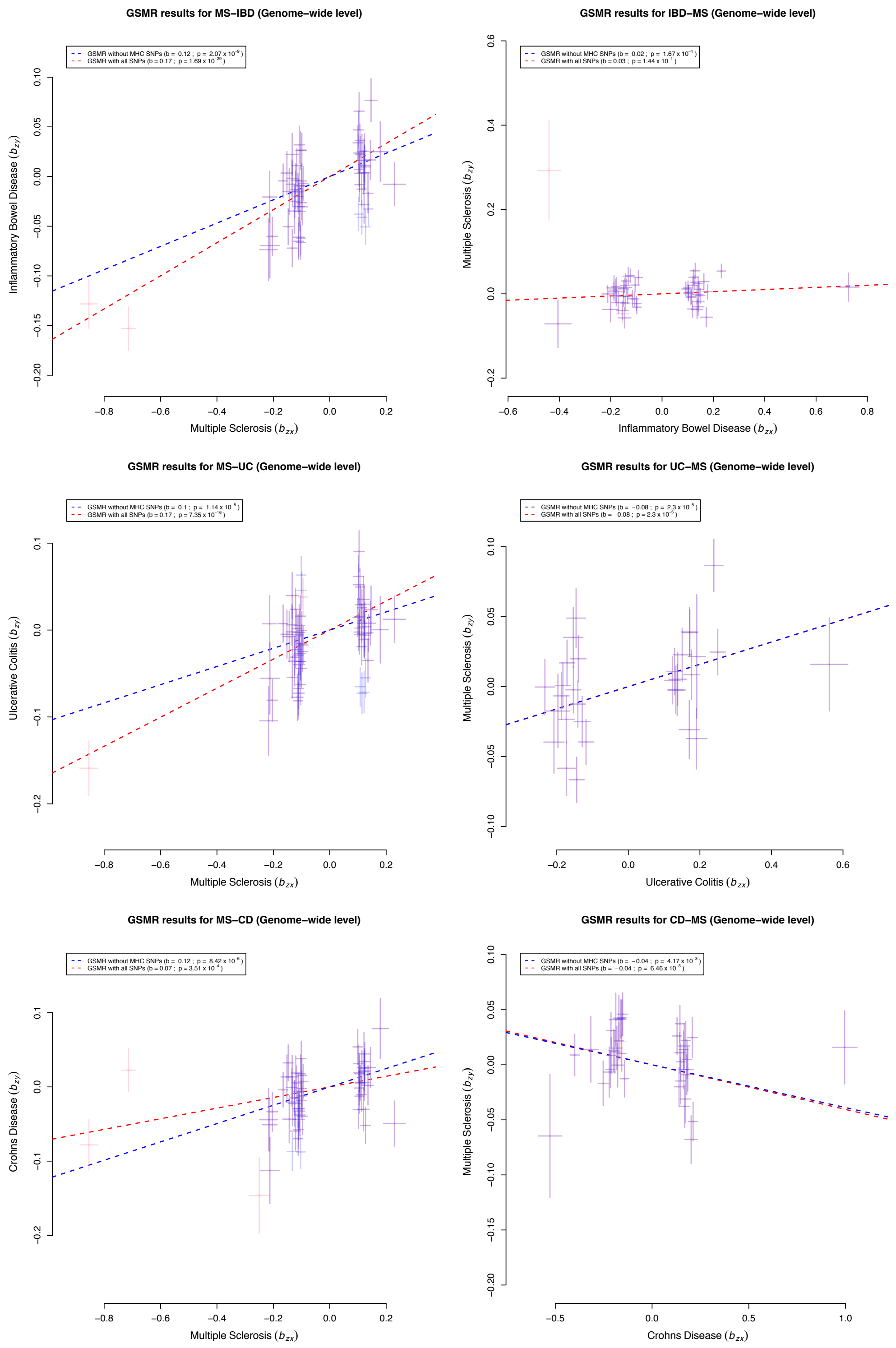

Figure S6. Generalised Summary-data-based Mendelian Randomisation (GSMR) effect size plots (with and without major histocompatibility complex [MHC] single nucleotide polymorphisms [SNPs]) for associations between multiple sclerosis (MS) and each of inflammatory bowel disease (IBD), ulcerative colitis (UC) and Crohn's disease (CD). For each plot, dots in pink represent MHC region SNPs; dots in purple represents non-MHC region SNPs; dots in blue represent SNPs included by HEIDI (HEterogeneity In Dependent Instrument) in GSMR analyses without MHC region SNPs, but removed by HEIDI for GSMR analyses including all SNPs.

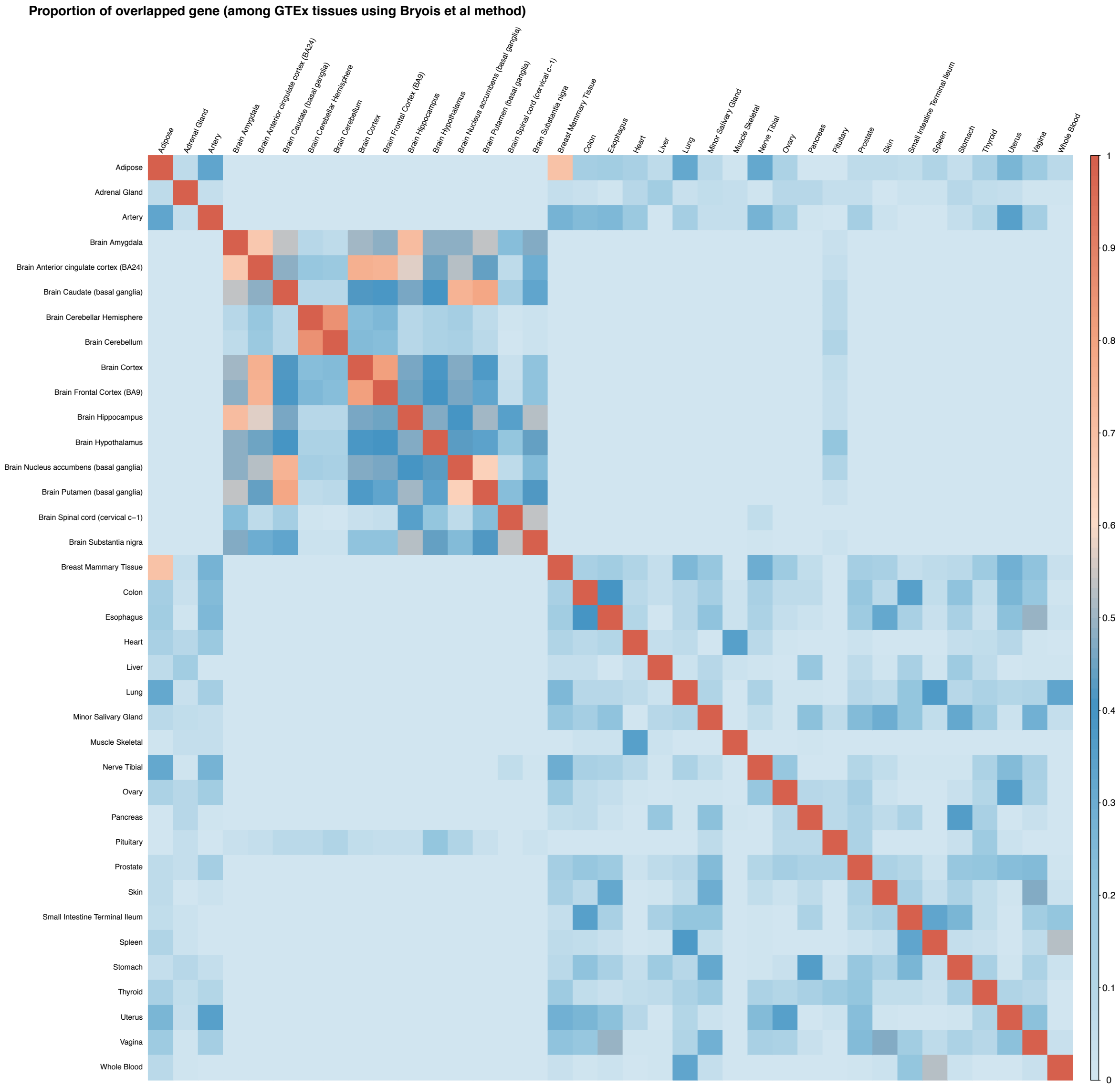

Figure S7. Proportion of overlap of the top 10% most specific genes among 37 GTEx (Genotype-Tissue Expression) tissues[4] using the Bryois et al. (2020) method.

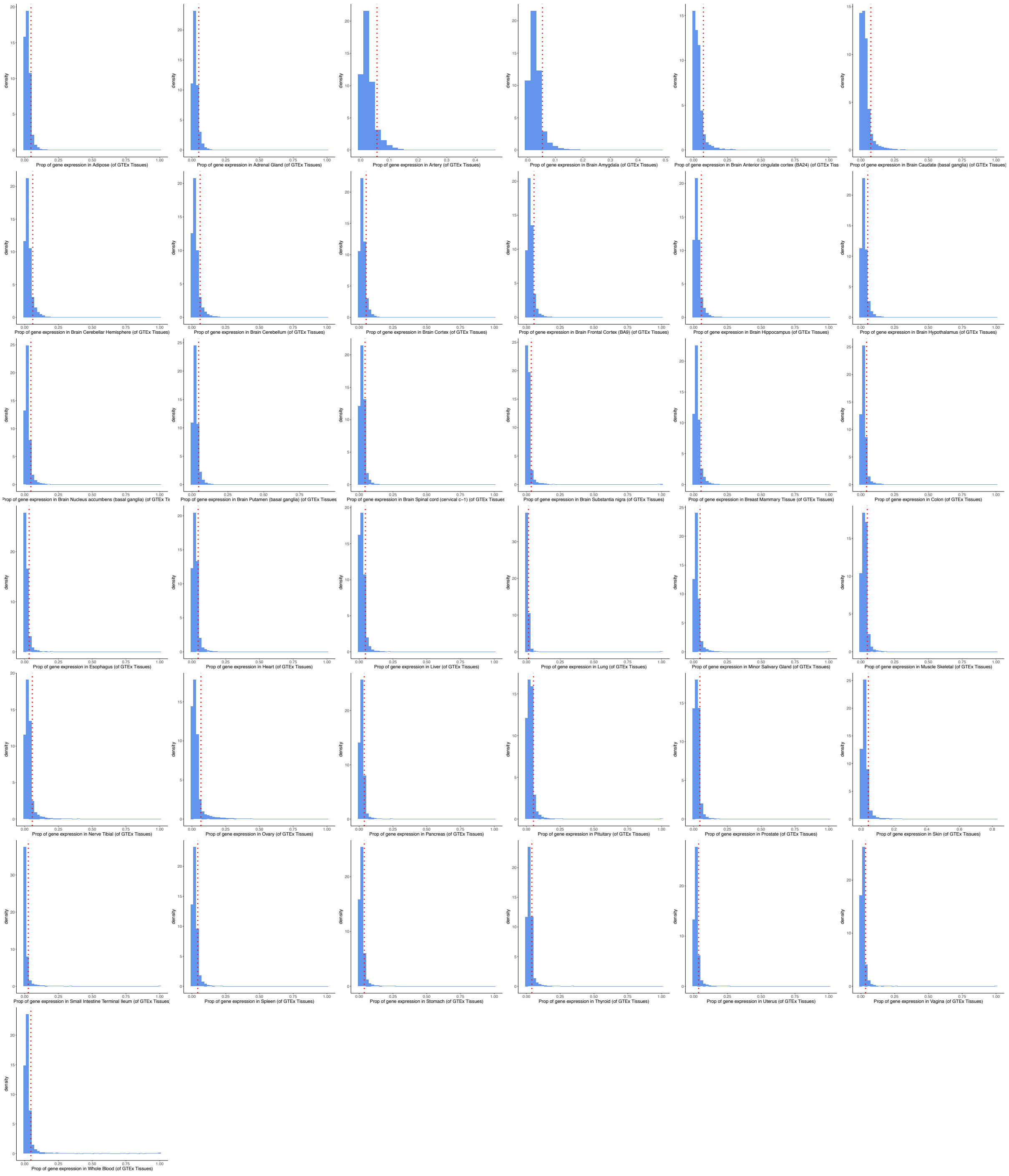

Figure S8. Distribution of the proportion of total expression per gene in each of 37 GTEx (Genotype-Tissue Expression) tissues[4] using the Bryois et al. (2020) method for defining tissue-specific genes. For each tissue, the top 10% most tissue-specific genes are distributed to the right of the red dotted vertical line.

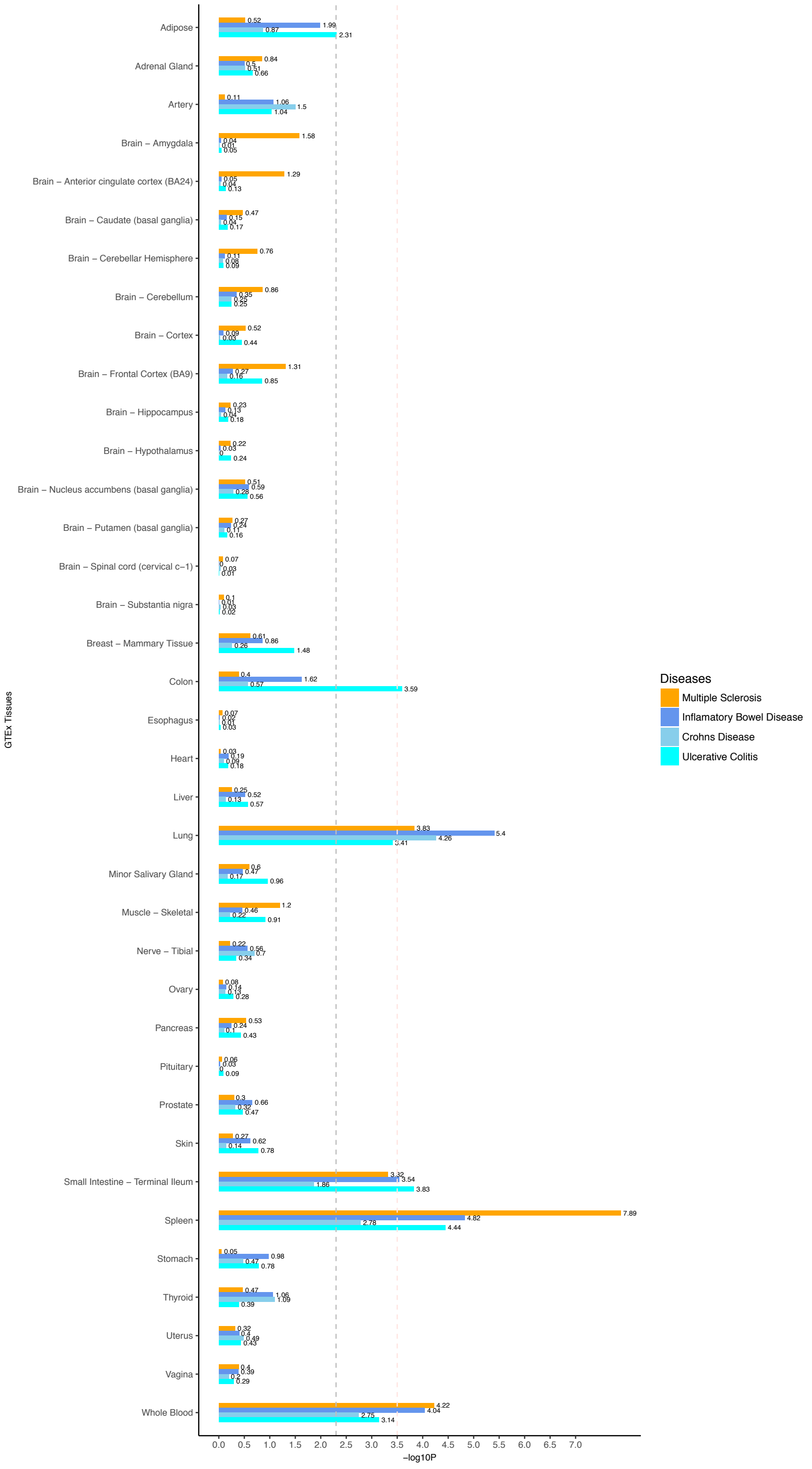

Figure S9. Stratified linkage disequilibrium score regression (S-LDSC)-based heritability enrichment estimates in 37 GTEx (Genotype-Tissue Expression) tissues[4] for multiple sclerosis (MS) and each of inflammatory bowel disease (IBD), ulcerative colitis (UC) and Crohn's disease (CD), using the Bryois et al. (2020) method for defining tissue-specific genes. Negative log10 *p*-values of coefficient *Z*-scores for each individual test (two-tailed *Z*-test) are displayed on the x axis. The grey and pink dotted lines represent the false discovery rate (FDR) <5% and Bonferroni corrected thresholds for multiple regressions, respectively.

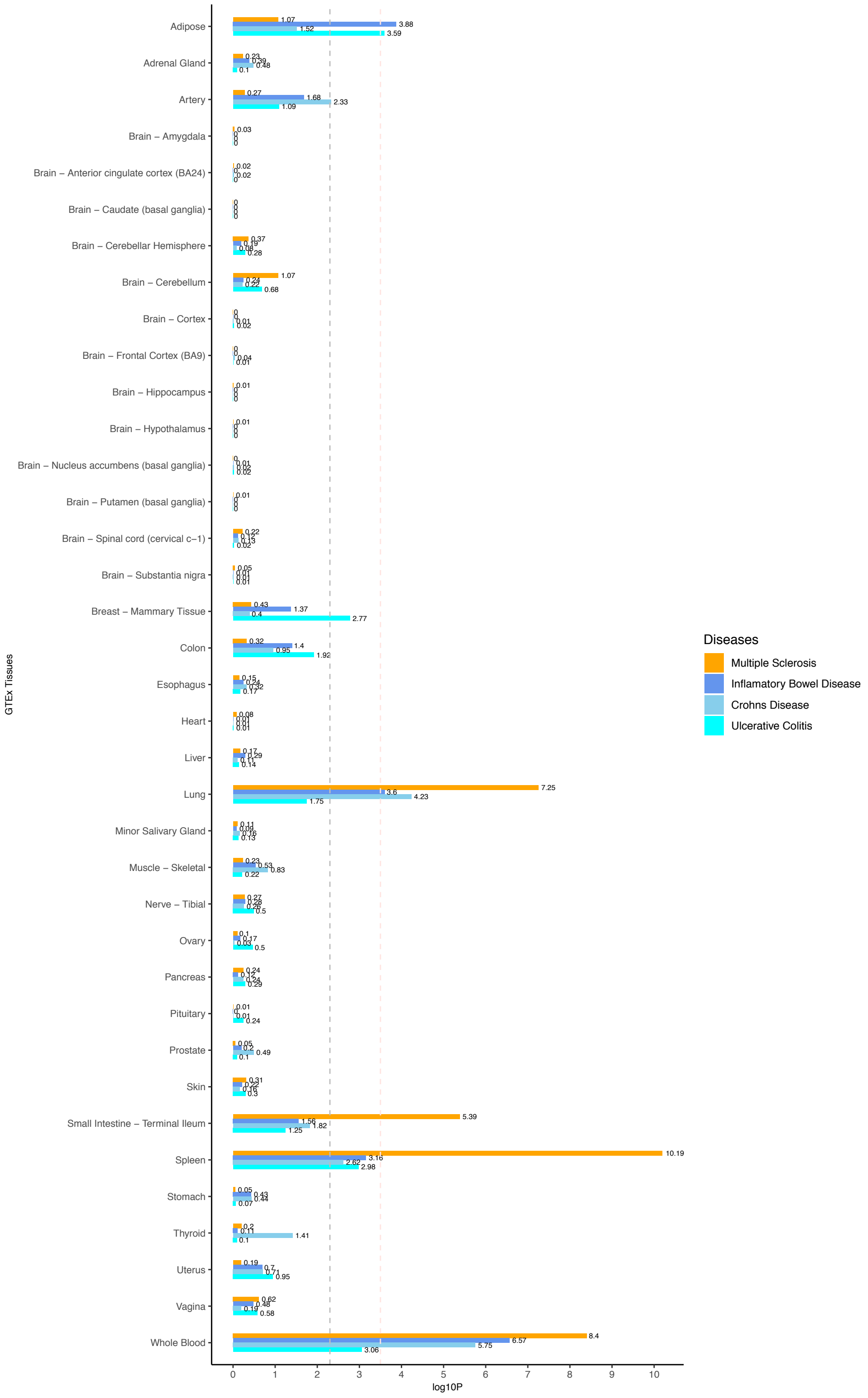

Figure S10. MAGMA (Multi-marker Analysis of GenoMic)-based heritability enrichment estimates in 37 GTEx (Genotype-Tissue Expression) tissues[4] for multiple sclerosis (MS) and each of inflammatory bowel disease (IBD), ulcerative colitis (UC) and Crohn's disease (CD), without genes in the major histocompatibility complex (MHC) region. Negative log10 *p*-values of coefficient *Z*-scores for each individual test (two-tailed *Z*-test) are displayed on the x axis. The grey and pink dotted lines represent the false discovery rate (FDR) <5% and Bonferroni corrected thresholds for multiple regressions, respectively.

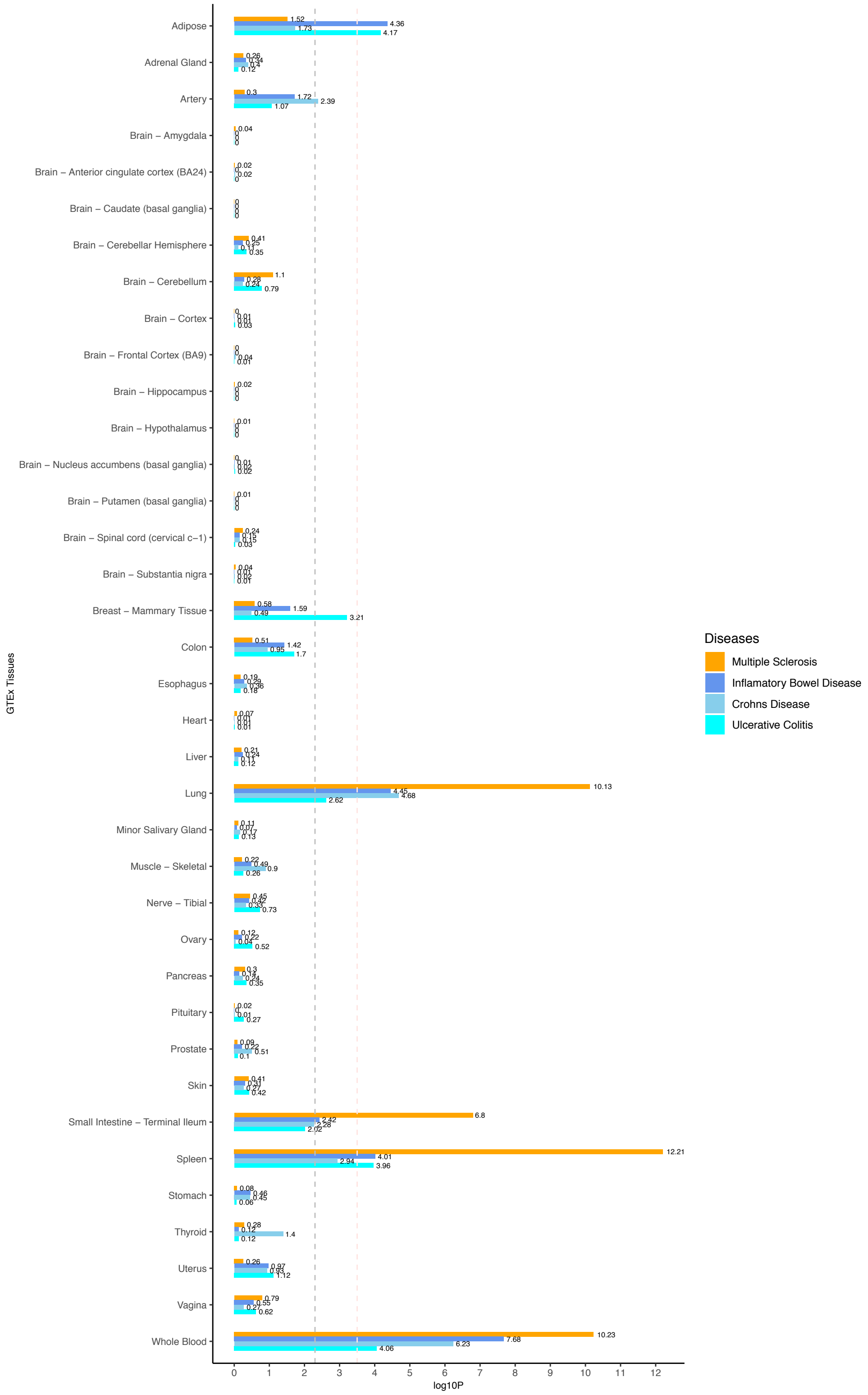

Figure S11. MAGMA (Multi-marker Analysis of GenoMic)-based heritability enrichment estimates in 37 GTEx (Genotype-Tissue Expression) tissues[4] for multiple sclerosis (MS) and each of inflammatory bowel disease (IBD), ulcerative colitis (UC) and Crohn's disease (CD), including genes in the major histocompatibility complex (MHC) region. Negative log10 *p*-values of coefficient *Z*-scores for each individual test (two-tailed *Z*-test) are displayed on the x axis. The grey and pink dotted line represent the false discovery rate (FDR) <5% and Bonferroni corrected thresholds for multiple regressions, respectively.

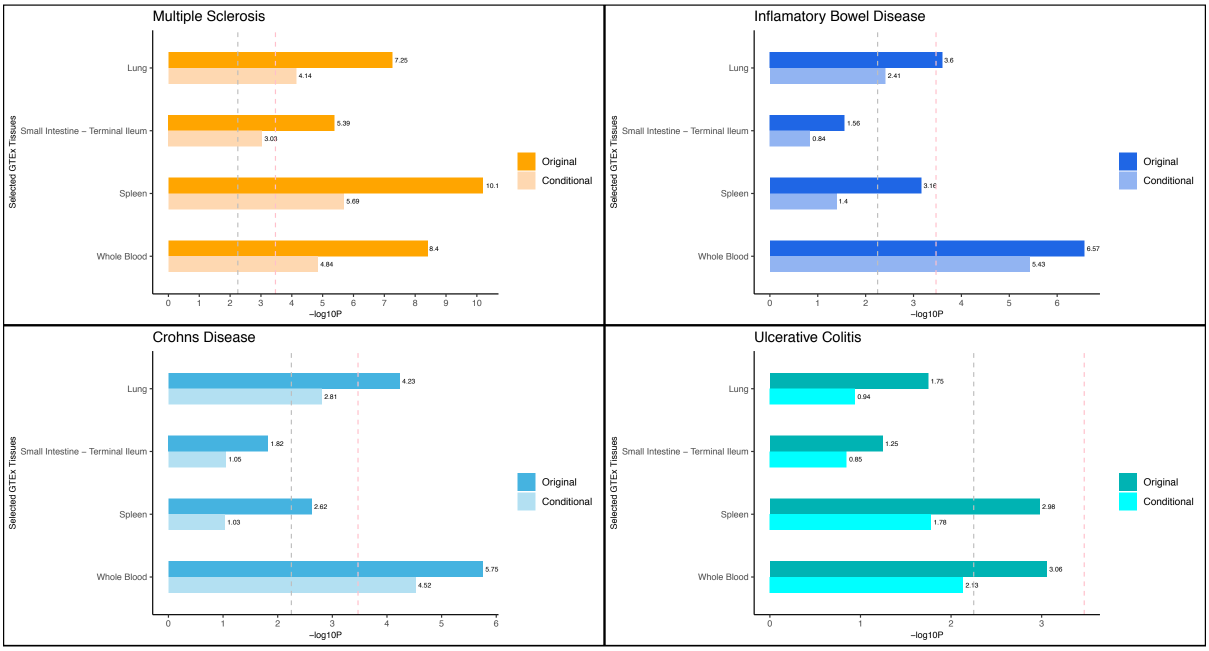

Figure S12. Tissue-specific enrichment of heritability in multiple sclerosis (MS), inflammatory bowel disease (IBD), ulcerative colitis (UC) and Crohn's disease (CD) in immune tissues using MAGMA (Multi-marker Analysis of GenoMic Annotation, without genes in the major histocompatibility complex [MHC] region). Negative log10 *p*-values of coefficient *Z*-scores for each individual test (two-tailed *Z*-test) are displayed on the x axis. The grey and pink dotted lines represent the false discovery rate (FDR) <5% and Bonferroni corrected thresholds for multiple regressions, respectively. Original indicates results from analyses adjusted for the baseline model and the set of all genes. Conditional indicates results from conditional analyses that additionally adjusted for the set of genes specifically expressed in the three non-focal tissues (e.g. small intestine - terminal ileum, lung and whole blood in analyses of spleen).

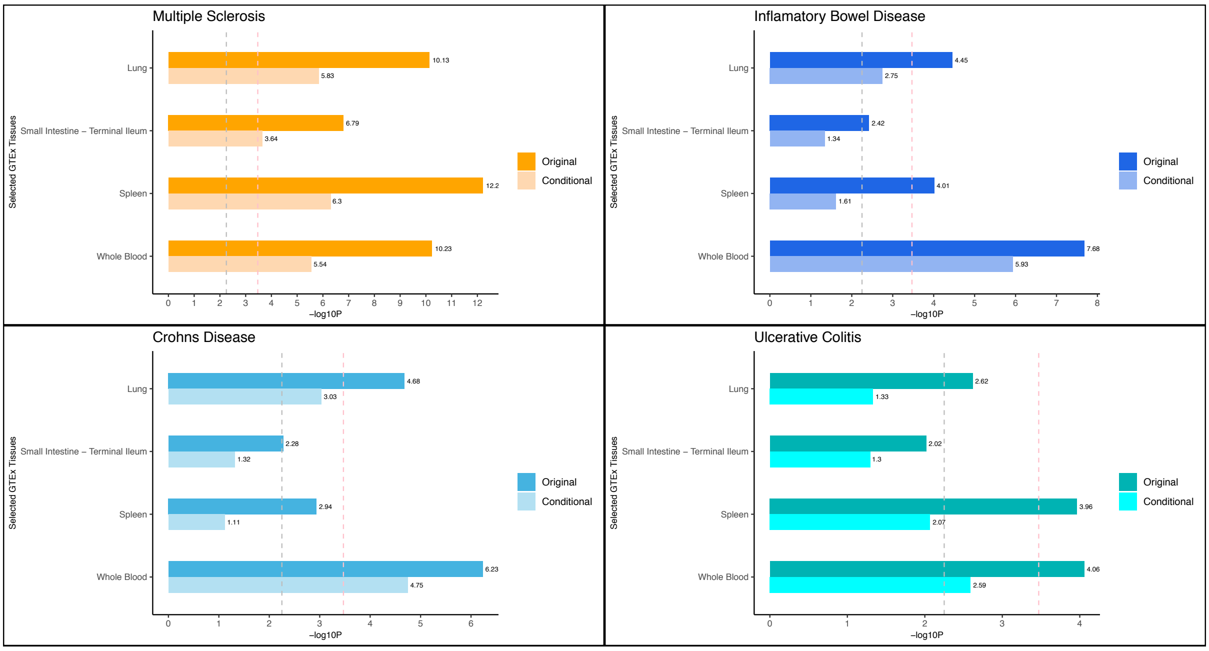

Figure S13. Tissue-specific enrichment of heritability in multiple sclerosis (MS), inflammatory bowel disease (IBD), ulcerative colitis (UC) and Crohn's disease (CD) in immune tissues using MAGMA (Multi-marker Analysis of GenoMic Annotation, including genes in the major histocompatibility complex [MHC] region). Negative log10 *p*-values of coefficient *Z*-scores for each individual test (two-tailed *Z*-test) are displayed on the x axis. The grey and pink dotted lines represent the false discovery rate (FDR) <5% and Bonferroni corrected thresholds for multiple regressions, respectively. Original indicates results from analyses adjusted for the baseline model and the set of all genes. Conditional indicates results from conditional analyses that additionally adjusted for the set of genes specifically expressed in the three non-focal tissues (e.g. small intestine - terminal ileum, lung and whole blood in analyses of spleen).

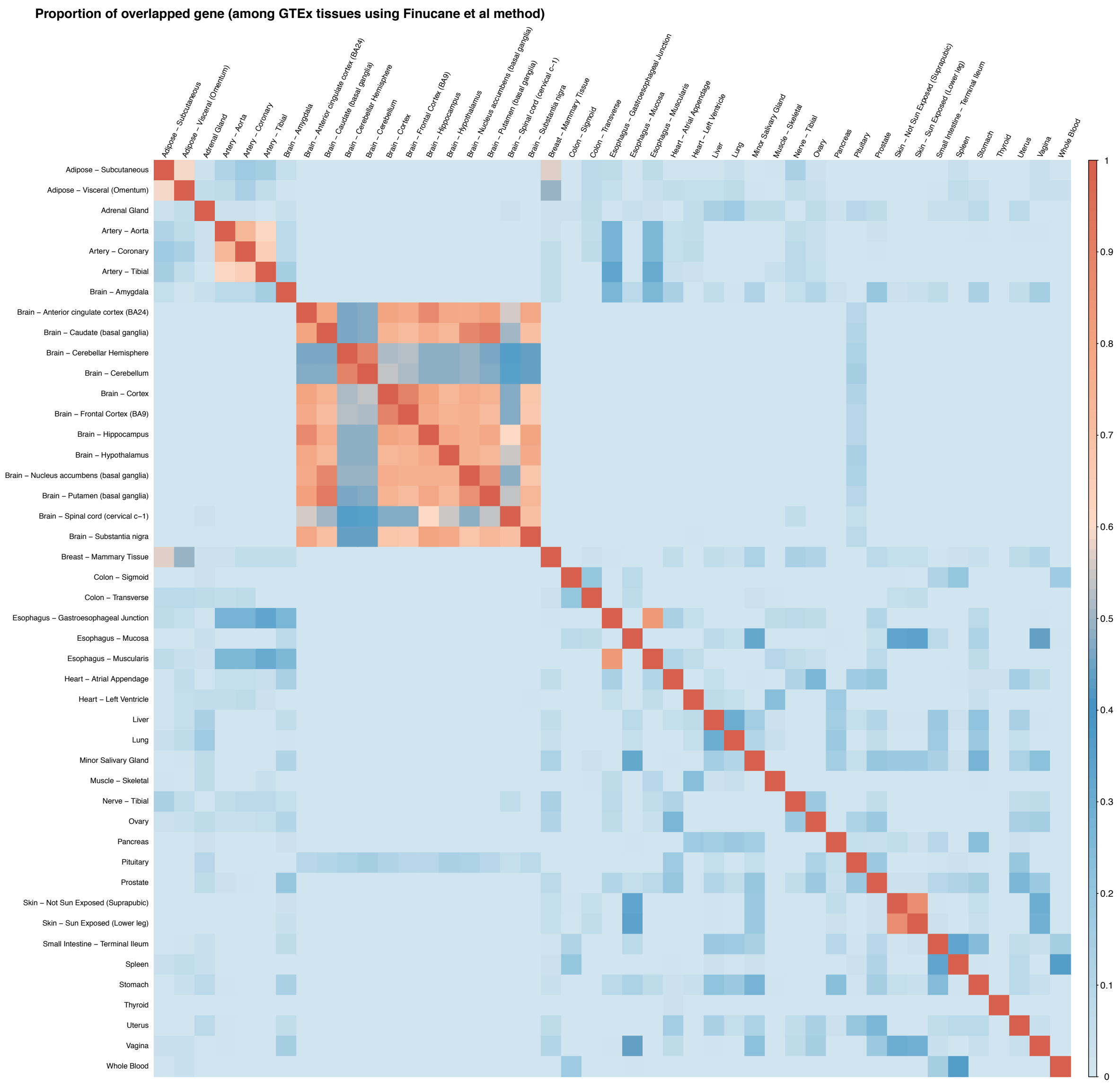

Figure S14. Proportion of overlap of the top 10% highly expressed genes among 45 GTEx (Genotype-Tissue Expression) tissues[4] using the Finucane et al. (2018) method.

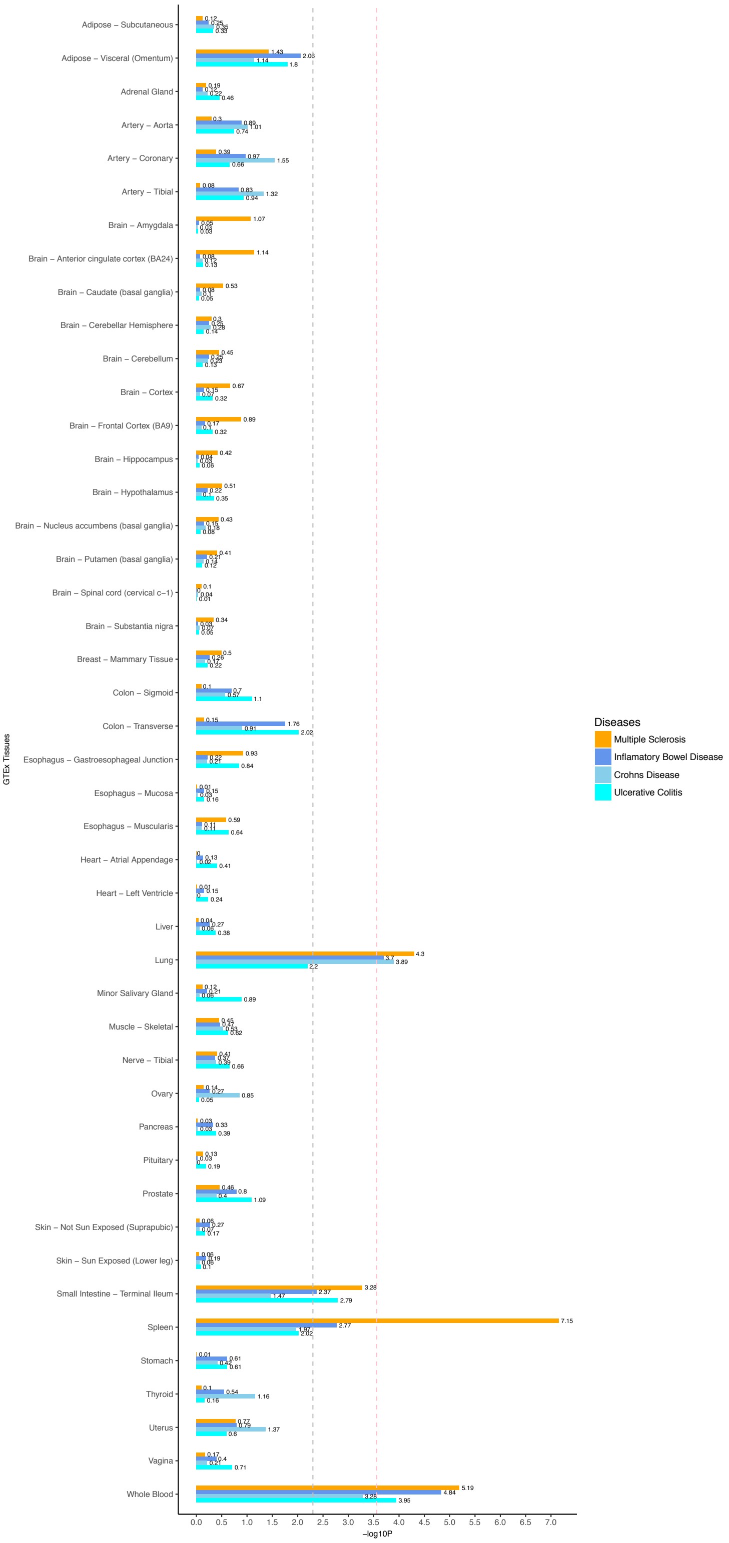

Figure S15. Stratified linkage disequilibrium score regression (S-LDSC)-based heritability enrichment estimates in 45 GTEx (Genotype-Tissue Expression) tissues[4] for multiple sclerosis (MS) and each of inflammatory bowel disease (IBD), ulcerative colitis (UC) and Crohn's disease (CD), using the Finucane et al. (2018) method. Negative log10 *p*-values of coefficient *Z*-scores for each individual test (two-tailed *Z*-test) are displayed on the x axis. The grey and pink dotted lines represent the false discovery rate (FDR) <5% and Bonferroni corrected thresholds for multiple regressions, respectively.

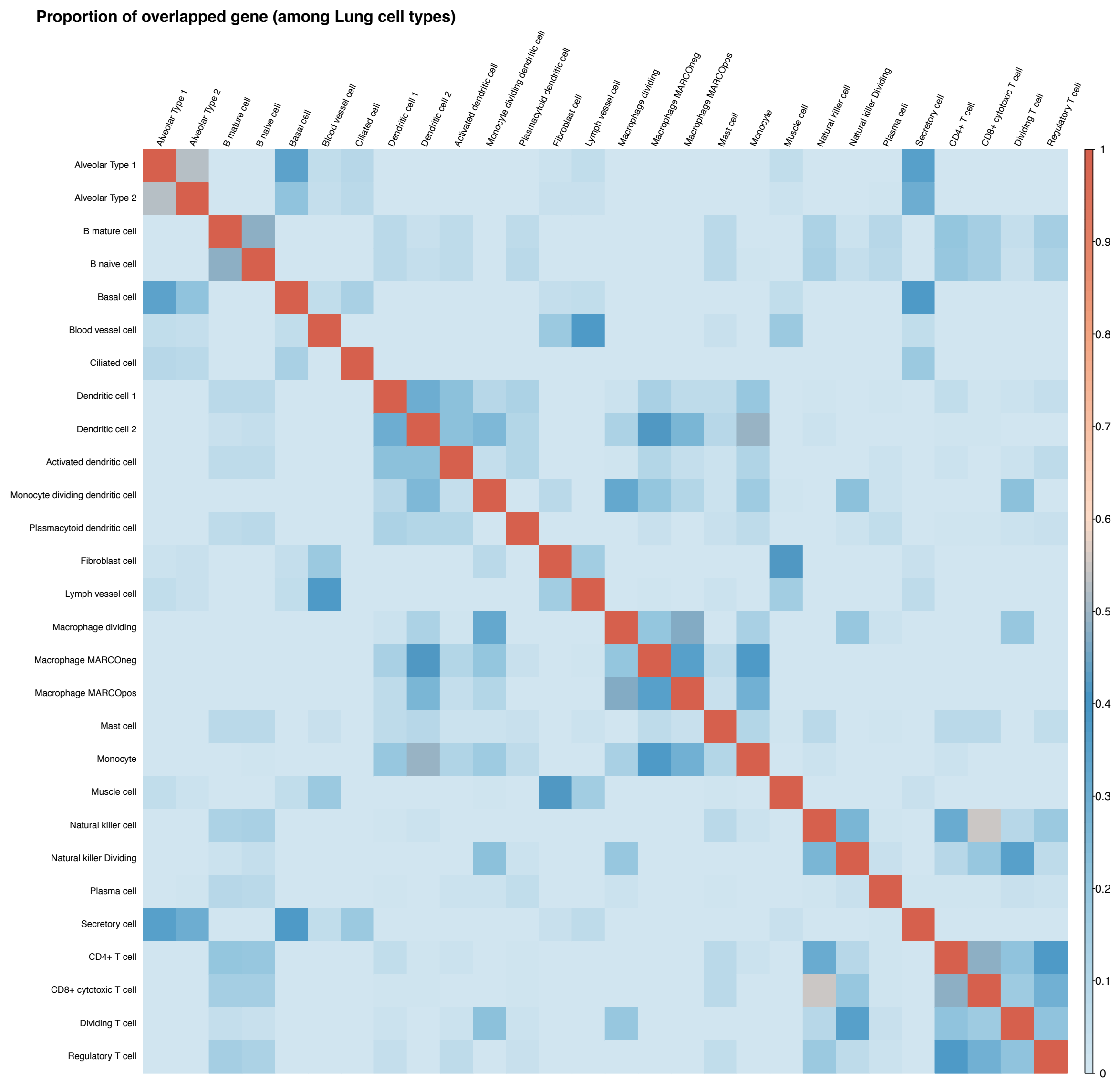

Figure S16. Proportion of overlap of the top 10% most specific genes among 28 lung cell types[5], using the Bryois et al. (2020) method.

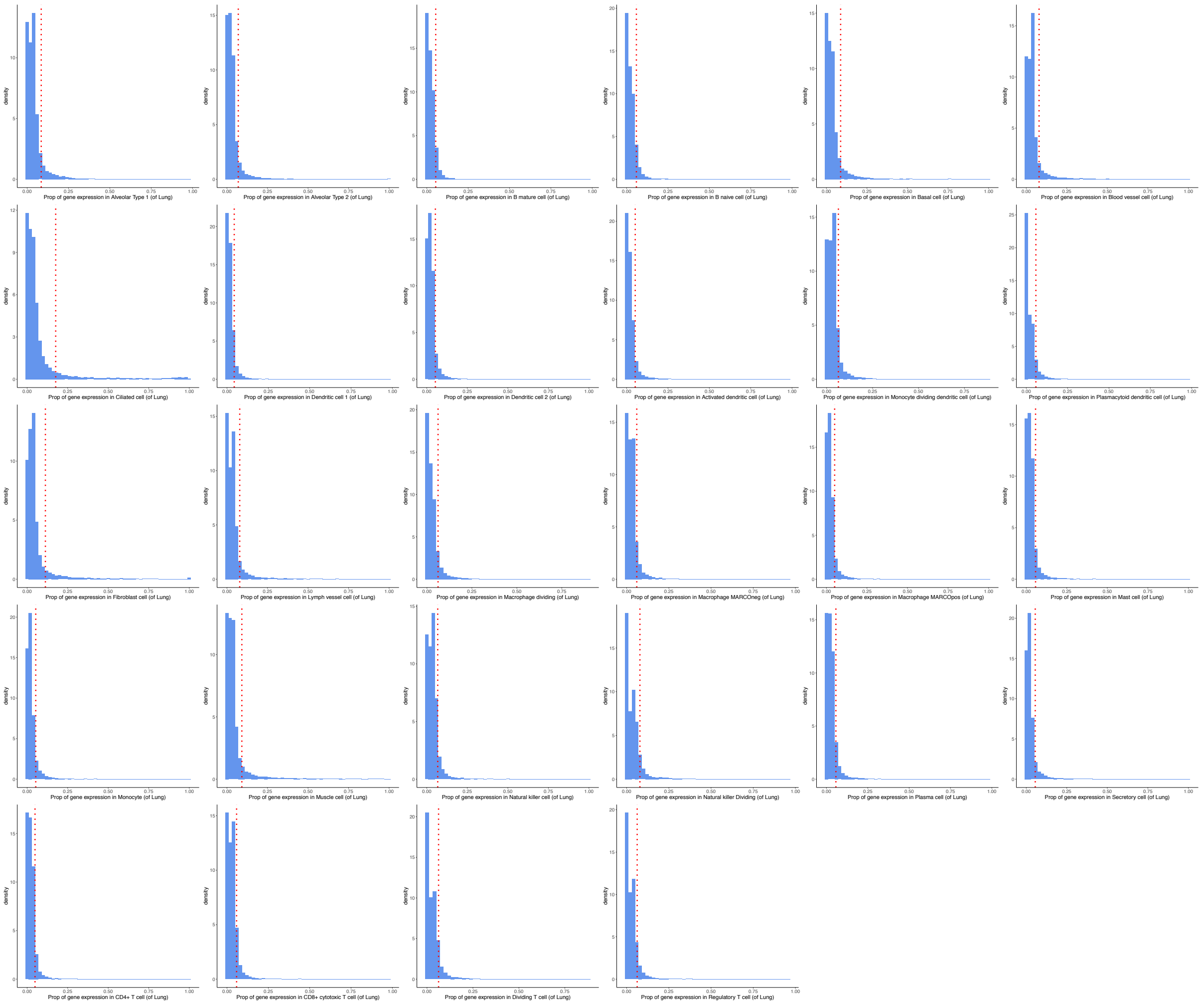

Figure S17. Distribution of the proportion of total expression per gene in each of the 28 lung cell types[5], using the Bryois et al. (2020) method for defining cell type-specific genes. For each cell type, the top 10% most cell type-specific genes are distributed in the right of the red dotted vertical line.

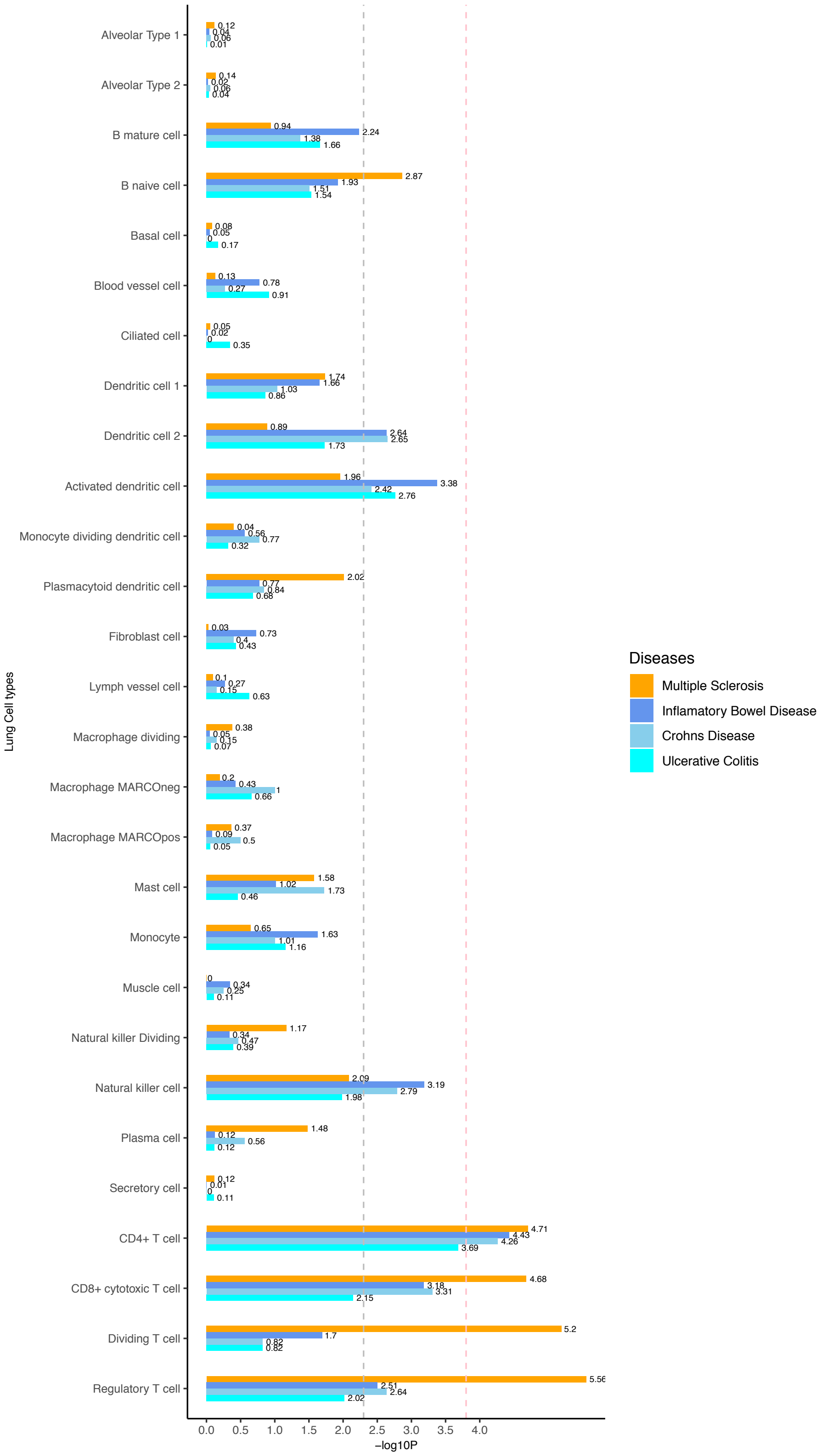

Figure S18. Stratified linkage disequilibrium score regression (S-LDSC)-based heritability enrichment estimates in 28 lung cell types[5] for multiple sclerosis (MS) and each of inflammatory bowel disease (IBD), ulcerative colitis (UC) and Crohn's disease (CD). Negative log10 *p*-values of coefficient *Z*-scores for each individual test (two-tailed *Z*-test) are displayed on the x axis. The grey and pink dotted lines represent the false discovery rate (FDR) <5% and Bonferroni corrected thresholds for multiple regressions, respectively.

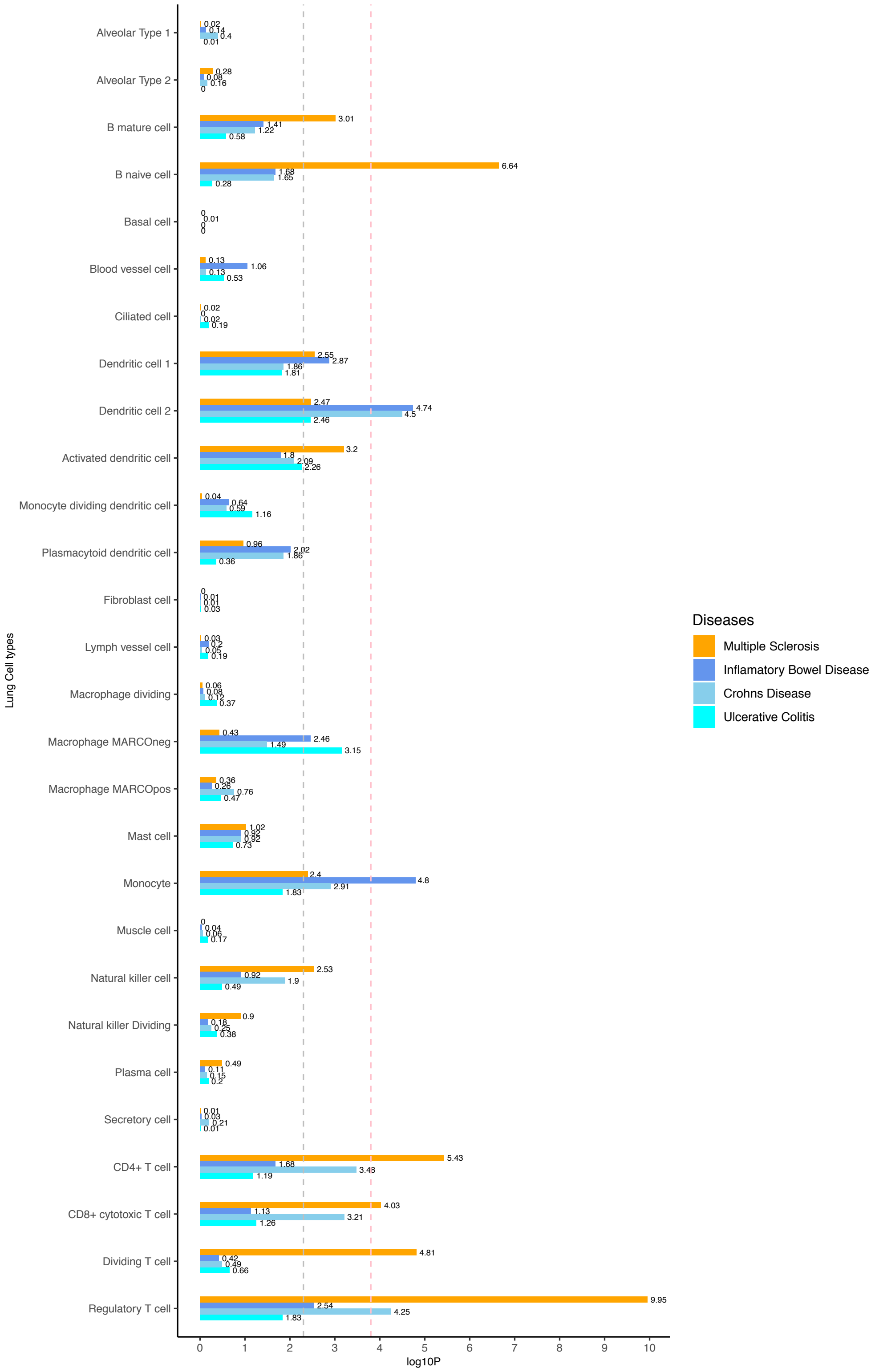

Figure S19. MAGMA (Multi-marker Analysis of GenoMic)-based heritability enrichment in 28 lung cell types[5] for multiple sclerosis (MS) and each of inflammatory bowel disease (IBD), ulcerative colitis (UC) and Crohn's disease (CD), without genes in the major histocompatibility complex (MHC) region. Negative log10 *p*-values of coefficient *Z*-scores for each individual test (two-tailed *Z*-test) are displayed on the x axis. The grey and pink dotted lines represent the false discovery rate (FDR) <5% and Bonferroni corrected thresholds for multiple regressions, respectively.

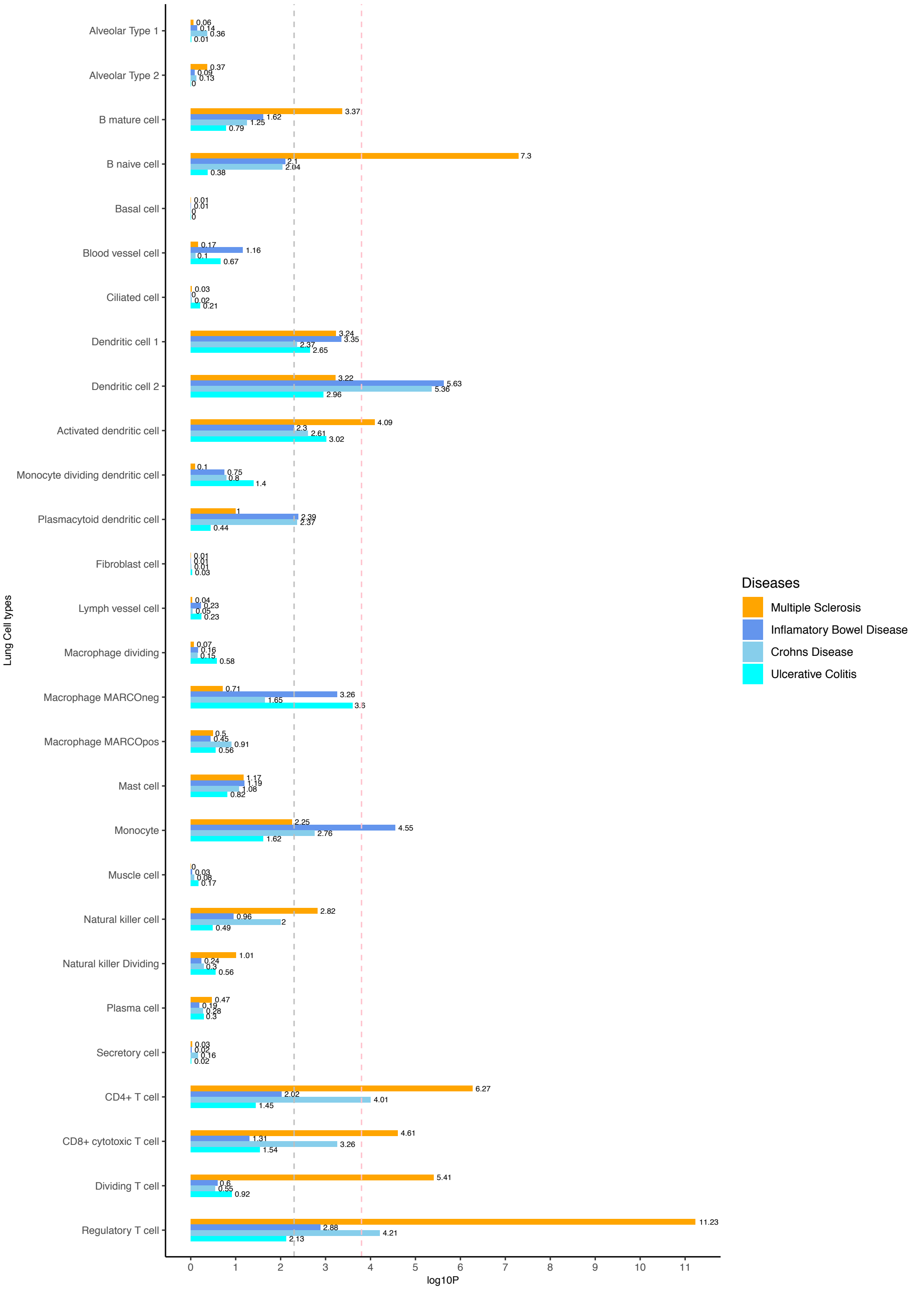

Figure S20. MAGMA (Multi-marker Analysis of GenoMic)-based heritability enrichment estimates in 28 lung cell types[5] for multiple sclerosis (MS) and each of inflammatory bowel disease (IBD), ulcerative colitis (UC) and Crohn's disease (CD), including genes in the major histocompatibility complex (MHC) region. Negative log10 *p*-values of coefficient *Z*-scores for each individual test (two-tailed *Z*-test) are displayed on the x axis. The grey and pink dotted lines represent the false discovery rate (FDR) <5% and Bonferroni corrected thresholds for multiple regressions, respectively.

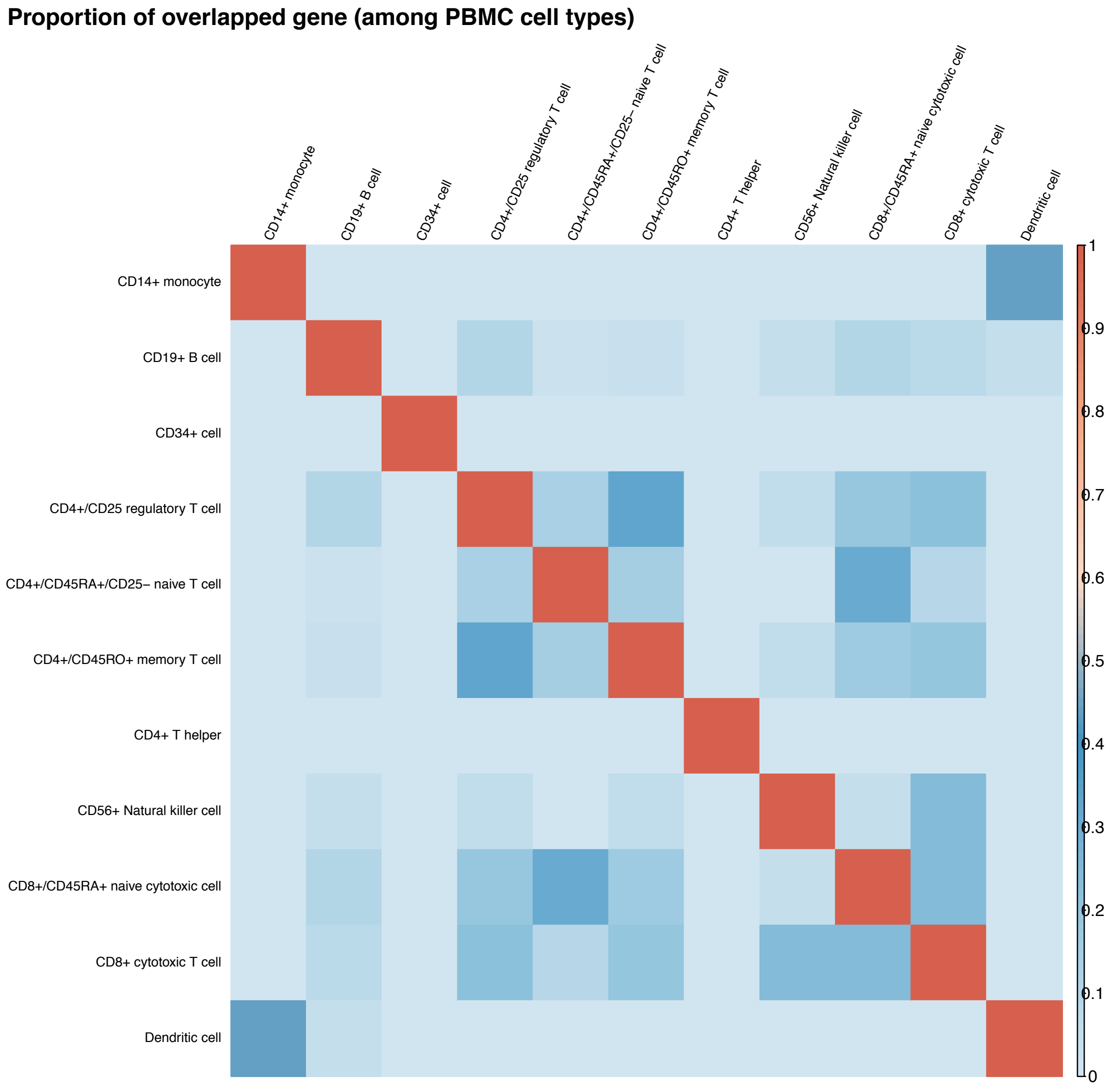

Figure S21. Proportion of overlap of the top 10% most specific genes among 11 peripheral blood mononuclear cells (PBMC) cell types[6], using the Bryois et al. (2020) method.

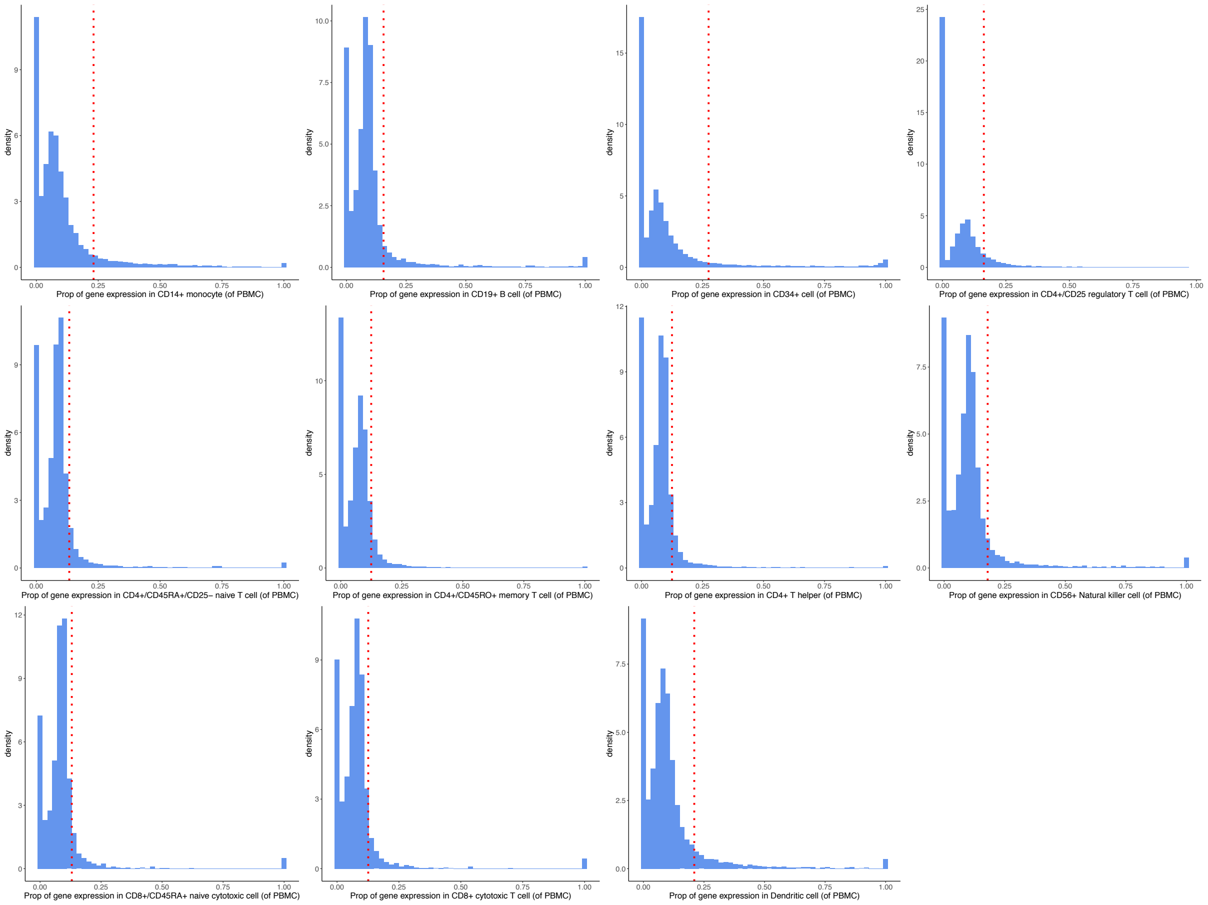

Figure S22. Distribution of the proportion of total expression per gene in each of the 11 peripheral blood mononuclear cells (PBMC) cell types[6], using the Bryois et al. (2020) method for defining cell type-specific genes. For each cell type, the top 10% most cell type-specific genes are distributed in the right of the red dotted vertical line.

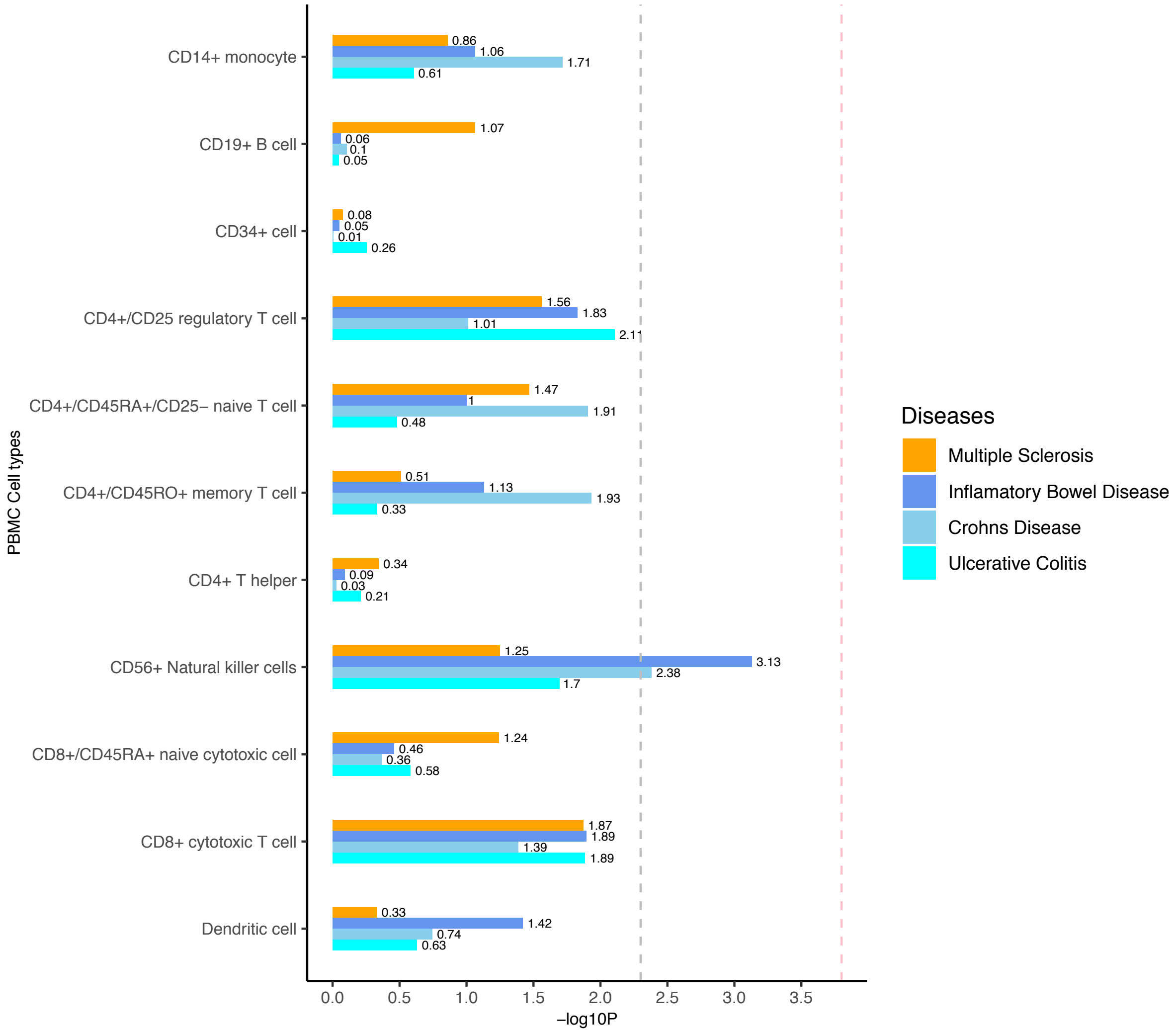

Figure S23. Stratified linkage disequilibrium score regression (S-LDSC)-based heritability enrichment estimates in 11 peripheral blood mononuclear cells (PBMC) cell types[6] for multiple sclerosis (MS) and each of inflammatory bowel disease (IBD), ulcerative colitis (UC) and Crohn's disease (CD). Negative log10 *p*-values of coefficient *Z*-scores for each individual test (two-tailed *Z*-test) are displayed on the x axis. The grey and pink dotted lines represent the false discovery rate (FDR) <5% and Bonferroni corrected thresholds for multiple regressions, respectively.

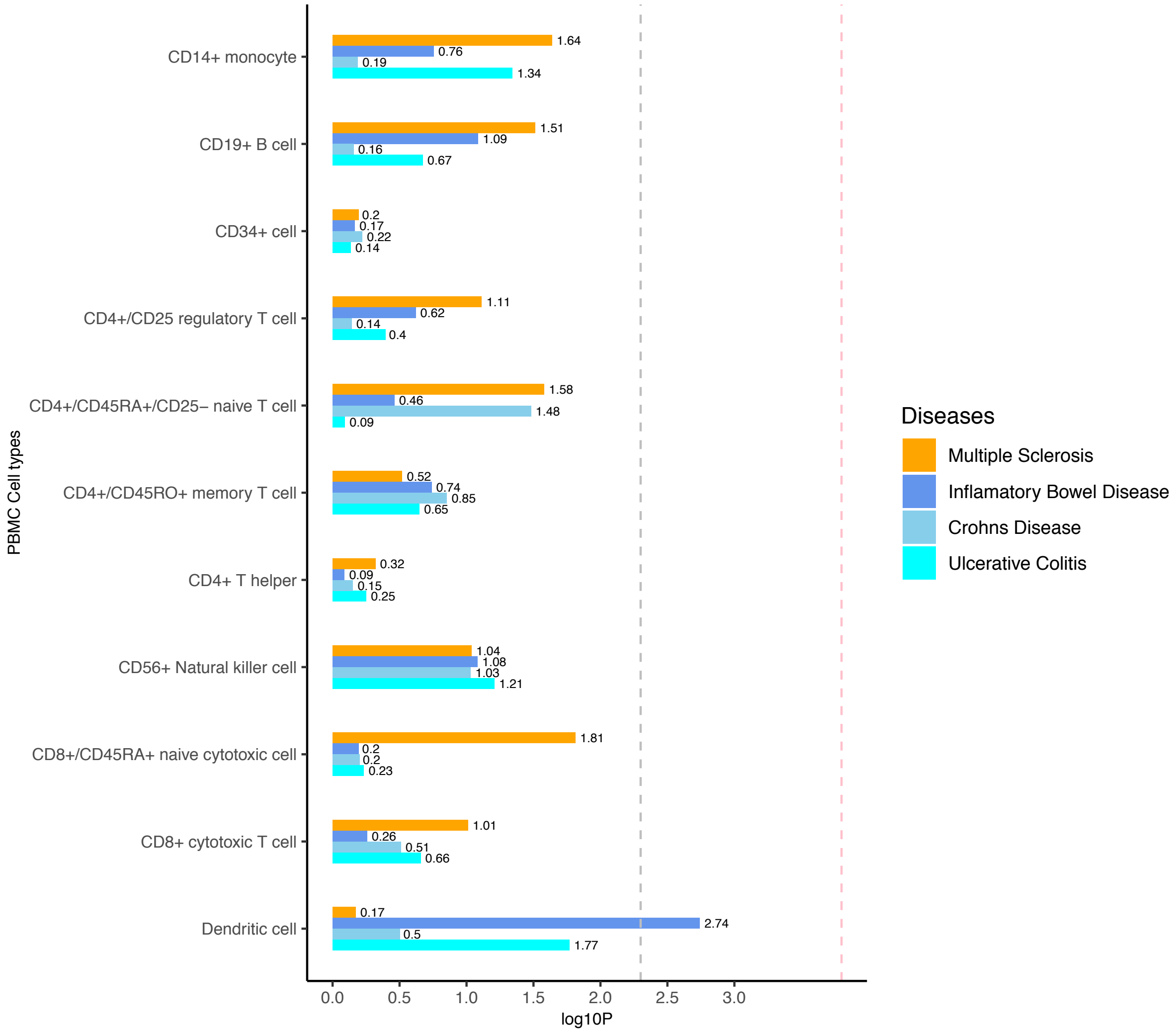

Figure S24. MAGMA (Multi-marker Analysis of GenoMic)-based heritability enrichment in 11 peripheral blood mononuclear cells (PBMC) cell types[6] for multiple sclerosis (MS) and each of inflammatory bowel disease (IBD), ulcerative colitis (UC) and Crohn's disease (CD), without genes in the major histocompatibility complex (MHC) region. Negative log10 *p*-values of coefficient *Z*-scores for each individual test (two-tailed *Z*-test) are displayed on the x axis. The grey and pink dotted lines represent the false discovery rate (FDR) <5% and Bonferroni corrected thresholds for multiple regressions, respectively.

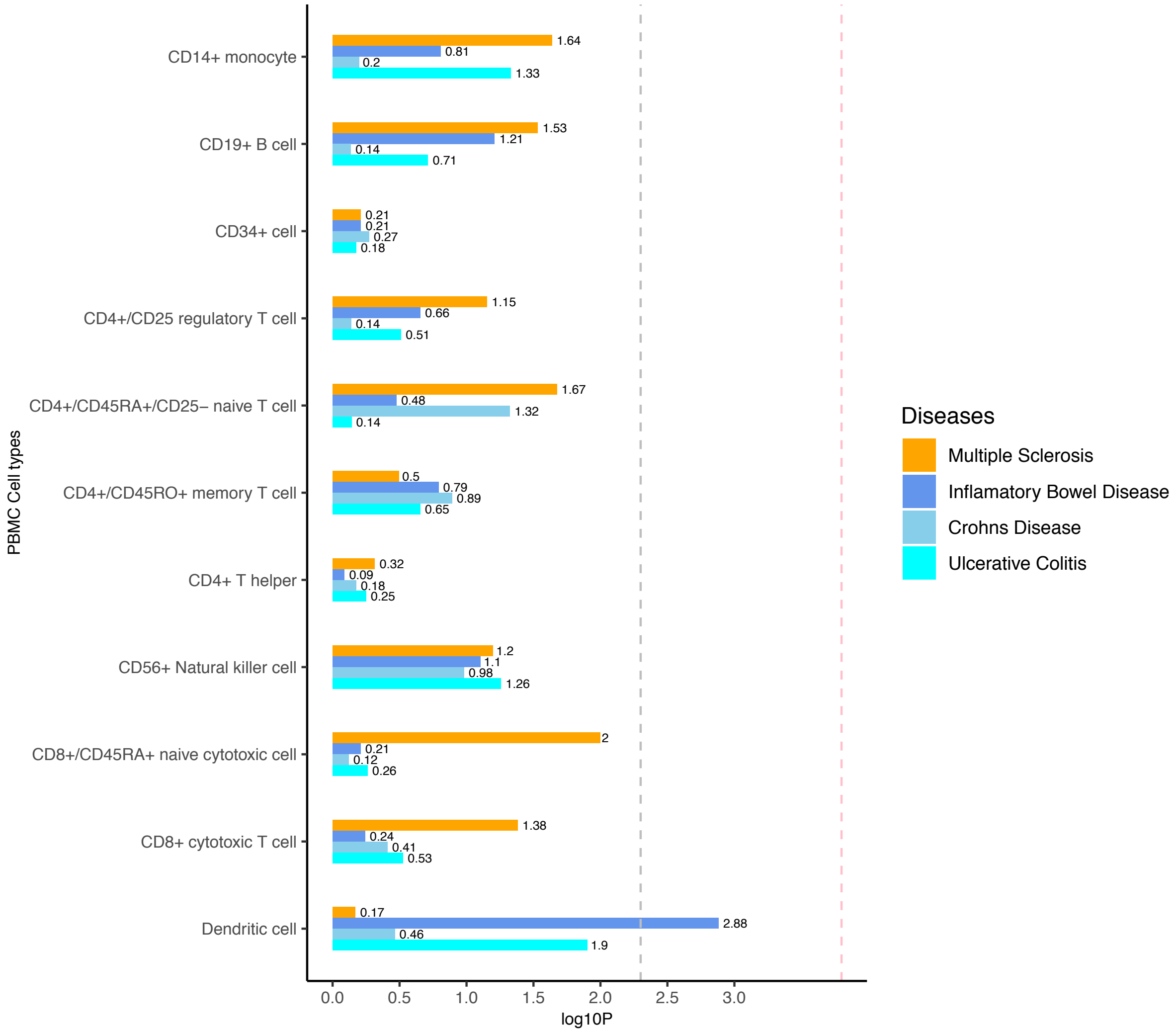

Figure S25. MAGMA (Multi-marker Analysis of GenoMic)-based heritability enrichment estimates in 11 peripheral blood mononuclear cells (PBMC) cell types[6] for multiple sclerosis (MS) and each of inflammatory bowel disease (IBD), ulcerative colitis (UC) and Crohn's disease (CD), including genes in the major histocompatibility complex (MHC) region. Negative log10 *p*-values of coefficient *Z*-scores for each individual test (two-tailed *Z*-test) are displayed on the x axis. The grey and pink dotted lines represent the false discovery rate (FDR) <5% and Bonferroni corrected thresholds for multiple regressions, respectively.

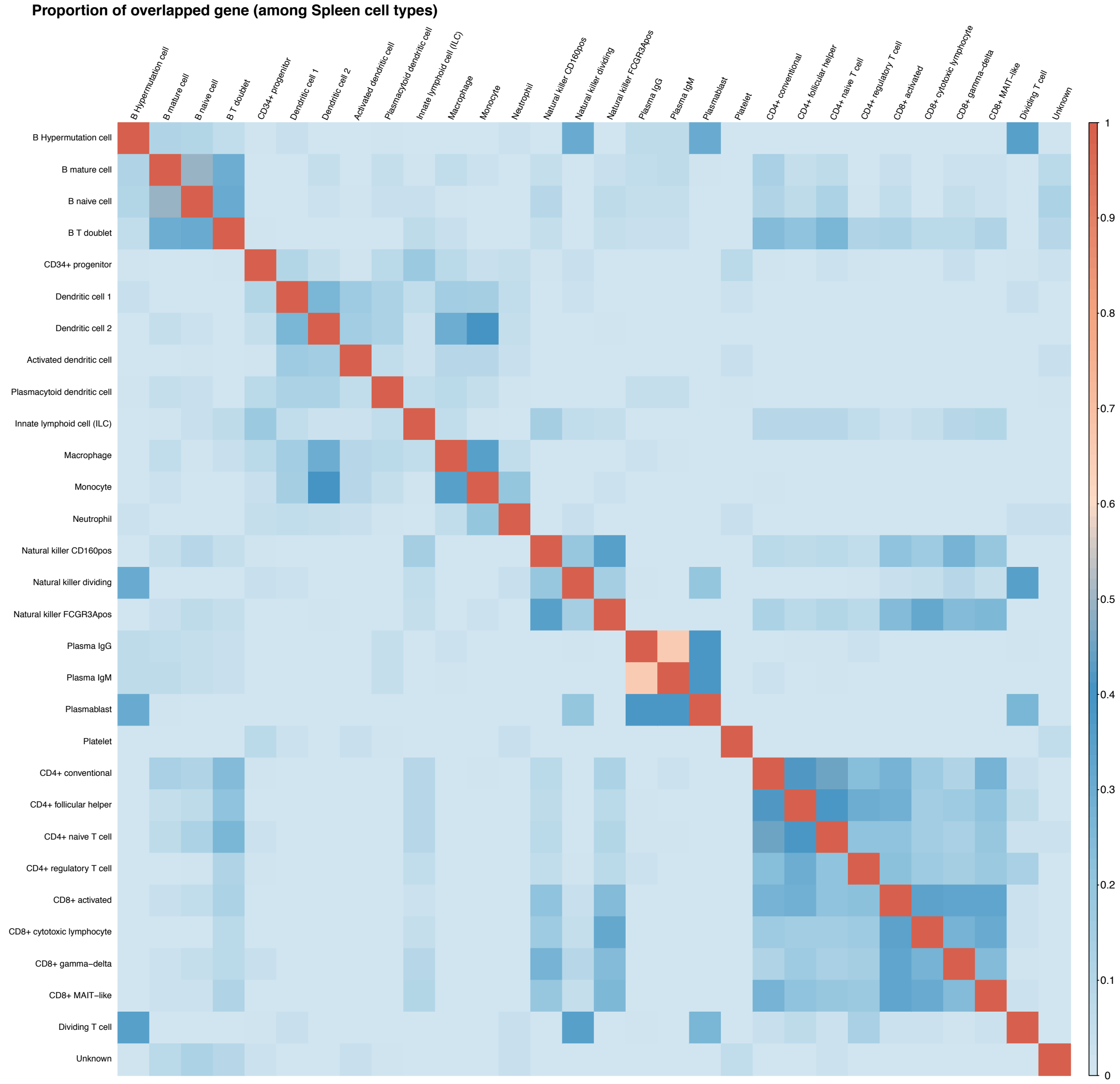

Figure S26. Proportion of overlap of the top 10% most specific genes among 30 spleen cell types[5], using the Bryois et al. (2020) method.

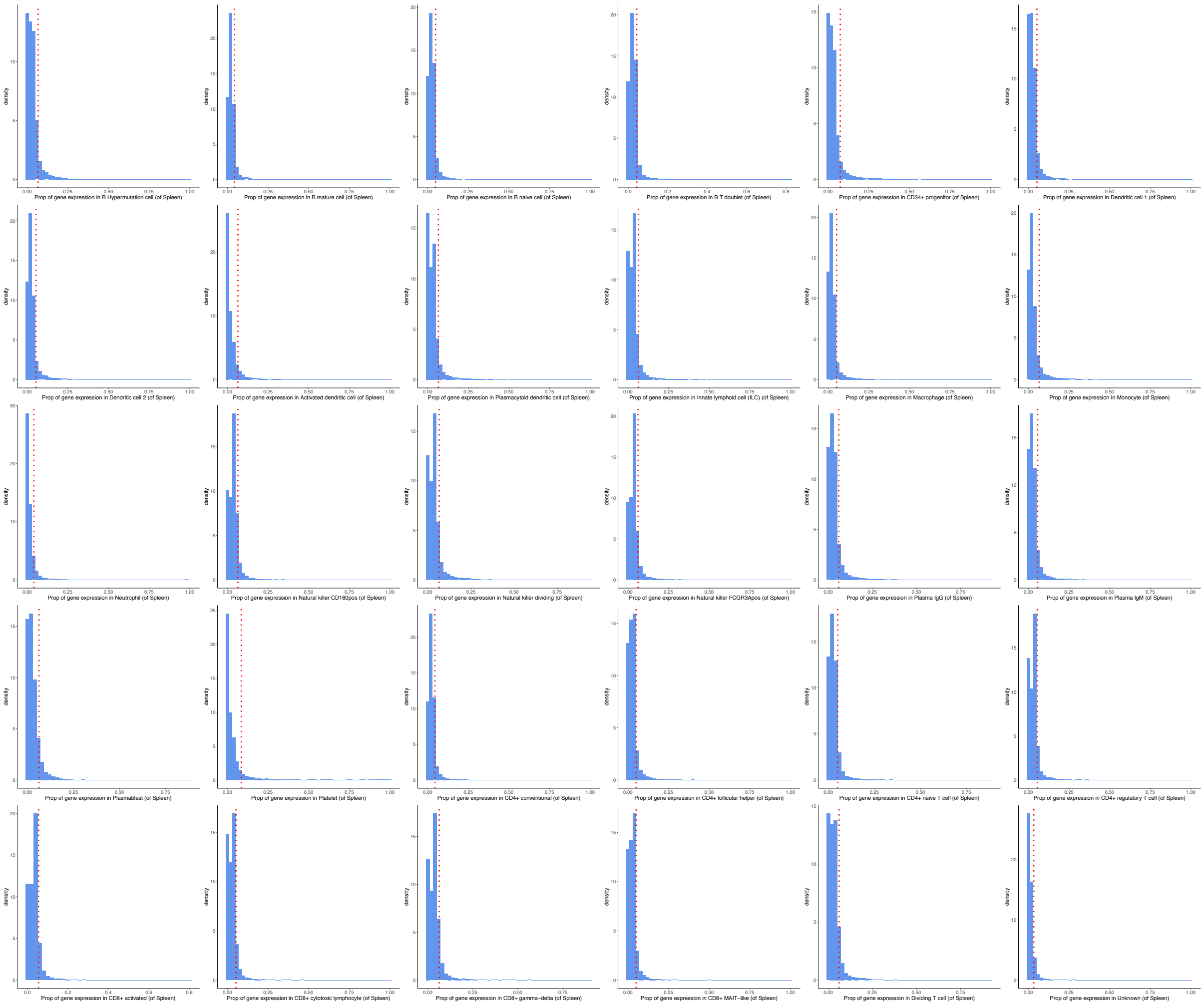

Figure S27. Distribution of the proportion of total expression per gene in each of the 30 spleen cell types[5], using the Bryois et al. (2020) method for defining cell type-specific genes. For each cell type, the top 10% most cell type-specific genes are distributed in the right of the red dotted vertical line.

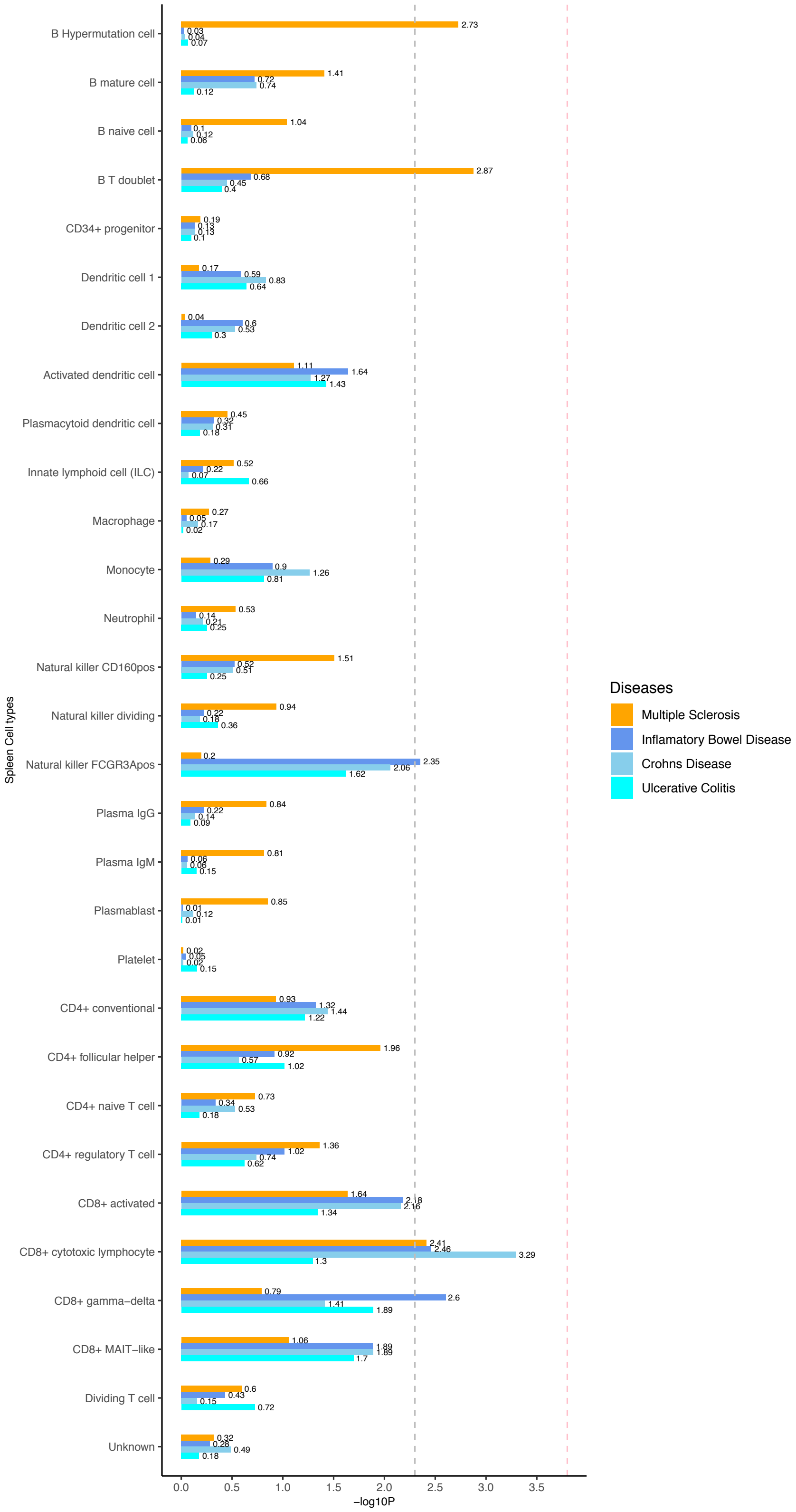

Figure S28. Stratified linkage disequilibrium score regression (S-LDSC)-based heritability enrichment estimates in 30 spleen cell types[5] for multiple sclerosis (MS) and each of inflammatory bowel disease (IBD), ulcerative colitis (UC) and Crohn's disease (CD). Negative log10 *p*-values of coefficient *Z*-scores for each individual test (two-tailed *Z*-test) are displayed on the x axis. The grey and pink dotted lines represent the false discovery rate (FDR) <5% and Bonferroni corrected thresholds for multiple regressions, respectively.

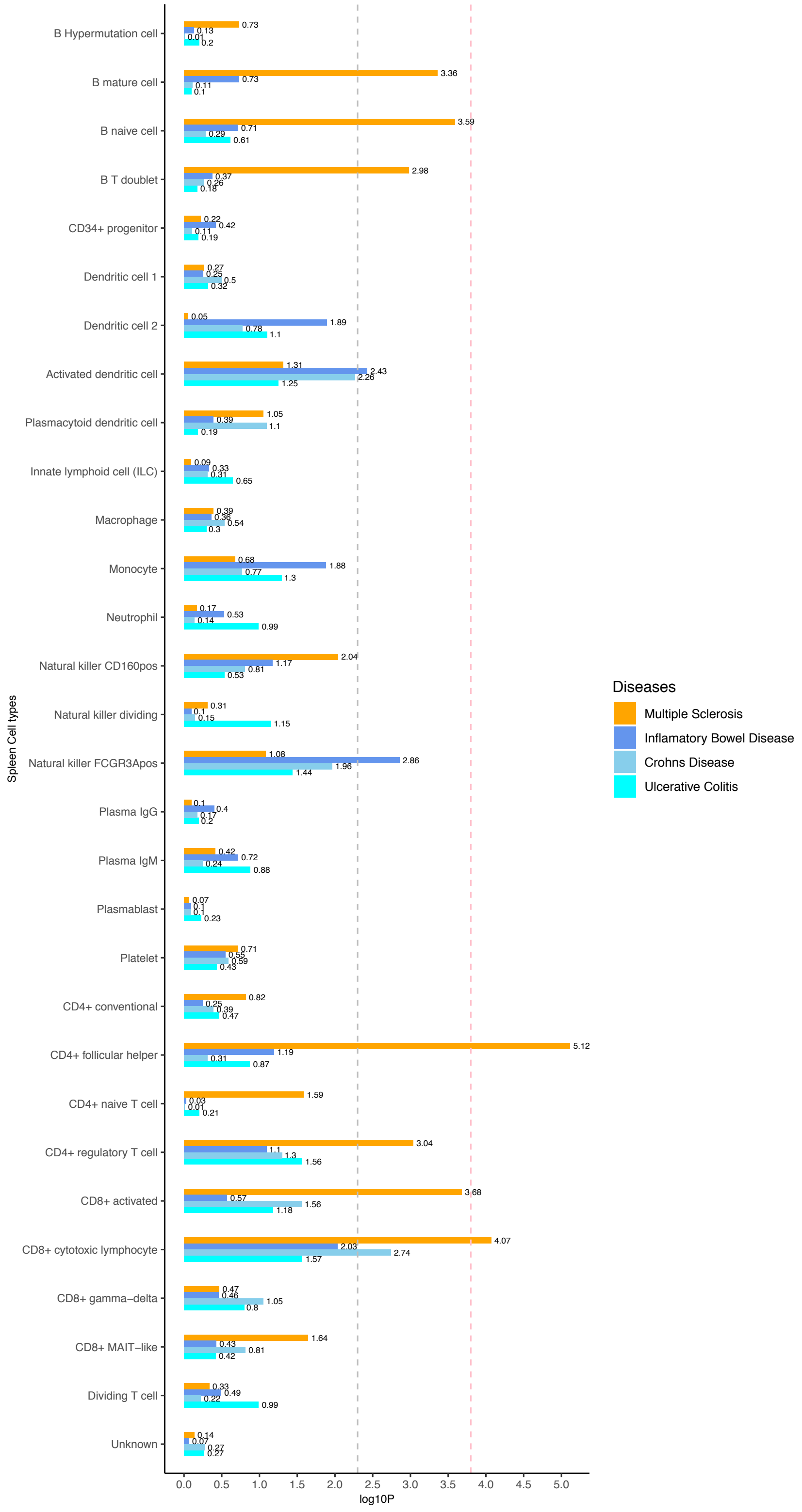

Figure S29. MAGMA (Multi-marker Analysis of GenoMic)-based heritability enrichment in 30 spleen cell types[5] for multiple sclerosis (MS) and each of inflammatory bowel disease (IBD), ulcerative colitis (UC) and Crohn's disease (CD), without genes in the major histocompatibility complex (MHC) region. Negative log10 *p*-values of coefficient *Z*-scores for each individual test (two-tailed *Z*-test) are displayed on the x axis. The grey and pink dotted lines represent the false discovery rate (FDR) <5% and Bonferroni corrected thresholds for multiple regressions, respectively.

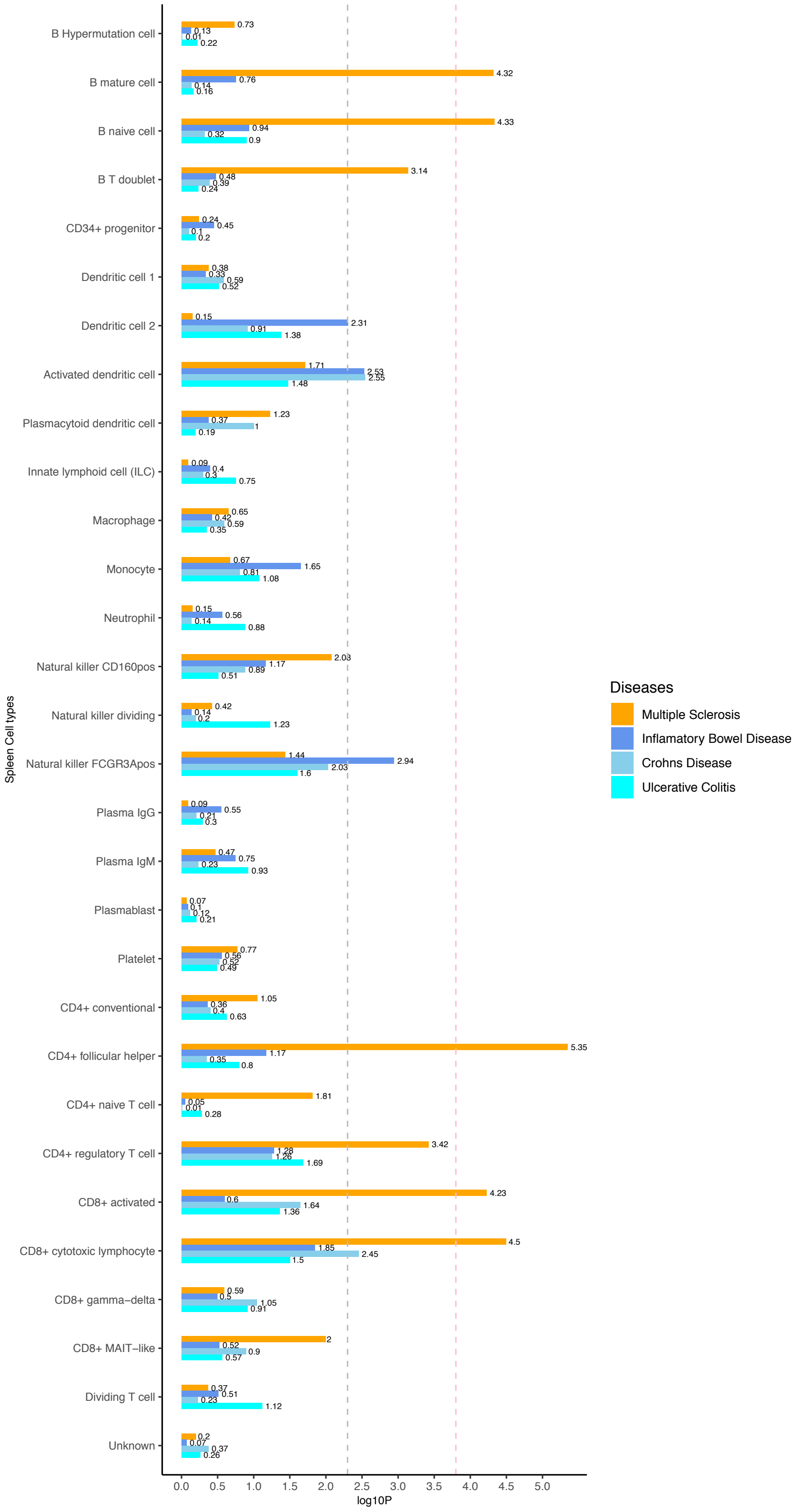

Figure S30. MAGMA (Multi-marker Analysis of GenoMic)-based heritability enrichment estimates in 30 spleen cell types[5] for multiple sclerosis (MS) and each of inflammatory bowel disease (IBD), ulcerative colitis (UC) and Crohn's disease (CD), including genes in the major histocompatibility complex (MHC) region. Negative log10 *p*-values of coefficient *Z*-scores for each individual test (two-tailed *Z*-test) are displayed on the x axis. The grey and pink dotted lines represent the false discovery rate (FDR) <5% and Bonferroni corrected thresholds for multiple regressions, respectively.

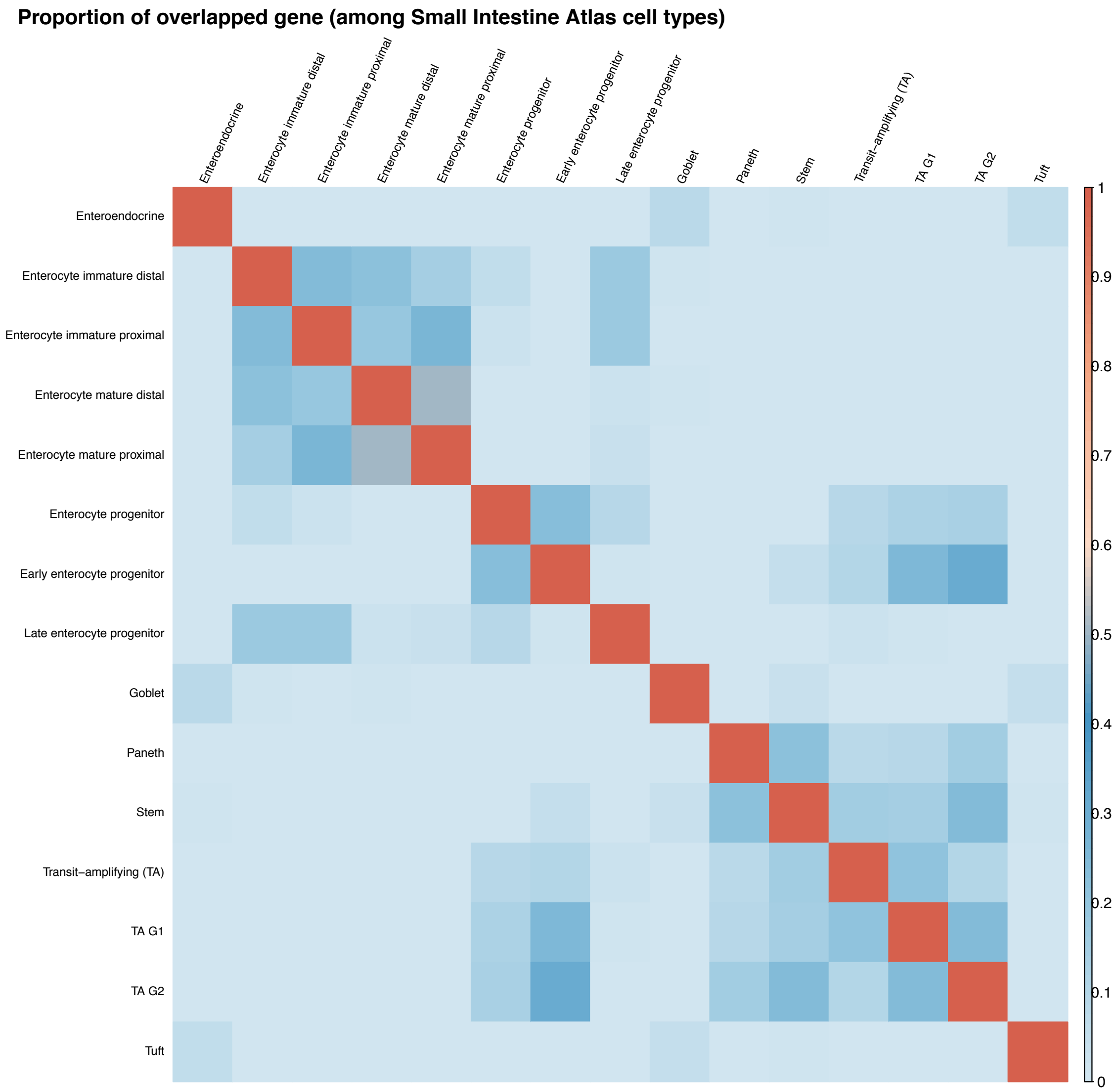

Figure S31. Proportion of overlap of the top 10% most specific genes among 15 small intestine cell types[7], using the Bryois et al. (2020) method.

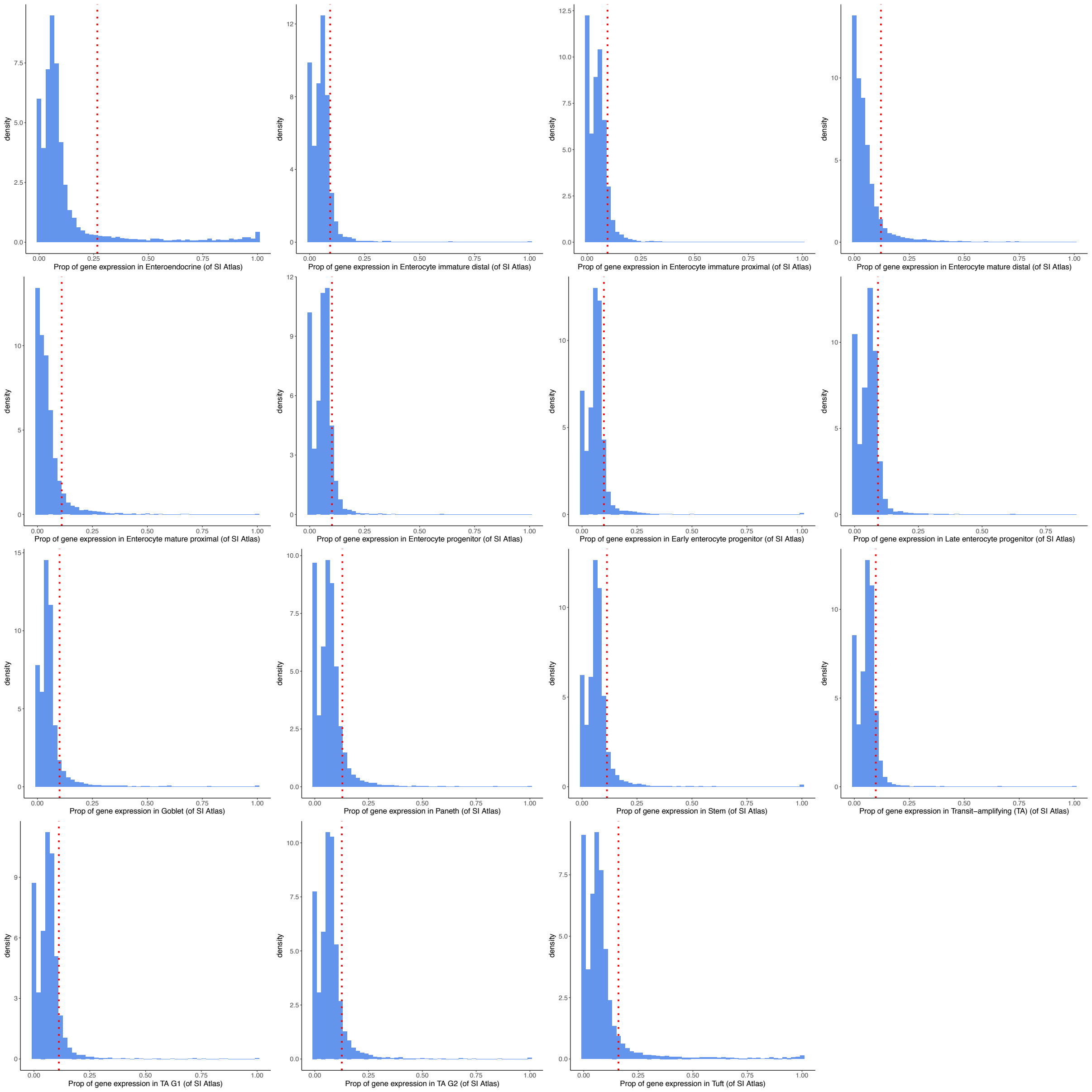

Figure S32. Distribution of the proportion of total expression per gene in each of the 15 small intestine cell types[7], using the Bryois et al. (2020) method for defining cell type-specific genes. For each cell type, the top 10% most cell type-specific genes are distributed in the right of the red dotted vertical line.

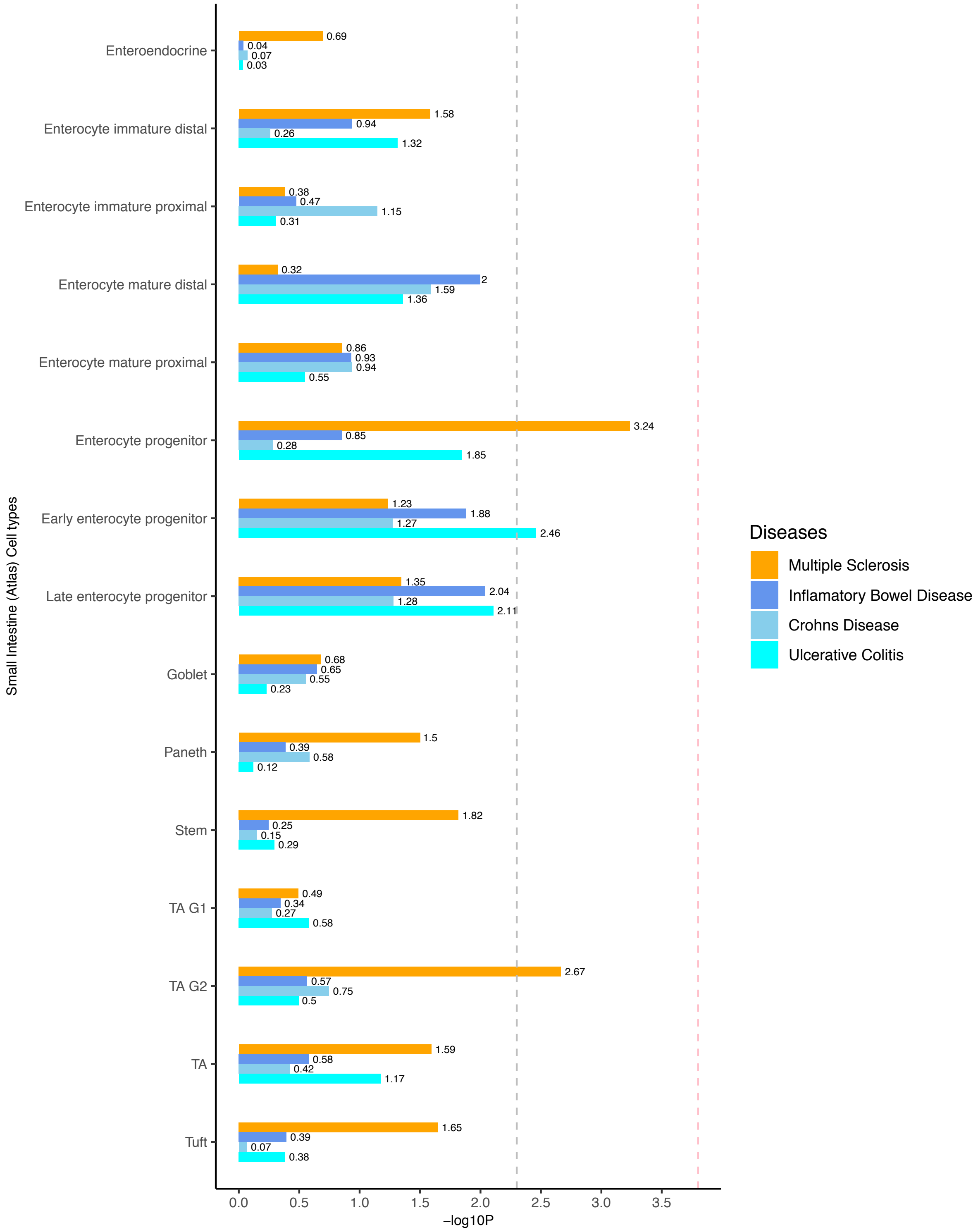

Figure S33. Stratified linkage disequilibrium score regression (S-LDSC)-based heritability enrichment estimates in 15 small intestine cell types[7] for multiple sclerosis (MS) and each of inflammatory bowel disease (IBD), ulcerative colitis (UC) and Crohn's disease (CD). Negative log10 *p*-values of coefficient *Z*-scores for each individual test (two-tailed *Z*-test) are displayed on the x axis. The grey and pink dotted lines represent the false discovery rate (FDR) <5% and Bonferroni corrected thresholds for multiple regressions, respectively.

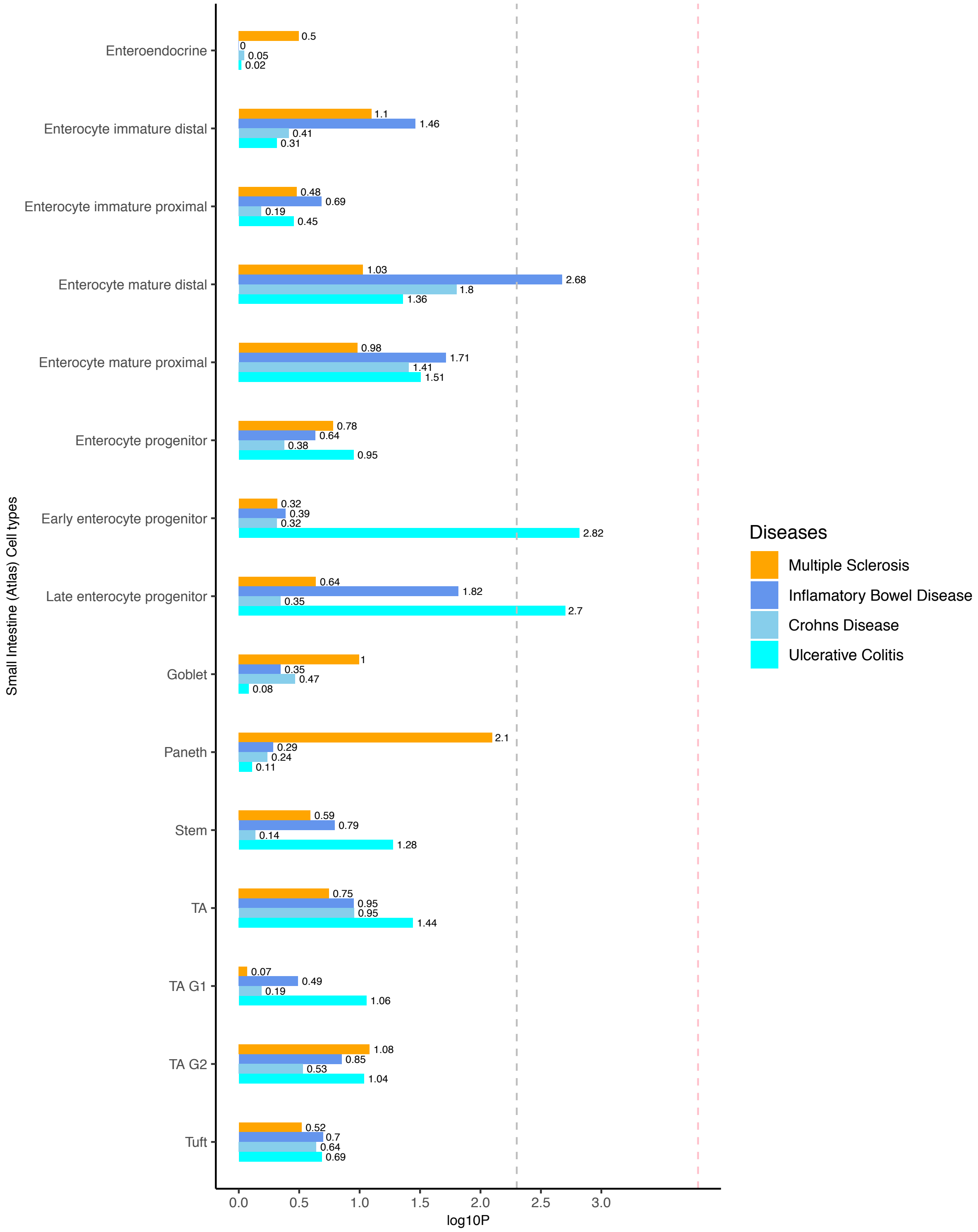

Figure S34. MAGMA (Multi-marker Analysis of GenoMic)-based heritability enrichment in 15 small intestine cell types[7] for multiple sclerosis (MS) and each of inflammatory bowel disease (IBD), ulcerative colitis (UC) and Crohn's disease (CD), without genes in the major histocompatibility complex (MHC) region. Negative log10 *p*-values of coefficient *Z*-scores for each individual test (two-tailed *Z*-test) are displayed on the x axis. The grey and pink dotted lines represent the false discovery rate (FDR) <5% and Bonferroni corrected thresholds for multiple regressions, respectively.

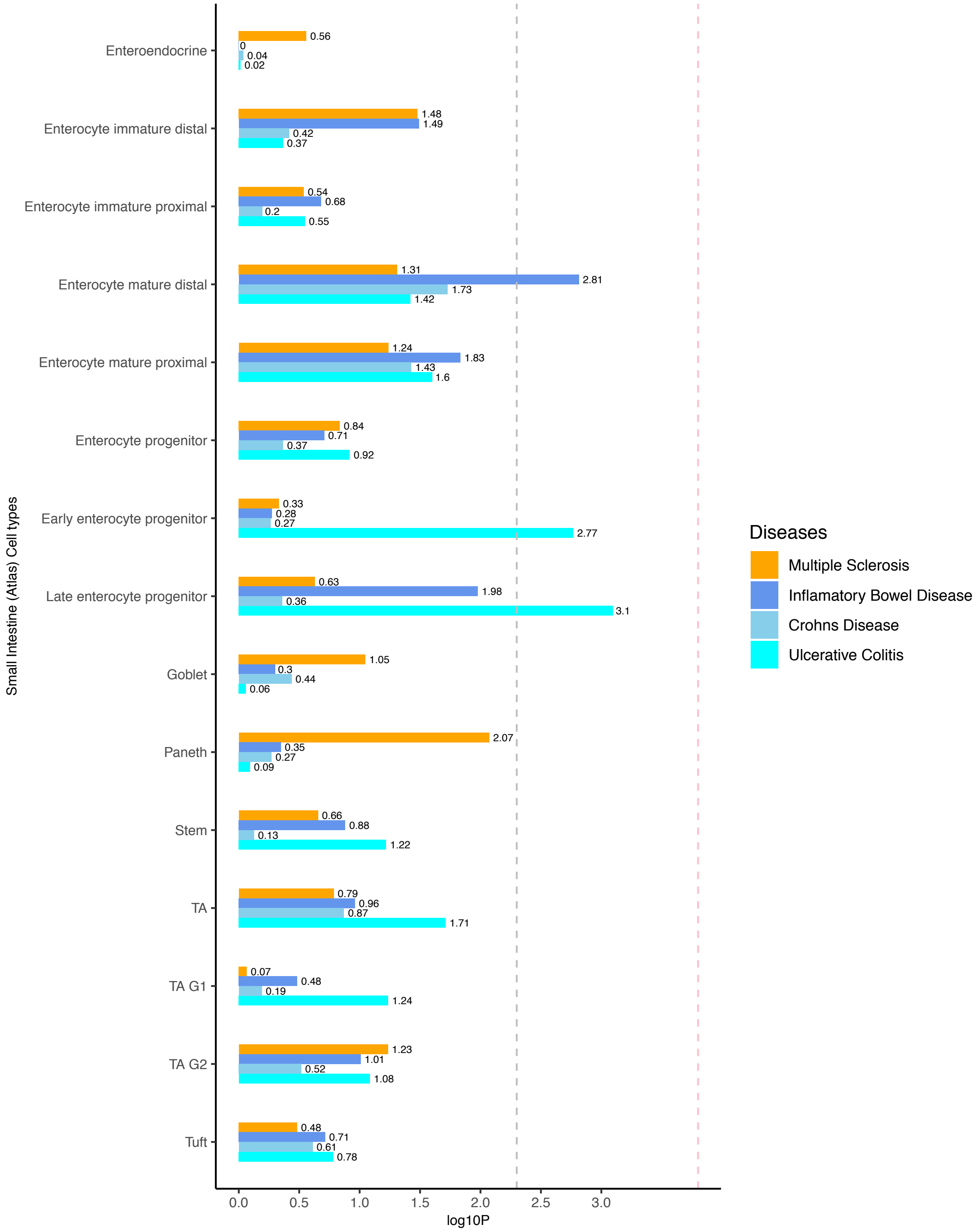

Figure S35. MAGMA (Multi-marker Analysis of GenoMic)-based heritability enrichment estimates in 15 small intestine cell types[7] for multiple sclerosis (MS) and each of inflammatory bowel disease (IBD), ulcerative colitis (UC) and Crohn's disease (CD), including genes in the major histocompatibility complex (MHC) region. Negative log10 *p*-values of coefficient *Z*-scores for each individual test (two-tailed *Z*-test) are displayed on the x axis. The grey and pink dotted lines represent the false discovery rate (FDR) <5% and Bonferroni corrected thresholds for multiple regressions, respectively.

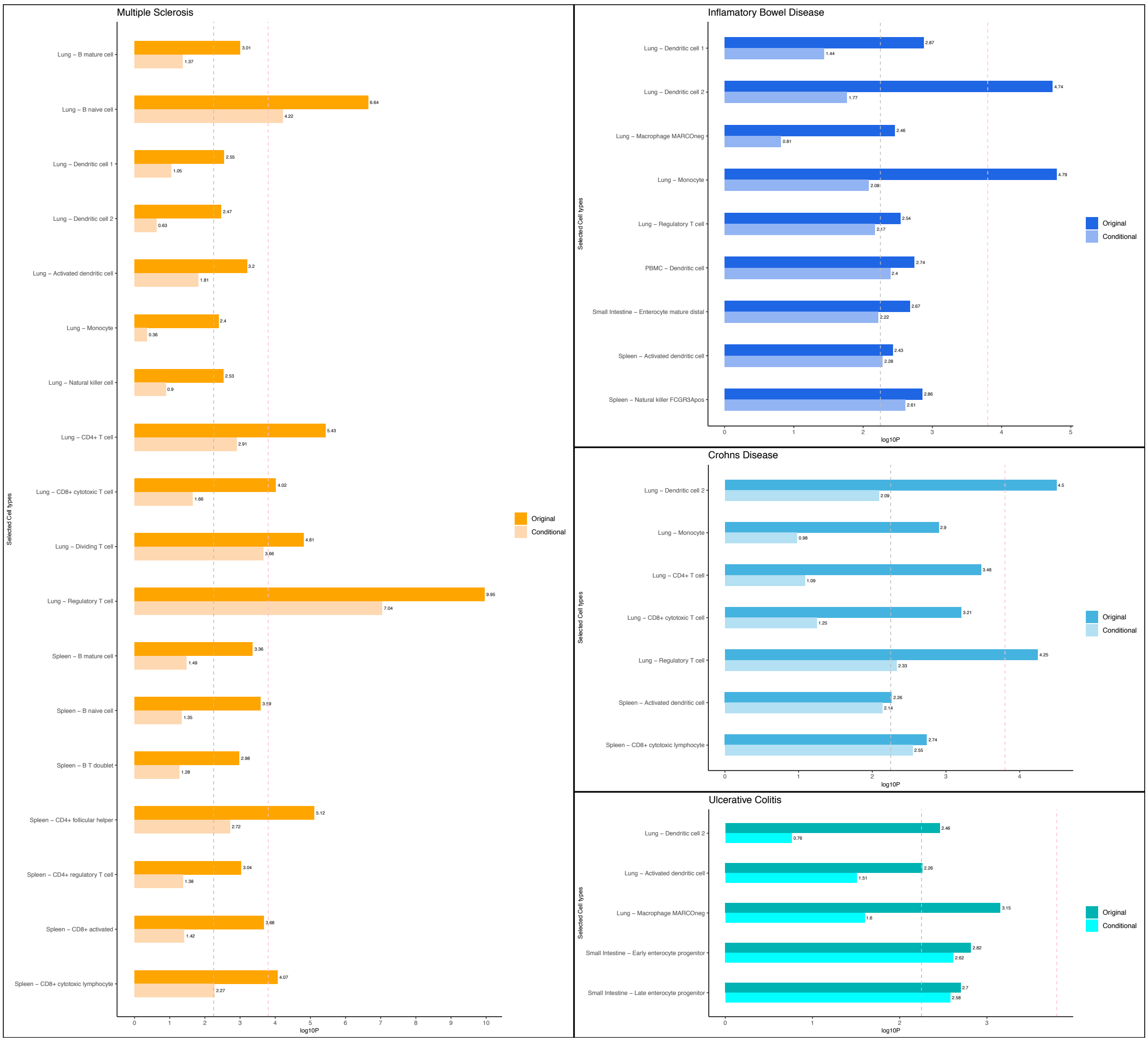

Figure S36. Cell type-specific enrichment of heritability for multiple sclerosis (MS), inflammatory bowel disease (IBD), ulcerative colitis (UC) and Crohn's disease (CD) in immune tissue cells using MAGMA (Multi-marker Analysis of GenoMic Annotation, without genes in the major histocompatibility complex [MHC] region). Cell types are included if they showed FDR-significant enrichment in at least one disease. Negative log10 *p*-values of coefficient *Z*-scores for each individual test (two-tailed *Z*-test) are displayed on the x axis. The grey and pink dotted lines represent the false discovery rate (FDR) <5% and Bonferroni corrected thresholds for multiple regressions, respectively. Original indicates results from analyses adjusted for the baseline model and the set of all genes. Conditional indicates results from conditional analyses adjusted for the baseline model, the set of all genes, the set of genes specifically expressed in the three non-focal tissues (e.g. small intestine - terminal ileum, lung and whole blood in analyses of spleen) and the set of genes highly expressed in other FDR-significant cell types of the same tissue and same disease (e.g. spleen: B/T doublet and spleen: $CD8^{+}$ cytotoxic lymphocytes in analyses of spleen: B hypermutation cells).

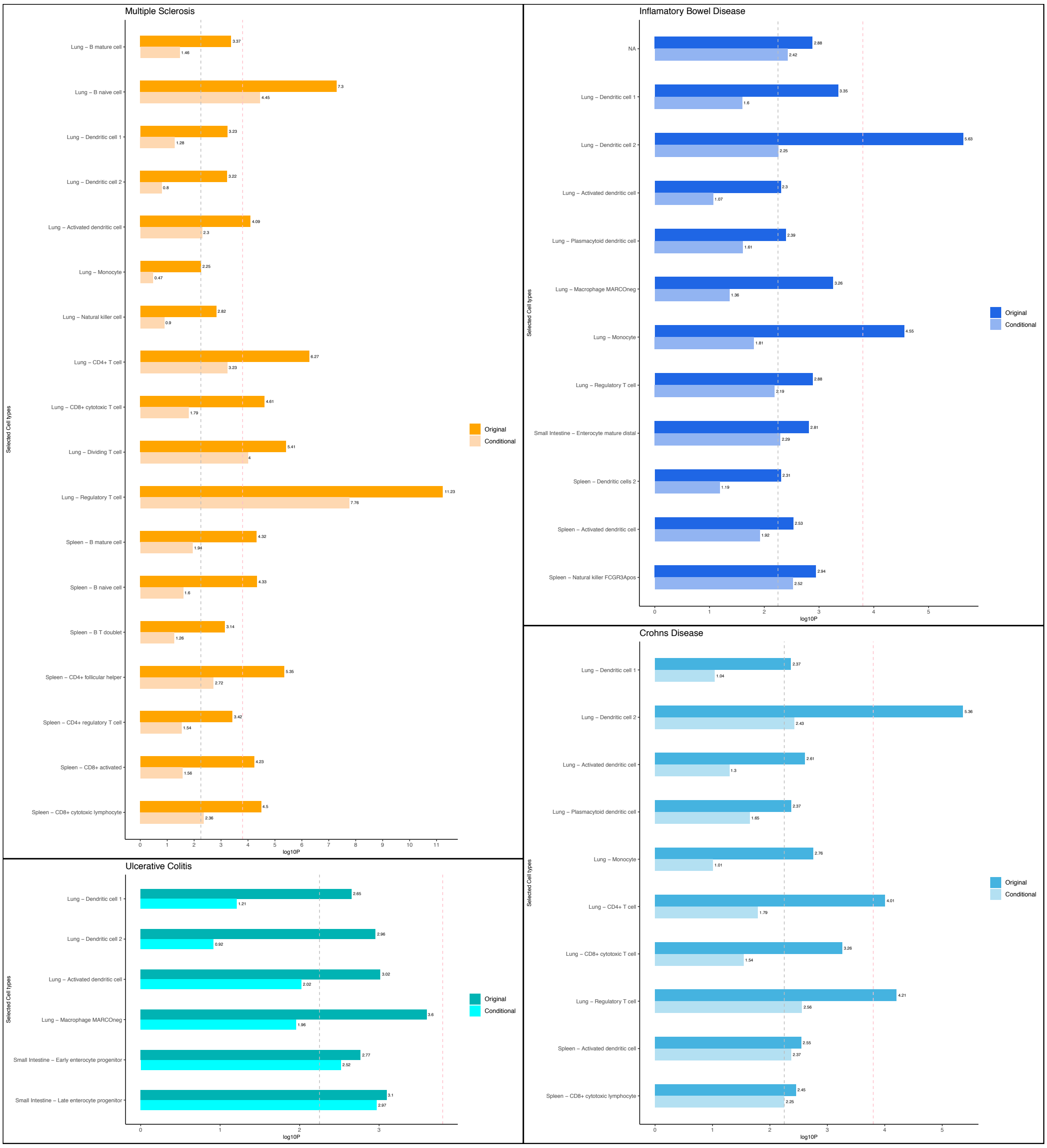

Figure S37. Cell type-specific enrichment of heritability for multiple sclerosis (MS), inflammatory bowel disease (IBD), ulcerative colitis (UC) and Crohn's disease (CD) in immune tissue cells using MAGMA (Multi-marker Analysis of GenoMic Annotation, including genes in the major histocompatibility complex [MHC] region). Cell types are included if they showed false discovery rate (FDR)-significant enrichment in at least one disease. Negative log10 *p*-values of coefficient *Z*-scores for each individual test (two-tailed *Z*-test) are displayed on the x axis. The grey and pink dotted lines represent the FDR <5% and Bonferroni corrected thresholds for multiple regressions, respectively. Original indicates results from analyses adjusted for the baseline model and the set of all genes. Conditional indicates results from conditional analyses adjusted for the baseline model, the set of all genes, the set of genes specifically expressed in the three non-focal tissues (e.g. small intestine - terminal ileum, lung and whole blood in analyses of spleen) and the set of genes highly expressed in other FDR-significant cell types of the same tissue and same disease (e.g. spleen: B/T doublet and spleen: $CD8^+$ cytotoxic lymphocytes in analyses of spleen: B hypermutation cells).

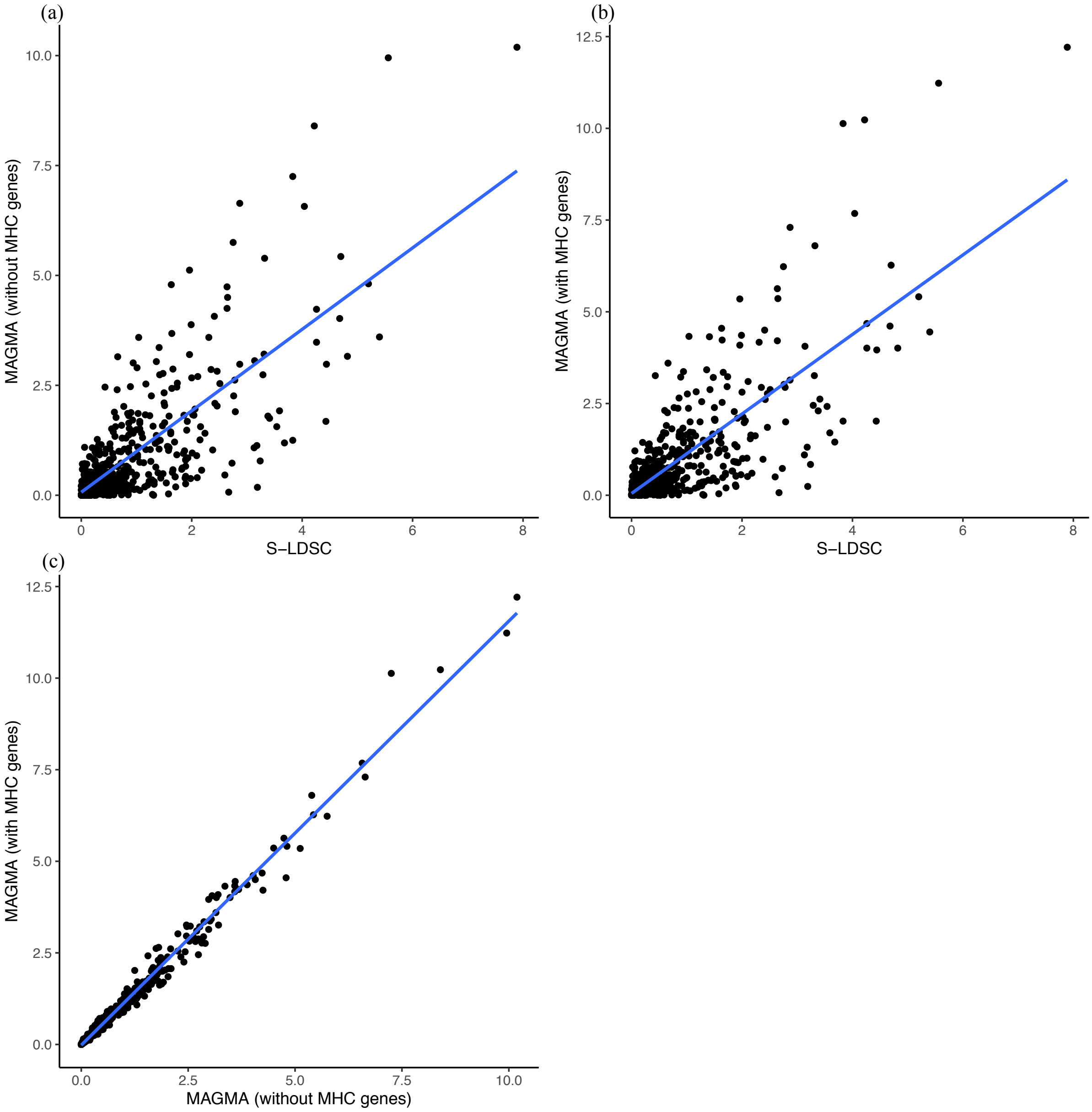

Figure S38. Comparison of heritability enrichment estimates (for results of both tissues and cell types; i.e. negative log10 *p*-values of coefficient *Z*-scores for each individual test [two-tailed *Z*-test]) estimated by: (a) stratified linkage disequilibrium score regression (S-LDSC) and Multi-marker Analysis of GenoMic Annotation (MAGMA, without genes in the major histocompatibility complex [MHC] region); (b) S-LDSC and MAGMA (including genes in the MHC region); (c) MAGMA with and without genes in the MHC region. Blue line shows the regression line of the x axis variable against the y axis variable. The correlations between the heritability enrichments estimated by S-PLDSC and MAGMA (with/without genes in the MHC region) are ~0.75 (with MHC genes: 95% confidence interval [CI]=0.70-0.78; Spearman's test *p*-value=1.04×10^{-88}; without MHC genes: 95% CI=0.71-0.79; Spearman's test *p*-value=1.60×10^{-89}), and the correlation between heritability enrichments estimated by MAGMA with and without genes in the MHC region is 0.99 (95% CI=0.99-1.00; Spearman's test *p*-value$<1\times10^{-300}$).

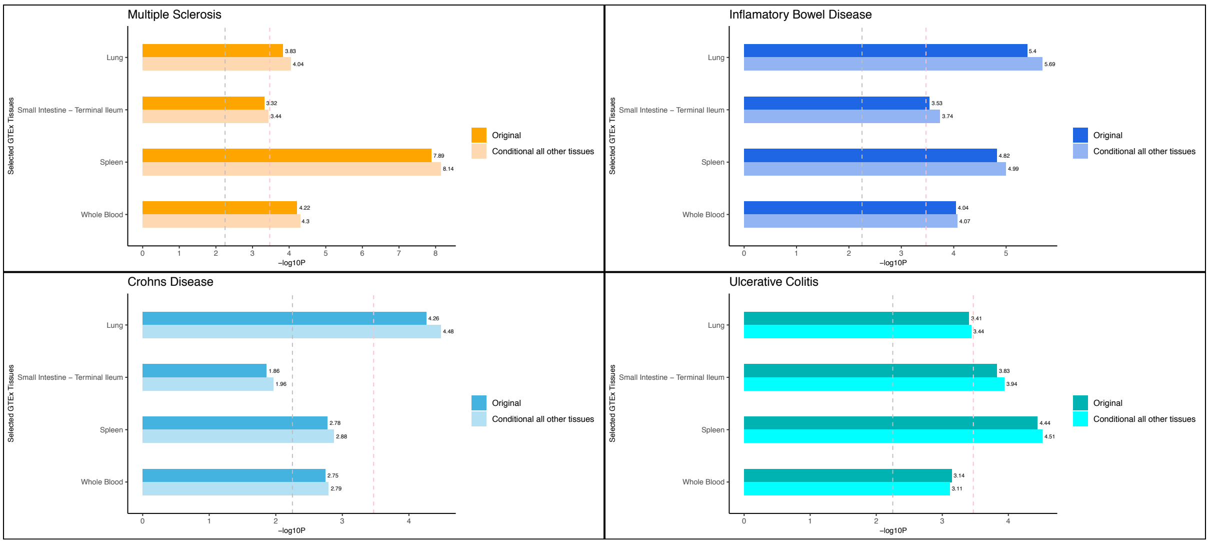

Figure S39. Tissue-specific enrichment of heritability for multiple sclerosis (MS), inflammatory bowel disease (IBD), ulcerative colitis (UC) and Crohn's disease (CD) in immune tissues, using stratified linkage disequilibrium score regression (S-LDSC). Negative log10 *p*-values of coefficient *Z*-scores for each individual test (two-tailed *Z*-test) are displayed on the x axis. The grey and pink dotted lines represent the false discovery rate (FDR) <5% and Bonferroni corrected thresholds for multiple regressions, respectively. Original indicates results of analyses adjusted for the baseline model and the set of all genes. Conditional indicates results from conditional analyses adjusted for the baseline model, the set of all genes and the set of genes specifically expressed in the other 36 non-focal tissues.

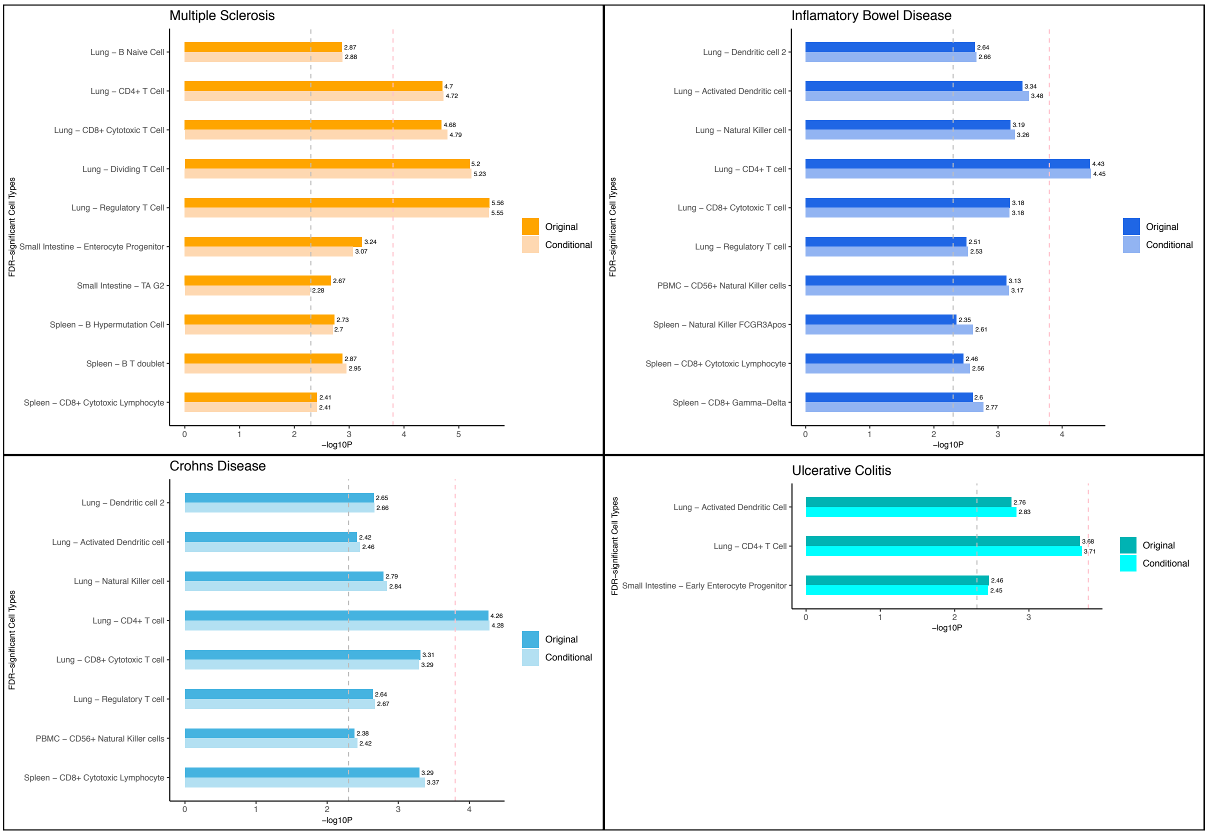

Figure S40. Cell type-specific enrichment of heritability for multiple sclerosis (MS), inflammatory bowel disease (IBD), ulcerative colitis (UC) and Crohn's disease (CD) in immune tissue cells, using stratified linkage disequilibrium score regression (S-LDSC). Cell types are included if they showed FDR-significant enrichments in at least one disease. Negative log10 *p*-values of coefficient *Z*-scores for each individual test (two-tailed *Z*-test) are displayed on the x axis. The grey and pink dotted lines represent the false discovery rate (FDR) <5% and Bonferroni corrected thresholds for multiple regressions, respectively. Original indicates results of analyses adjusted for the baseline model and the set of all genes. Conditional indicates results from conditional analyses that adjusted for the baseline model, the set of all genes, the set of genes specifically expressed in the other 36 non-focal tissues, and the set of genes specifically expressed in the other non-focal cell types of the same tissue disease.

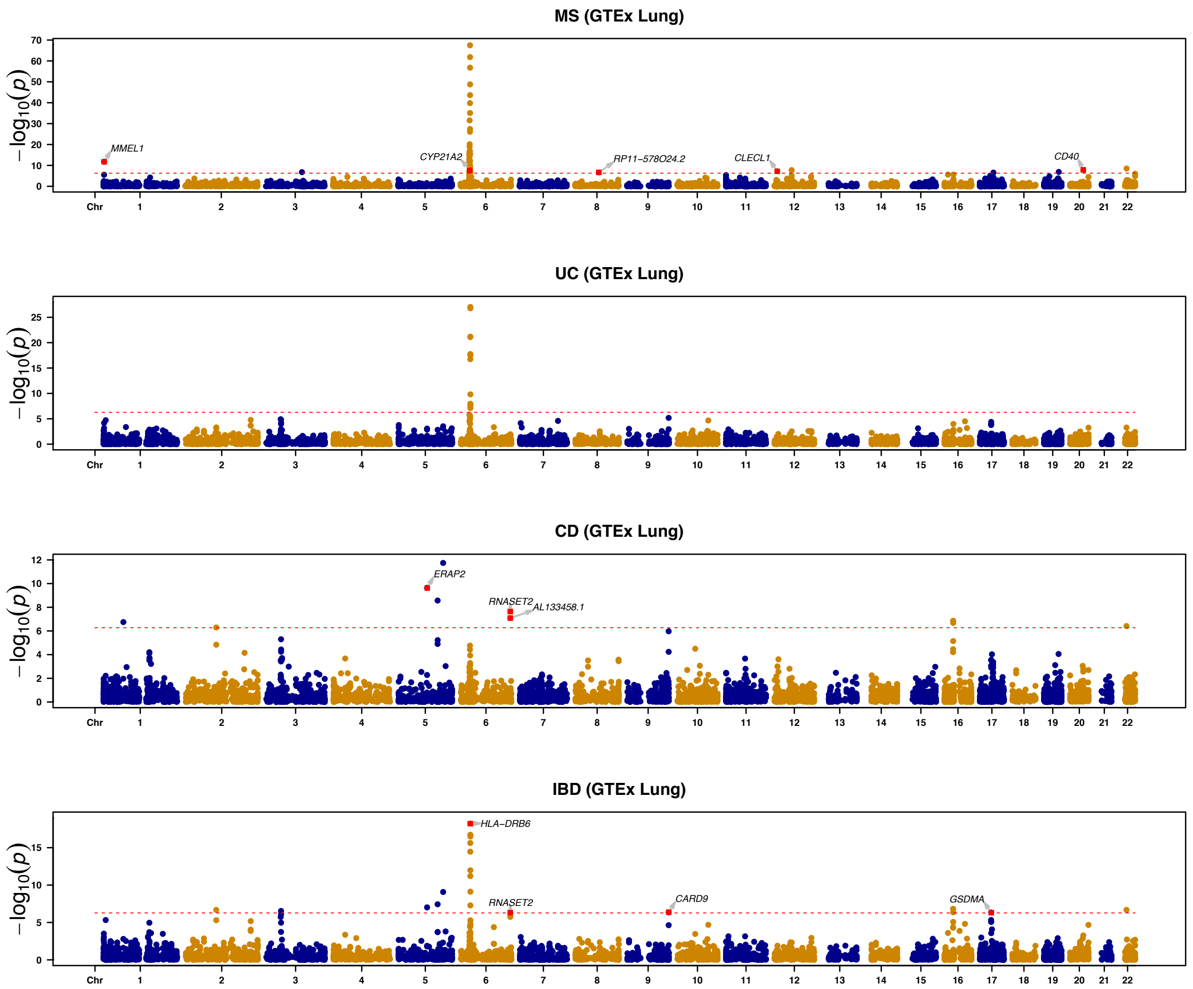

Figure S41. Manhattan plots of Summary-data-based Mendelian randomisation (SMR) results for associations between gene expression in lung (based on Genotype-Tissue Expression [GTEx] lung expression quantitative trait locus [eQTL] summary data) and each of multiple sclerosis (MS), inflammatory bowel disease (IBD), ulcerative colitis (UC) and Crohn's disease (CD). For each plot, the y-axis shows the –log10(SMR *p*-value [generated approximately by a two-tailed *Z*-test for each individual test]), the dotted horizontal line indicates the study-wide Bonferroni-corrected SMR threshold (SMR $p < 5.28 \times 10^{-7}$) for multiple regressions; and the genes in red represent the putative functional genes with HEIDI (HEterogeneity In Dependent Instrument) $p > 0.05$ and at least 10 SNPs after the HEIDI-outlier test.

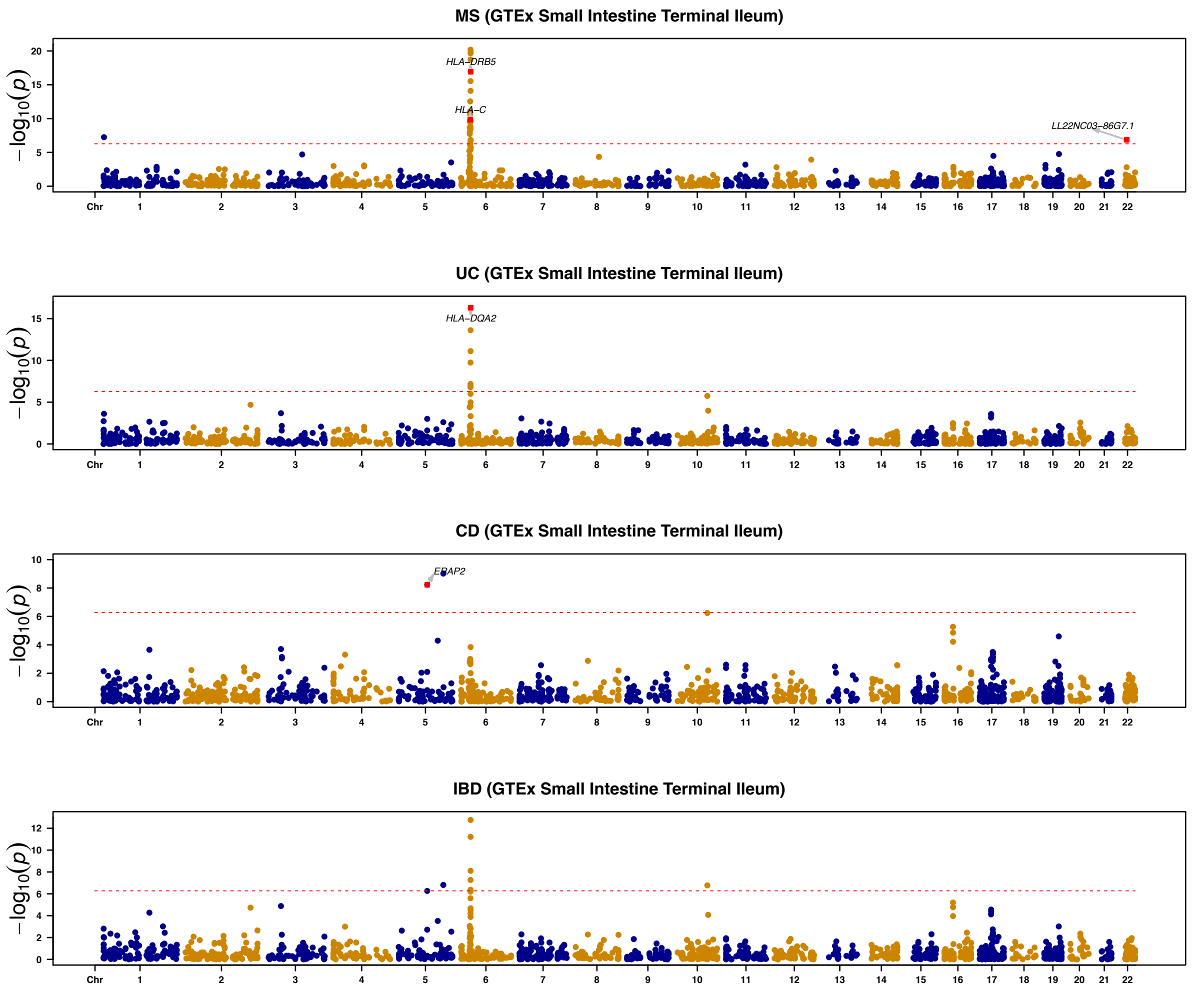

Figure S42. Manhattan plots of Summary-data-based Mendelian randomisation (SMR) results for associations between gene expression in small intestine-terminal ileum (based on Genotype-Tissue Expression [GTEx] small intestine-terminal ileum expression quantitative trait locus [eQTL] summary data) and each of multiple sclerosis (MS), inflammatory bowel disease (IBD), ulcerative colitis (UC) and Crohn’s disease (CD). For each plot, the y-axis shows the –log10(SMR *p*-value [generated approximately by a two-tailed *Z*-test for each individual test]), the dotted horizontal line indicates the study-wide Bonferroni-corrected SMR threshold (SMR $p < 5.28\times10^{-7}$) for multiple regressions, and the genes in red represent putative functional genes with HEIDI (HEterogeneity In Dependent Instrument) $p > 0.05$ and at least 10 SNPs after the HEIDI-outlier test.

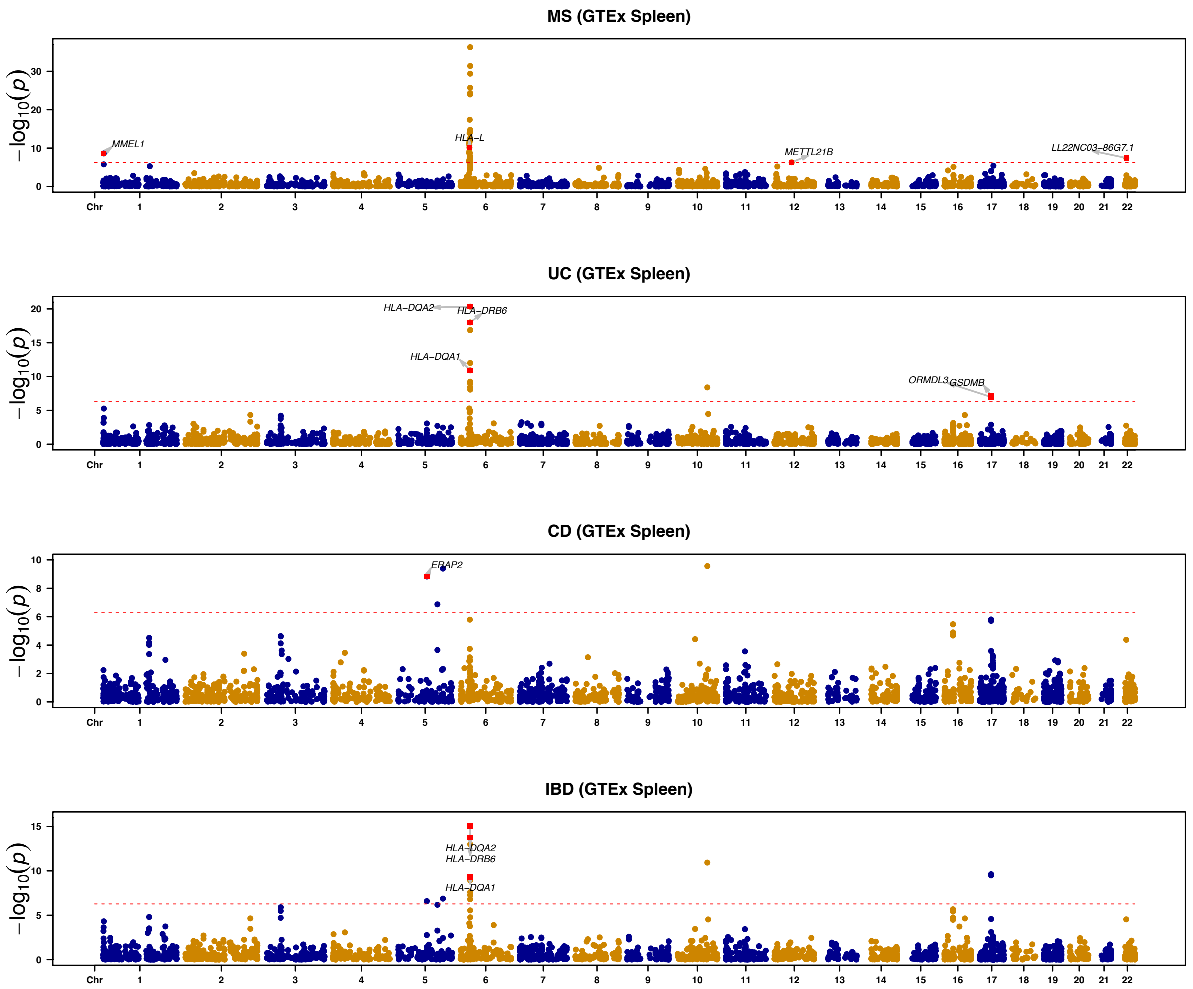

Figure S43. Manhattan plots of Summary-data-based Mendelian randomisation (SMR) results for associations between gene expression in spleen (based on Genotype-Tissue Expression [GTEx] spleen expression quantitative trait locus [eQTL] summary data) and each of multiple sclerosis (MS), inflammatory bowel disease (IBD), ulcerative colitis (UC) and Crohn's disease (CD). For each plot, the y-axis shows the –log10(SMR *p*-value [generated approximately by a two-tailed *Z*-test for each individual test]), the dotted horizontal line indicates the study-wide Bonferroni-corrected SMR threshold (SMR $p < 5.28 \times 10^{-7}$) for multiple regressions, and the genes in red represent putative functional genes with HEIDI (HEterogeneity In Dependent Instrument) $p > 0.05$ and at least 10 SNPs after the HEIDI-outlier test.

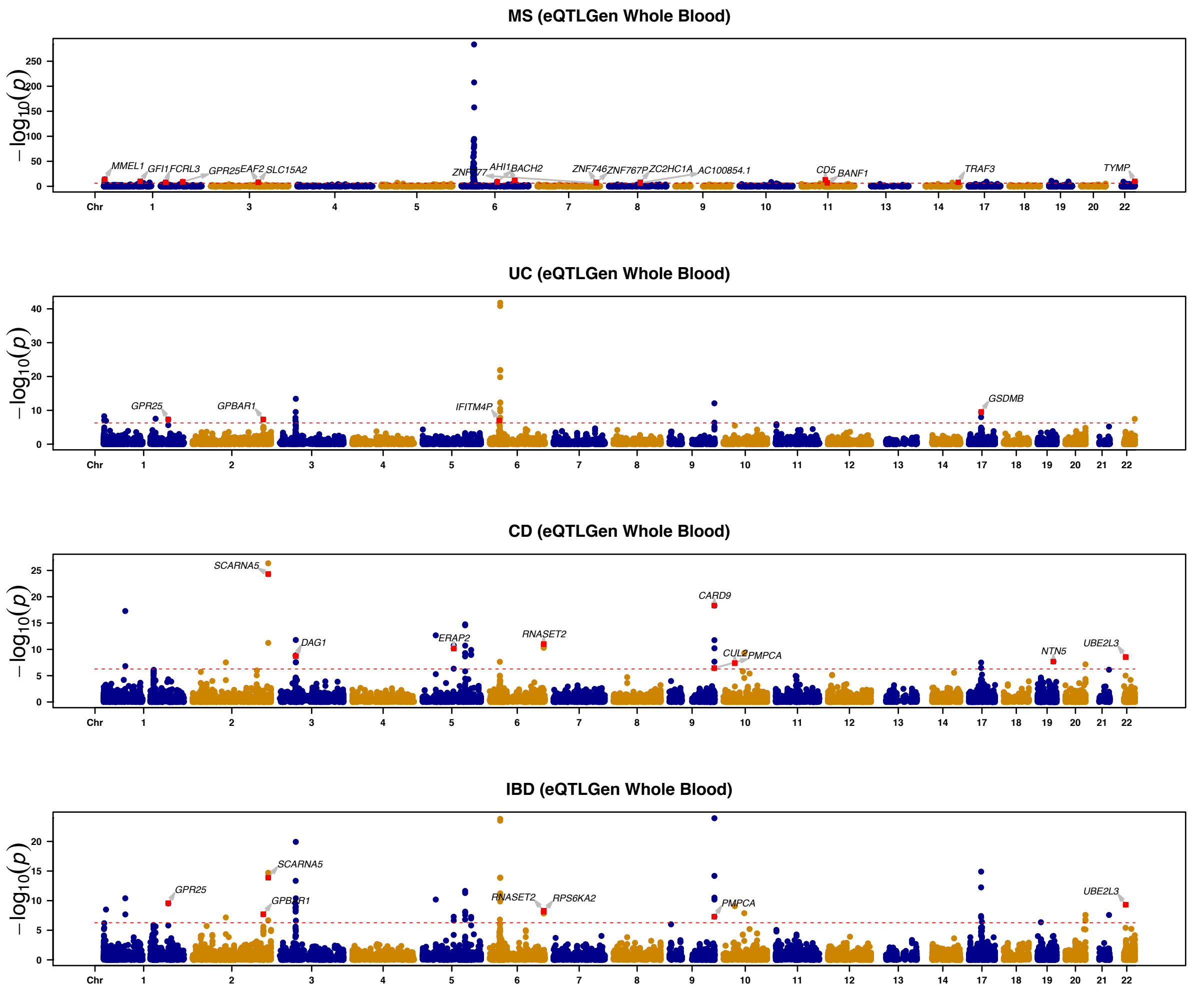

Figure S44. Manhattan plots of Summary-data-based Mendelian randomisation (SMR) results for associations between gene expression in whole blood (based on eQTLGen whole blood expression quantitative trait locus [eQTL] summary data) and each of multiple sclerosis (MS), inflammatory bowel disease (IBD), ulcerative colitis (UC) and Crohn's disease (CD). For each plot, the y-axis shows the –log10(SMR *p*-value [generated approximately by a two-tailed *Z*-test for each individual test]), the dotted horizontal line indicates the study-wide Bonferroni-corrected SMR threshold (SMR $p < 5.28\times10^{-7}$) for multiple regressions, and the genes in red represent putative functional genes with HEIDI (HEterogeneity In Dependent Instrument) $p > 0.05$ and at least 10 SNPs after the HEIDI-outlier test.

Reference

1. Bryois, J. *et al.* Genetic identification of cell types underlying brain complex traits yields insights into the etiology of Parkinson's disease. *Nat Genet* **52**, 482-493 (2020).
2. Finucane, H.K. *et al.* Heritability enrichment of specifically expressed genes identifies disease-relevant tissues and cell types. *Nat Genet* **50**, 621-629 (2018).
3. de Leeuw, C.A., Mooij, J.M., Heskes, T. & Posthuma, D. MAGMA: generalized gene-set analysis of GWAS data. *PLoS Comput Biol* **11**, e1004219 (2015).
4. GTEx Consortium. The Genotype-Tissue Expression (GTEx) project. *Nat Genet* **45**, 580-5 (2013).
5. Madissoon, E. *et al.* scRNA-seq assessment of the human lung, spleen, and esophagus tissue stability after cold preservation. *Genome Biol* **21**, 1 (2019).
6. Zheng, G.X. *et al.* Massively parallel digital transcriptional profiling of single cells. *Nat Commun* **8**, 14049 (2017).
7. Haber, A.L. *et al.* A single-cell survey of the small intestinal epithelium. *Nature* **551**, 333-339 (2017).
