# Supplementary material for: Investigating the shared genetic architecture between multiple sclerosis and inflammatory bowel diseases
Source: Nat Commun. 2021 Sep 24;12:5641. doi: 10.1038/s41467-021-25768-0 (PMC8463615; doi:10.1038/s41467-021-25768-0)
Supplement: Supplementary file 3 — Description of Additional Supplementary Files [file 41467_2021_25768_MOESM3_ESM.pdf]

Description of Additional Supplementary Files

File Name: Supplementary Data 1
Description: Cell types and number of cells per cell type in scRNA-seq data from lung, peripheral blood, small intestine and spleen

File Name: Supplementary Data 2
Description: Summary of SNP heritability enrichment in 37 GTEx tissues for MS, and each of IBD, UC, and CD using the Bryois et al. (2020) method

File Name: Supplementary Data 3
Description: Summary of SNP heritability enrichment in 45 GTEx tissues for MS, and each of IBD, UC, and CD using the Finucane et al. (2018) method

File Name: Supplementary Data 4
Description: Summary of SNP heritability enrichment in 84 cell types for MS and each of IBD, UC, and CD using the Bryois et al. (2020) method

File Name: Supplementary Data 5
Description: Summary of conditional SNP heritability enrichment in FDR-significant GTEx tissues for MS, and each of IBD, UC, and CD using the Bryois et al. (2020) method

File Name: Supplementary Data 6
Description: Summary of conditional SNP heritability enrichment in FDR-significant cell types for MS, and each of IBD, UC, and CD using the Bryois et al. (2020) method

File Name: Supplementary Data 7
Description: Summary of gene-level analyses in 37 GTEx tissues for MS, and each of IBD, UC, and CD using MAGMA

File Name: Supplementary Data 8
Description: Summary of gene-level analyses in 84 cell types for MS, and each of IBD, UC, and CD using MAGMA

File Name: Supplementary Data 9
Description: Summary of gene-level conditional analyses in FDR-significant GTEx tissues for MS, and each of IBD, UC, and CD using MAGMA

File Name: Supplementary Data 10
Description: Summary of gene-level conditional analyses in FDR-significant cell types for MS, and each of IBD, UC, and CD using MAGMA

File Name: Supplementary Data 11
Description: Summary of significant non-MHC SMR associations in MS and each of IBD, UC, and CD using eQTL data from eQTLGen and GTEx tissues

File Name: Supplementary Data 12
Description: Summary of significant MHC SMR associations for MS and each of IBD, UC, and CD using eQTL data from eQTLGen and GTEx tissues
