# Supplementary material for: Investigating the shared genetic architecture between multiple sclerosis and inflammatory bowel diseases
Source: Nat Commun. 2021 Sep 24;12:5641. doi: 10.1038/s41467-021-25768-0 (PMC8463615; doi:10.1038/s41467-021-25768-0)
Supplement: Supplementary file 16 — Reporting Summary [file 41467_2021_25768_MOESM16_ESM.pdf]

nature research

Corresponding author(s): Bruce Taylor, Jacob Gratten, Yuan Zhou

Last updated by author(s): Jul 26, 2021

Reporting Summary

Nature Research wishes to improve the reproducibility of the work that we publish. This form provides structure for consistency and transparency in reporting. For further information on Nature Research policies, see our Editorial Policies and the Editorial Policy Checklist.

Statistics

For all statistical analyses, confirm that the following items are present in the figure legend, table legend, main text, or Methods section.

n/a	Confirmed	
☐	☒	The exact sample size (n) for each experimental group/condition, given as a discrete number and unit of measurement
☐	☒	A statement on whether measurements were taken from distinct samples or whether the same sample was measured repeatedly
☐	☒	The statistical test(s) used AND whether they are one- or two-sided<br>*Only common tests should be described solely by name; describe more complex techniques in the Methods section.*
☐	☒	A description of all covariates tested
☐	☒	A description of any assumptions or corrections, such as tests of normality and adjustment for multiple comparisons
☐	☒	A full description of the statistical parameters including central tendency (e.g. means) or other basic estimates (e.g. regression coefficient) AND variation (e.g. standard deviation) or associated estimates of uncertainty (e.g. confidence intervals)
☐	☒	For null hypothesis testing, the test statistic (e.g. F, t, r) with confidence intervals, effect sizes, degrees of freedom and P value noted<br>*Give P values as exact values whenever suitable.*
☒	☐	For Bayesian analysis, information on the choice of priors and Markov chain Monte Carlo settings
☒	☐	For hierarchical and complex designs, identification of the appropriate level for tests and full reporting of outcomes
☐	☒	Estimates of effect sizes (e.g. Cohen's d, Pearson's r), indicating how they were calculated

Our web collection on statistics for biologists contains articles on many of the points above.

Software and code

Policy information about availability of computer code

Data collection	No software was used for data collection.
Data analysis	Data analyses were performed using the following software: R version 3.6.3 (R package 'cause' version 1.0.0.267, 'gsmr' version 1.0.9, 'TwoSampleMR' version 0.5.5, and 'Seurat' version 3.2.2), PLINK version 1.90beta, Python version 2.7.16 (python module 'LDSC' version 1.0.0, 'HESS' version 0.5.3, 'mtag' version 1.0.8), SMR version 1.02 and CPASSOC version 1.0.1.

For manuscripts utilizing custom algorithms or software that are central to the research but not yet described in published literature, software must be made available to editors and reviewers. We strongly encourage code deposition in a community repository (e.g. GitHub). See the Nature Research guidelines for submitting code & software for further information.

Data

Policy information about availability of data

All manuscripts must include a data availability statement. This statement should provide the following information, where applicable:

- Accession codes, unique identifiers, or web links for publicly available datasets
- A list of figures that have associated raw data
- A description of any restrictions on data availability

GWAS summary statistics for MS are available by application from https://imsgc.net/?page_id=31. GWAS summary statistics for IBD, UC, and CD are publicly available from https://www.ebi.ac.uk/gwas/publications/26192919. GTEx expression summary data are available from https://gtexportal.org/home/datasets. Summary-level scRNA-seq data are available from https://singlecell.broadinstitute.org/single_cell/study/SCP44/small-intestinal-epithelium for mouse small intestine, https://www.tissuestabilitycellatlas.org/ for human lung and spleen, and https://support.10xgenomics.com/single-cell-gene-expression/datasets for human PBMC. The eQTL summary data for eQTLGen and GTEx are available from https://www.eqtlgen.org/cis-eqtls.html and https://cnsgenomics.com/software/smr/#DataResource. Source data are provided with this paper.

Field-specific reporting

Please select the one below that is the best fit for your research. If you are not sure, read the appropriate sections before making your selection.

- [x] Life sciences
- [ ] Behavioural & social sciences
- [ ] Ecological, evolutionary & environmental sciences

For a reference copy of the document with all sections, see nature.com/documents/nr-reporting-summary-flat.pdf

Life sciences study design

All studies must disclose on these points even when the disclosure is negative.

Sample size	All our analyses were based on publicly-available data. The sample sizes in our analyses represent the total data currently available.
Data exclusions	We applied a number of exclusion criteria for the purpose of quality control. For GWAS datasets, we excluded SNPs with minor allele frequency <1% and SNPs that were strand-ambiguous. For GTEx data, we excluded low-quality individuals (N=2, defined as <100 genes with >1 reads per million) and genes (N=736, defined as <4 individuals with >1 reads per million). For human scRNA-seq data, we excluded genes if they were coded with a non-unique gene identifier, were not expressed in any cell type, were non-protein coding genes, or were located in the MHC region. For mouse scRNA-seq data, we further excluded any gene that did not have a matching human gene.
Replication	As we used the largest GWAS summary results, no other datasets are available for replication.
Randomization	This is not relevant to our study as it is not a randomized controlled trial. However, the Mendelian randomization analyses we presented are based on the assumption that the segregation of alleles during meiosis is random.
Blinding	This is not relevant to our study as it is not a randomized controlled trial.

Reporting for specific materials, systems and methods

We require information from authors about some types of materials, experimental systems and methods used in many studies. Here, indicate whether each material, system or method listed is relevant to your study. If you are not sure if a list item applies to your research, read the appropriate section before selecting a response.

Materials & experimental systems

n/a	Involved in the study	
x		Antibodies
x		Eukaryotic cell lines
x		Palaeontology and archaeology
x		Animals and other organisms
x		Human research participants
x		Clinical data
x		Dual use research of concern

Methods

n/a	Involved in the study	
x		ChIP-seq
x		Flow cytometry
x		MRI-based neuroimaging
